# Cpeb1 remodels cell type–specific translational program to promote fear extinction

**DOI:** 10.1126/sciadv.adr8687

**Published:** 2025-01-10

**Authors:** Juan Zhang, Chun-Qing Yang, Zhi-Qiang Liu, Shi-Ping Wu, Zu-Guang Li, Luo-Man Zhang, Hong-Wei Fan, Zi-Yuan Guo, Heng-Ye Man, Xiang Li, You-Ming Lu, Ling-Qiang Zhu, Dan Liu

**Affiliations:** ^1^ Department of Pathophysiology, School of Basic Medicine, Tongji Medical College, Huazhong University of Science and Technology, Wuhan, Hubei 430030, China.; ^2^ Department of Pathology, The First Affiliated Hospital of Zhengzhou University, Zhengzhou 450002, China.; ^3^ Center for Stem Cell and Organoid Medicine (CuSTOM), Division of Developmental Biology, Cincinnati Children's Hospital Medical Center, Cincinnati, OH 45229, USA.; ^4^ Department of Biology, Boston University, Boston, MA 02215, USA.; ^5^ Department of Neurosurgery, Zhongnan Hospital of Wuhan University, Wuhan, China.; ^6^ Brain Research Center, Zhongnan Hospital of Wuhan University, Wuhan, China.; ^7^ Medical Research Institute, Wuhan University, Wuhan, Hubei 430030, China.; ^8^ Department of Medical Genetics, School of Basic Medicine, Tongji Medical College, Huazhong University of Science and Technology, Wuhan, Hubei 430030, China.

## Abstract

Protein translation is crucial for fear extinction, a process vital for adaptive behavior and mental health, yet the underlying cell-specific mechanisms remain elusive. Using a Tet-On 3G genetic approach, we achieved precise temporal control over protein translation in the infralimbic medial prefrontal cortex (*IL*) during fear extinction. In addition, our results reveal that the disruption of cytoplasmic polyadenylation element binding protein 1 (Cpeb1) leads to notable alterations in cell type–specific translational programs, thereby affecting fear extinction. Specifically, Cpeb1 deficiency in neurons activates the translation of heterochromatin protein 1 binding protein 3, which enhances microRNA networks, whereas in microglia, it suppresses the translation of chemokine receptor 1 (*Cx3cr1*), resulting in an aged-like microglial phenotype. These coordinated alterations impair spine formation and plasticity. Our study highlights the critical role of cell type–specific protein translation in fear extinction and provides an insight into therapeutic targets for disorders with extinction deficits.

## Teaser

Cpeb1 promotes fear extinction by inhibiting neuronal Hp1bp3 translation and activating microglial Cx3cr1 translation.

## INTRODUCTION

Fear extinction learning is an evolutionarily conserved and flexible process crucial for an organism’s survival and its ability to appropriately respond to dangerous or aversive stimuli (*1*). In the classical Pavlovian fear conditioning paradigm, a widely used experimental model for studying fear extinction learning (*2*, *3*), this process unfolds as the conditioned stimulus (CS) is repeatedly presented in the absence of the unconditioned stimulus (US), prompting the organism to swiftly reverse previously acquired contingencies (*4*). Dysfunctions in fear extinction learning have been associated with various neuropsychiatric conditions. For instance, individuals with posttraumatic stress disorder (PTSD) often exhibit decreased fear extinction abilities, leading to heightened fear responses to trauma-related cues (*5*). Similarly, those grappling with anxiety and addiction withdrawal frequently experience challenges with extinction learning, perpetuating cycles of anxiety and substance use (*6*). Currently, the primary effective treatment for these disorders is extinction-based exposure therapy, which mitigates fear responses by subjecting individuals to repeated exposure to feared stimulus in a safe environment (*7*). Consequently, a deeper comprehension of fear extinction learning holds promise for developing innovative therapeutic approaches tailored to individuals afflicted by these disorders, thereby enhancing their daily functioning and overall quality of life.

Protein synthesis is a fundamental cellular process used by cells to respond to signals originating from both inside and outside the cell, enabling the reshaping of cell function (*8*) and facilitating the formation of new neural connections, as well as the restructuring of existing ones (*9*). Deregulation of protein translation plays a vital role in memory by affecting protein expression, function, and cellular homeostasis. Protein translation dysfunction primarily stems from disruption in the three key processes of translation initiation, elongation, and termination (*10*). For example, knockdown of neuronal *eIF4E* knockdown in the lateral amygdala, leading to inhibition of the neuronal translation initiation process, has been shown to impair memory consolidation (*11*). Conversely, phosphomutation of the translation initiation factor *eIF2*α in excitatory or somatostatin-expressing inhibitory neurons enhances overall mRNA translation, thereby strengthening synaptic plasticity and improving long-term memory (*12*). Some drugs, such as anisomycin, cycloheximide, and rapamycin, impair memory consolidation by inhibiting the translation elongation (*13*–*15*). Moreover, protein translation is crucial for the consolidation of the extinction memory, particularly in the infralimbic subregion (*IL*) of the prefrontal cortex (*9*). Previous studies have demonstrated that infusion of the protein translation inhibitor anisomycin into the *IL* during extinction training not only reduced the activity of medial prefrontal cortex (mPFC) neurons but also notably hindered fear extinction in rats (*16*). Similarly, extinction learning with social support was impaired by injecting the protein synthesis inhibitors, such as anisomycin and rapamycin, into the *IL* during extinction training (*17*). Extinction of conditioned taste aversion was impeded by microinfusing anisomycin into the *IL* immediately following a nonreinforced extinction session (*18*). These findings suggest that deregulation of the protein translation process is closely associated with impaired extinction memory, and the extinction process may be compromised in the absence of proper protein synthesis in the *IL*. However, little is known about the translation function in specific cell populations, and the underlying mechanism of cell type–specific protein translation in fear extinction remains to be elucidated.

In this study, the genetically encodable protein synthesis inhbitor (gePSI) and Tet-on 3G system were used to inhibit protein synthesis in the IL. It was observed that fear extinction learning was impaired because of disruption of protein translation. Subsequently, a fear extinction learning protocol was implemented (*19*), and screening of mice was conducted to identify groups with Impaired-extinction (Impaired-ext) and Normal-extinction (Normal-ext) using the principal components analysis (PCA) method. Our findings, combined with the proteomic and transcriptomic data, indicated that posttranscriptional translational regulation plays a critical role in fear extinction. Furthermore, we demonstrated that the involvement of neuron- and microglia-specific translational profile driven by cytoplasmic polyadenylation element binding protein 1 (Cpeb1) simultaneously. Specifically, the translational activation of *Hp1bp3* in neurons promoted a complex network of microRNAs (miRNAs) to regulate the disruption of postsynaptic proteins, hindering spine formation and plasticity, while the suppression of translation of *Cx3cr1* disturbed the homeostasis in microglia, resulting in an aged phenotype and impairing their capability to engulf the extracellular matrix (ECM) Aggrecan, thereby promoting the ECM deposition and reduction of the dendritic spine. Overall, our findings suggest that the reduction of Cpeb1 remodels the neuron- and microglia-specific translational program, leading to the disruption of synaptic structure and plasticity, ultimately impairing fear extinction.

## RESULTS

### The protein translation programs are required for fear extinction

**Fig. 1.**
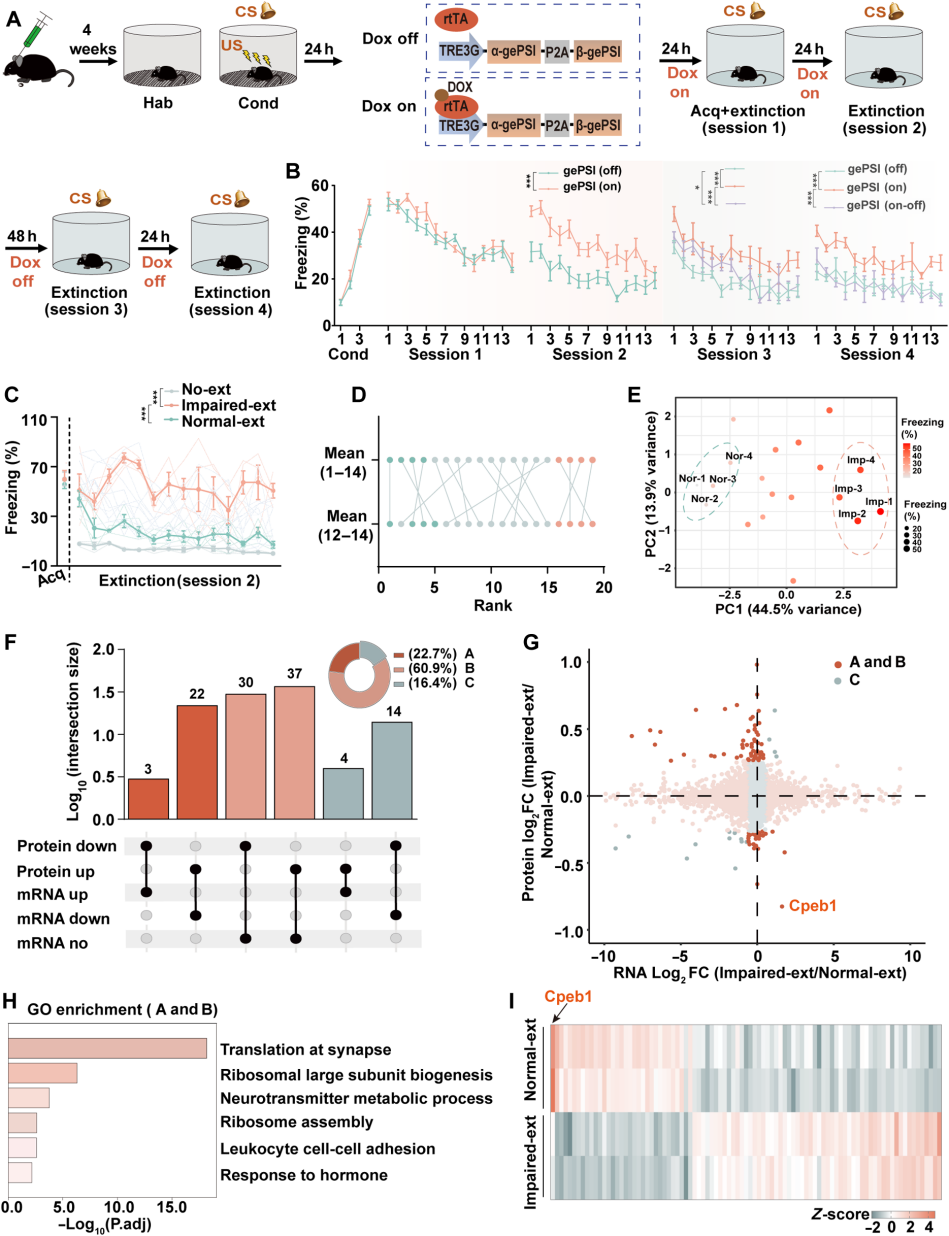
The protein translation programs are required for fear extinction. (**A**) Schematic diagram of the behavioral process for fear conditioning training, Tet-on system, and fear extinction experiment. (**B**) Percentage of freezing levels is shown in blocks of two sessions across all phases of the experiment (trial 4 during conditioning and trial 14 during extinction). Detailed experimental procedures are shown in the “Behavioral assays” section of Materials and Methods (*P < 0.05, ***P < 0.001, *n* = 5 to 10). (**C**) Percentage of freezing level in fear memory acquisition (Acq) and average percentage of freezing of 14 trials in the second stage of extinction learning from No-ext, Normal-ext, and Impaired-ext group mice (***P < 0.001, *n* = 21). (**D**) The average percentage of freezing was ranked in the 14 trials during the second stage of extinction learning compared to the percentage of freezing in the last three trials for the mice. (**E**) PCA for the average percentage of freezing in the 14 trials during the second stage of extinction learning (*n* = 19; Nor, Normal-ext; Imp, Impaired-ext). (**F** and **G**) Analysis of proteome and transcriptome on Impaired-ext and Normal-ext group. Proteome and transcriptome comparison between them was performed in (F). Scatter plot of the fold change of RNA and protein comparing Impaired-ext to Normal-ext is shown in (G) (mRNA, fold change >1.5; Protein, fold change >1.2; group A exhibits an inverse correlation between protein and mRNA levels; group B represents protein levels change while mRNA remains constant; group C represents a positive correlation between protein and mRNA levels). (**H**) Functional enrichment analysis of gene populations from groups A and B in (F) or (G) (fold change >1.5, *P* < 0.05, *n* = 3 per group). (**I**) Heatmap showing the expression profiles of protein levels (groups A and B) between Impaired-ext and Normal-ext mice (fold change >1.2, *P* < 0.05, *n* = 2 per group).

To ascertain whether fear extinction memory depends on protein translation in the IL, the gePSI and Tet-on 3G systems were used to achieve temporal regulation of protein synthesis (*20*). Immunofluorescence and surface sensing of translation (SUnSET) assay (*21*) effectively demonstrated the inhibition of translation in the IL (fig. S1, A to F). Subsequently, mice injected with gePSI-expressing virus or control virus in the IL were subjected to a fear extinction paradigm, the doxycycline (Dox) and Tet-on 3G system were introduced to inhibit the protein translation at any time. Our results suggested that the gePSI-expressing group with Dox treatment (gePSI-On) did not impair the acquisition of fear memory but exhibited a high freezing level compared to the gePSI-expressing group without Dox treatment (gePSI-Off) and control group (Con-On, Con-Off) during fear extinction (Fig. 1, A and B, and fig. S1G). In addition, no disparity in freezing levels during fear extinction between control mice with (Con-On) or without Dox (Con-Off) was observed (fig. S1G). Moreover, in the gePSI (On-Off) mice, fear extinction inflexibility was reversed when the Dox administration was stopped (Fig. 1, A and B, and fig. S1, H and I). Thus, transient protein synthesis inhibition before extinction learning blocked the shift from retrieving the original fear memory to initiating the formation of a new fear-extinction memory, suggesting that the protein translation is necessary for fear extinction flexibility.

To identify the critical translational events associated with impaired fear extinction, we first used a classic example of the Pavlovian fear conditioning protocol and identified the Impaired-ext group and the Normal-ext group by analyzing the mean percentage of freezing across the 14 trials in session 2 of extinction learning, as well as the average freezing percentage during the last three trials. PCA, as referenced in our previous report (*19*), further validated the feasibility of our classification on the basis of the freezing levels observed during session 2. With this approach, we selected two proportions of mice that displayed the quite different freezing time. We therefore named these two groups as Impaired-ext and Normal-ext groups (Fig. 1, C to E, and fig. S2A). We also included a control group, which did not receive CS stimuli during extinction learning, named as No-extinction (No-ext) (fig. S2B). It is known that the mPFC played an important role in fear extinction (*22*), so we then performed quantitative proteomics and transcriptomic analysis in the mPFC of Impaired-ext, Normal-ext, and No-ext groups (fig. S2C). Proteomes in different groups were compared, and functional enrichment analysis was conducted on differentially expressed proteins (fold change > 1.2, *P* < 0.05). We observed that the pathways enriched in the Impaired-ext group were mainly related to synaptic translation and ribosome assembly pathway, which did not show notable enrichment between the Normal-ext and No-ext groups (fig. S2, D to G). These data suggested that some specific protein translation programs may play an important role in impaired fear extinction.

To further identify the specific translational programs implicated in fear extinction, we compared the proteomic and transcriptomic characteristics between the Normal-ext and Impaired-ext groups. All the matched genes/proteins were categorized into three categories: Group A exhibits an inverse correlation between protein and mRNA levels. Group B shows changes in protein levels while mRNA remains constant. Group C displays a positive correlation between protein and mRNA levels. We found that the proportion of genes in groups A and B was much higher than in group C (Fig. 1, F and G). Functional enrichment analysis was conducted on the genes in groups A and B (fold change >1.2, *P* < 0.05), revealing notable enrichment in pathways such as “translation” and “ribosome biogenesis.” Combined with the discordant proteomic and transcriptomic signatures, we propose that translational regulation mechanisms may play an important role in the impaired extinction learning (Fig. 1H). Among the changed proteins in groups A and B (fold change >1.2, *P* < 0.05), Cpeb1 is particular of interest, because (i) Cpeb1 plays important role in the posttranscriptional translation regulation (*23*); (ii) Cpeb1 was significantly down-regulated in Impaired-ext mice, especially in the *IL* subregions of the mPFC (Fig. 1I and fig. S3, A to D); and (iii) Cpeb1 was implicated in the reduced extinction of hippocampal-dependent memories (*24*).

### Cell type–specific Cpeb1-dependent translational signature are necessary for fear extinction

**Fig. 2.**
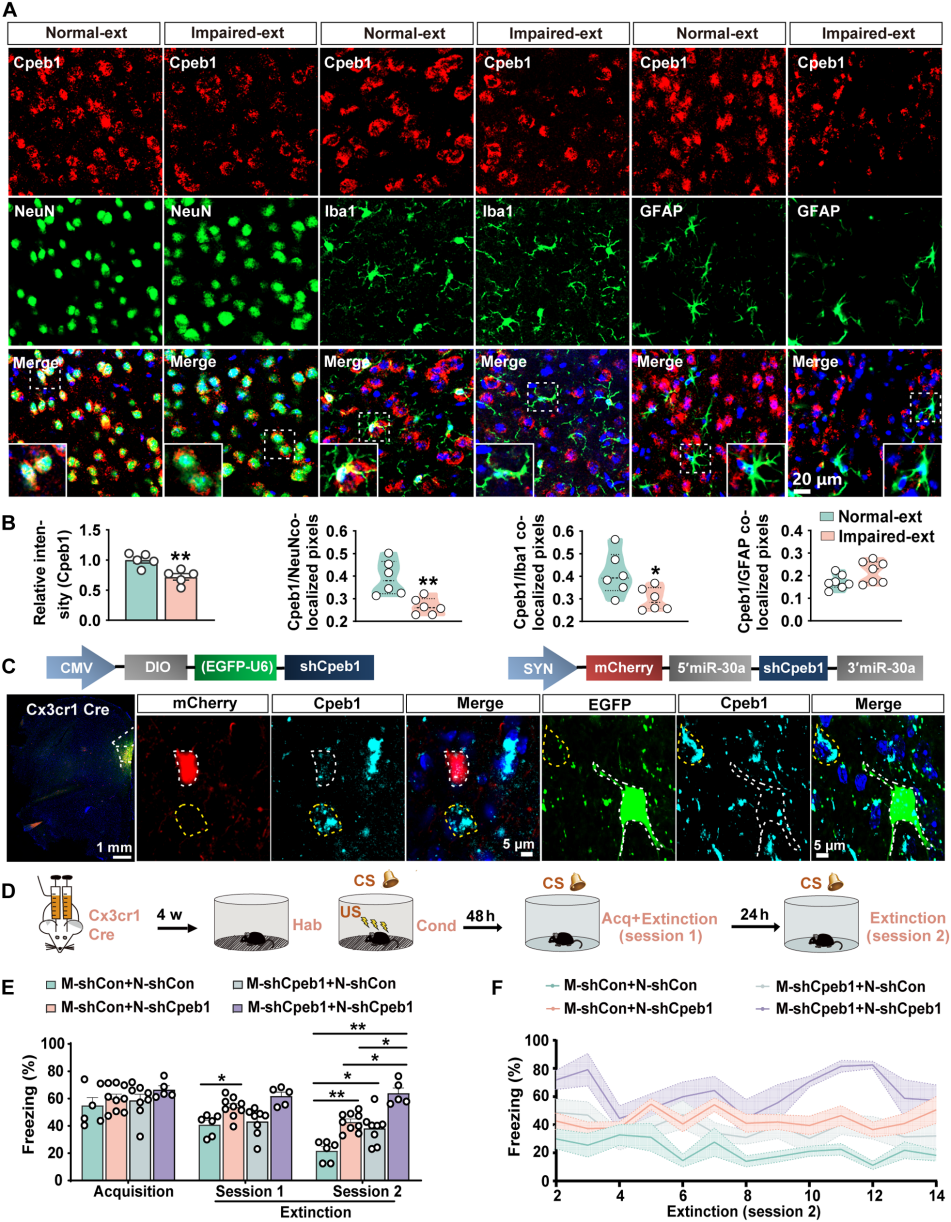
Cpeb1-dependent translational signatures in neuron and microglia are crucial for fear extinction. (**A** and **B**) Immunofluorescence staining was performed in the *IL* region from Impaired-ext and Normal-ext groups by using anti-Cpeb1 (red) and Neun, Iba1, and glial fibrillary acidic protein (GFAP) (green) antibodies. The representative images are shown in (A), and relative intensity of Cpeb1 and the colocalized pixels in different cell populations were calculated in (B) (*P < 0.05, **P < 0.01, *n* = 6 cells from five mice per group). (**C**) Representative confocal image (right) for virus injection into *IL* of the mPFC from *Cx3cr1*-*Cre* mice. Coronal slices from virus infected (mCherry) neurons were stained with anti-Cpeb1 (cyan) antibody, and coronal slices from virus infected (EGFP) microglia cells were stained with anti-Cpeb1 (cyan) antibody. White dashed lines indicate neurons or microglia cells with virus infection, and yellow dashed lines indicate neurons or microglia cells without virus infection. (**D**) Schematic illustration of the workflow for the fear conditioning training and fear extinction learning assay after viral intervention of *Cx3cr1*-*Cre* mice. Hab, habitation; Cond, conditioning; Acq, acquisition. (**E**) Average freezing percentage of fear memory acquisition and average freezing percentage in the first and second sessions of fear extinction learning from M-shCon+N-shCon, M-shCon+N-shCpeb1, M-shCpeb1+N-shCon, and M-shCpeb1+N-shCpeb1 groups (*P < 0.05, **P < 0.01, *n* = 5 to 9 per group). (**F**) Percentage of freezing of 14 trials in the second session of extinction learning from M-shCon+N-shCon, M-shCon+N-shCpeb1, M-shCpeb1+N-shCon, and M-shCpeb1+N-shCpeb1 groups (*n* = 5 to 9 per group).

We then tried to understand the cell type–specific changes of Cpeb1 among different groups. To this end, we performed double-immunofluorescence staining using antibodies to Cpeb1 and different cell markers and found that the decrement of Cpeb1 was mainly localized in the neurons and microglial rather than in astrocytes (Fig. 2, A and B). We subsequently investigated the specific cell type in which the down-regulation of *Cpeb1* is involved in fear extinction. We injected neuronal promoter–driven shCpeb1 virus (AAV/9-hSyn-mCherry-5′miR-30a-shCpeb1-3′miR-30a, N-shCpeb1) and the adeno-associated virus (AAV) vectors containing the double-floxed inverse orientation (DIO) element with a short hairpin RNA (shRNA) (shCpeb1) (AAV2/6-CMV-DIO-EGFP-U6-shCpeb1, M-shCpeb1) into the *IL* of *Cx3cr1*-*Cre* mice (Fig. 2C and fig. S4, A and B) to knock out the *Cpeb1* gene in neuron and microglia, respectively. Obviously, the intensity of Cpeb1 was noticeably decreased in the mCherry-positive neurons and the enhanced green fluorescent protein (EGFP)–positive microglia of *IL*, confirming the effectiveness of the shRNA-based knockdown strategies (Fig. 2C). Nissl staining revealed that neuron- and microglia-specific knockouts of *Cpeb1* did not result in any substantial changes in cell density (fig. S4, C and D). We then subjected these mice to the fear conditioning and fear extinction learning (Fig. 2D). We found that all mice exhibited comparable freezing behavior during the acquisition stage of cued fear memory (Fig. 2E). However, silencing of *Cpeb1* in either neuron or microglia led to a higher freezing level during the extinction stage (Fig. 2E). Knocking out *Cpeb1* in neurons showed higher freezing levels than knocking out *Cpeb1* in microglia during the initial phase of extinction learning (Fig. 2E). Furthermore, mice with Cpeb1 attenuation in neurons and microglia simultaneously displayed much higher freezing levels in the second phase of extinction learning stage (Fig. 2, E and F). Knockdown of *Cpeb1* in the prelimbic region (*PL*) did not find any deficits in the fear extinction, suggesting the crucial role of IL-specific Cpeb1 down-regulation in fear extinction (fig. S4, E to I). No substantial changes were found in locomotor and anxiety-like behavior in these mice when subjected to the open-field test and elevated plus maze task test (fig. S4, J to L). Collectively, these data suggested that both neuronal and microglial Cpeb1 were critical for fear extinction.

### Cell type–specific downstream effectors of Cpeb1 regulate fear extinction

**Fig. 3.**
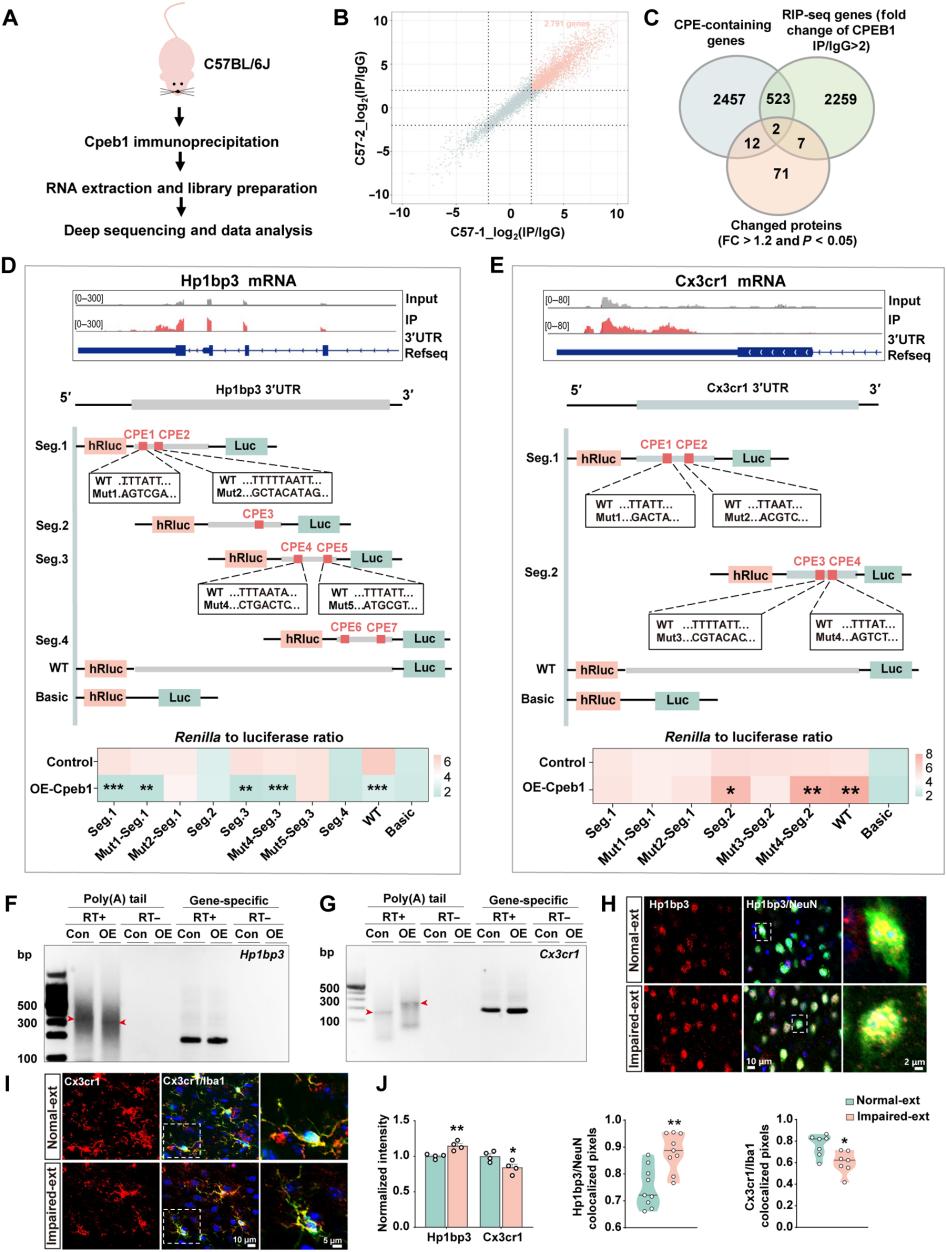
Distinct downstream effectors of Cpeb1 in neuron and microglia regulate fear extinction. (**A**) Schematic outlining the Cpeb1 RNA immunoprecipitation sequencing (RNA-IP-seq) workflow from C57BL/6 J mice (*n* = 2 per group). (**B**) Scatter plot displaying genes from two independent Cpeb1 RNA immunoprecipitation experiments. Each dot represents one gene, with *x* and *y* axes indicating fold changes of IP/IgG for each Cpeb1 RNA immunoprecipitation experiment. (**C**) Venn diagram illustrating the overlap between CPE-containing genes, RNA-IP-seq genes (fold change of IP/IgG > 2), and proteins identified by mass spectrometry (fold change >1.2, *P* < 0.05). (**D** and **E**) Cpeb1 RNA-IP-seq data for *Hp1bp3* [top of (D)] and *Cx3cr1* [top of (E)] transcripts. 3′UTRs of *Hp1bp3* and *Cx3cr1*, with WT or mutated CPEs, were inserted downstream of *Renilla* luciferase (hRluc) gene. These constructs were cotransfected into 293 T cells with the empty vetor pcDNA3.1(+) or pcDNA3.1(+)-*Cpeb1*, along with a firefly luciferase (Luc) vector as an internal control. After 48 hours, luciferase activity was measured using a dual-luciferase assay. Schematic illustration of WT or mutated CPEs in 3′UTRs of *Hp1bp3* [middle of (D)] and *Cx3cr1* [middle of (E)] are provided, and the corresponding luciferase activity [bottom of (D) and (E)] are shown (*P < 0.05, **P < 0.01, ***P < 0.001, *n* = 3 replications). (**F** and **G**) HT22 or BV2 cells were transfected with either empty vector (Con) or overexpress *Cpeb1* (OE). After 48 hours, RNA was isolated and subjected to PCR poly(A) test. (F) Bands for *Hp1bp3* poly(A) tail and primers. (G) *Cx3cr1* poly(A) tail and primers (RT+, reverse transcription; RT−, no RT; *n* = 3 replications). Arrowheads indicate poly(A) PCR product. (**H** to **J**) Immunofluorescence of IL sections from Impaired-ext and Normal-ext mice using anti-Hp1bp3/anti-Cx3cr1 (red) (H) and anti-Neun/anti-Iba1 (green) (I) antibodies. Representative confocal images are provided. Normalized intensities of Hp1bp3 and Cx3cr1, Hp1bp3/Neun, and Cx3cr1/Iba1 colocalized pixels were calculated (J) (*P < 0.05, **P < 0.01, *n* = 7 to 9 cells from four mice per group).

We then wanted to identify specific Cpeb1-dependent translational events in impaired fear extinction. It is known that Cpeb1 regulates cellular function by controlling the translation of specific transcripts (*25*). To identify the downstream targets of Cpeb1 mediating fear extinction learning in neurons and microglia separately, we purified RNA from the pellet of mPFC homogenates from wild-type (WT) C57BL/6 J mice immunoprecipitated by a Cpeb1 antibody (fig. S5A) and subjected it to *RNA* sequencing (Fig. 3A). Compared to immunoglobulin G (IgG)–immunoprecipitated pellets, 2791 transcripts were enriched in the anti–Cpeb1-immunoprecipitated pellets (fold change >2, *P* < 0.05) (Fig. 3B). Because Cpeb1 binds to specific transcriptomes via a cytoplasmic polyadenylation element (CPE) (*26*), we further analyzed these 2791 transcripts and identified 525 genes containing CPEs. Subsequently, we merged the 525 Cpeb1-associated genes with our proteomic data and identified two genes: *Hp1bp3* and *Cx3cr1* (Fig. 3C).

Heterochromatin protein 1 binding protein 3 (Hp1bp3), recognized as a previously unidentified cognitive function modulator (*27*) and Cx3cr1, a cell surface receptor predominantly expressed on microglia (*28*), are both known to play pivotal roles in synaptic plasticity and cognitive function (*29*, *30*). Subsequently, we sought to determine whether Cpeb1 indeed regulated the translation of *Hp1bp3* and *Cx3cr1*. We first verified that both *Hp1bp3* and *Cx3cr1* transcripts were enriched in Cpeb1-immunoprecipitated pellets (Fig. 3, D and E). We then identified seven and four canonical CPE sites in the 3′ untranslated region (3′UTR) of *Hp1bp3* and *Cx3cr1* mRNAs, respectively, through bioinformatics analysis (Fig. 3, D and E). To further clarify the specific CPE that biologically regulated by Cpeb1 in these two targets, we constructed a series of luciferase reporters that contains different CPEs in *Hp1bp3* (Seg.1: contains CPE1 and CPE2; Mut1-Seg.1: contains mutated CPE1 and WT CPE2; Mut2-Seg.1: contains mutated CPE2 and WT CPE1; Seg2: contains CPE3; Seg3: contains CPE4 and CPE5; Mut4-Seg.3: contains mutated CPE4 and WT CPE5; Mut5-Seg.3: contains mutated CPE5 and WT CPE4; Seg4: contains CPE6 and CPE7) and *Cx3cr1* (Seg1: contains CPE1 and CPE2; Mut1-Seg.1: contains mutated CPE1 and WT CPE2; Mut2-Seg.1: contains mutated CPE2 and WT CPE1; Seg2: contains CPE3 and CPE4; Mut3-Seg.2: contains mutated CPE3 and WT CPE4; Mut4-Seg.2: contains mutated CPE4 and WT CPE3) separately. Then, we transfected these reporters with pcDNA3.1(+)-*Cpeb1* construct into the human embryonic kidney–293T cells and performed the luciferase analysis. We found that overexpression of Cpeb1 significantly reduced the luciferase activity in the Seg1 and Seg3 but not CPE2-mutated Seg1 (Mut2-Seg.1) and CPE5-mutated Seg3 (Mut5-Seg.3) of *Hp1bp3* constructs. Meanwhile, Cpeb1 enhanced the luciferase activity of WT Seg2 but not CPE3-mutated Seg2 of *Cx3cr1* constructs (Fig. 3, D and E). These data suggested that CPE2 and CPE5 in *Hp1bp3*, CPE3 in *Cx3cr1*, are key binding motifs of Cpeb1. As reported, Cpeb1 is able to bind with target mRNAs with CPEs, thereby activating or suppressing their translation by influencing polyadenylate [poly(A)] tail elongation and removal (*25*). To examine the polyadenylation state of *Hp1bp3* and *Cx3cr1*, we used a poly(A) tail-length assay. Following poly(G/I) tailing and reverse transcription, the poly(A) polymerase chain reaction (PCR)–amplified products from Cpeb1 overexpression displayed a shorter length of the poly(A) tail for *Hp1bp3* transcript but a longer length for *Cx3cr1* transcript (Fig. 3, F and G, and fig. S5, B and C). Moreover, compared to the control group, *Cpeb1* overexpression in HT22 cells led to an approximate twofold reduction in Hp1bp3 protein levels. Conversely, knockdown of *Cpeb1* in HT22 cells resulted in a nearly 1.5-fold increase in Hp1bp3 protein expression. In BV2 microglial cells, *Cpeb1* overexpression caused an approximate 1.3-fold elevation in Cx3cr1 protein levels, whereas silencing *Cpeb1* led to a nearly 2-fold decrease in Cx3cr1 protein expression. Neither overexpression nor silencing of Cpeb1 affected the transcription of *Hp1bp3* and *Cx3cr1* (fig. S6, A to F). Collectively, these data suggest that Cpeb1 regulates *Hp1bp3* and *Cx3cr1* translation by binding specific CPEs and affecting the poly(A) tail elongation. Having verified the direct translational regulation of *Hp1bp3* and *Cx3cr1* by Cpeb1, we then queried whether Hp1bp3 and Cx3cr1 are indeed involved in the impaired fear extinction that affected by Cpeb1. Immunofluorescence experiments suggest that Hp1bp3 is mainly localized in neurons but not microglia, while Cx3cr1 is predominantly distributed in microglia but not neurons (Fig. 3, H to J, and fig. S6, G to J). In the Impaired-ext mice, the protein level of Hp1bp3 is elevated while Cx3cr1 is reduced, without affecting *Hp1bp3* and *Cx3cr1* mRNA levels (fig. S6, K to M). Therefore, it is possible that Hp1bp3 and Cx3cr1 are independent downstream effectors of Cpeb1 in neurons and microglia to mediate fear extinction inflexibility.

### Cell type–specific Cpeb1-dependent translational signature is necessary for synaptic maturation

**Fig. 4.**
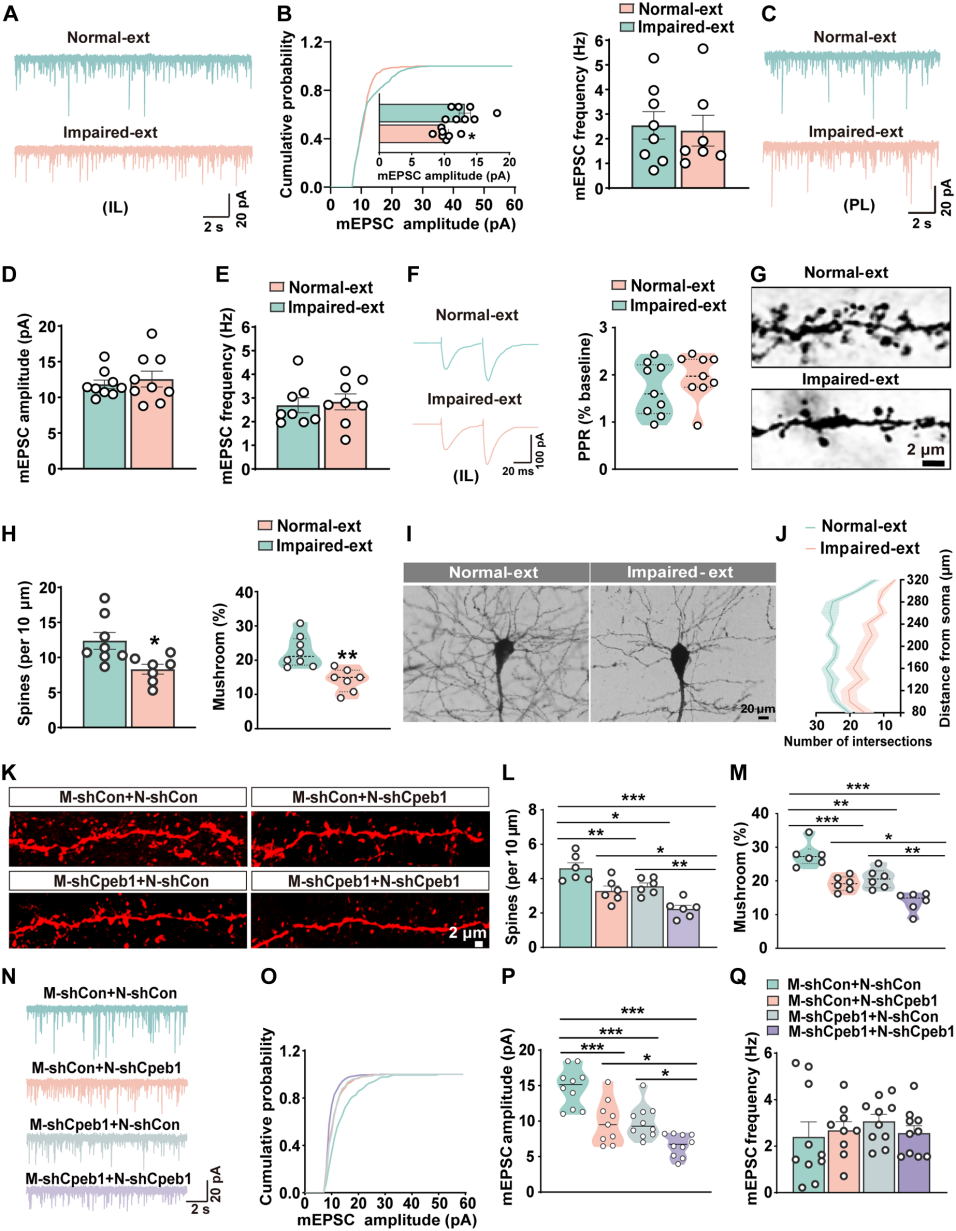
Neuron- and microglia-specific Cpeb1-dependent translational signatures are necessary for synaptic function. (**A** and **B**) Whole-cell patch-clamp recordings of *IL* neurons from Normal-ext and Impaired-ext mice. Representative mEPSC traces are shown (A), with quantification of mEPSC amplitude and frequency (B) (*P < 0.05, *n* = 7 to 8 neurons from four mice per group). (**C** to **E**) Whole-cell patch-clamp recordings of PL neurons from Normal-ext and Impaired-ext mice. Representative mEPSC traces are shown (C), with quantification of mEPSC amplitude (D) and frequency (E) (*n* = 8 to 9 neurons from four mice per group). (**F**) Paired-pulse ratios recordings of IL neurons from Normal-ext and Impaired-ext mice. mEPSC traces are shown in the right panel, with quantification of EPSC amplitude on the left (*n* = 9 neurons from four mice per group). (**G** and **H**) Golgi staining of *IL* sections from Impaired-ext and Normal-ext groups. Representative images are shown (G), with quantification of spine density and mushroom spine percentage (H) (*P < 0.05, **P < 0.01, *n* = 7 to 8 slices from four mice per group). (**I**) Golgi staining images showing the dendritic trees from Impaired-ext and Normal-ext groups. (**J**) Sholl analysis evaluating dendritic complexity in neurons from the Impaired-ext and Normal-ext groups (*n* = 8 to 10 neurons from four mice per group). (**K** to **M**) Dendritic spine in *IL* neurons labeled with AAV9-hSyn-mCherry and AAV9-hSyn-mCherry-shcpeb1 viruses from *Cx3cr1*-*Cre* mice with the neuron-specific, microglia-specific, and combined *Cpeb1* deletion. Representative confocal images of spines are shown in (K), with quantitative analysis of spine density (L) and mushroom spine percentage (M) (*P < 0.05, **P < 0.01, ***P < 0.001, *n* = 6 neurons from four mice per group). (**N** to **Q**) Whole-cell patch-clamp recordings of *IL* neurons from *Cx3cr1*-*Cre* mice with neuron-specific, microglia-specific, and combined *Cpeb1* deletion. Representative mEPSC traces are shown in (N), with quantification of mEPSC amplitude (O and P) and frequency (Q) (*P < 0.05, ***P < 0.001, *n* = 7 to 10 neurons from four mice per group).

We then wanted to explore the underlying molecular mechanisms contributing to impaired fear extinction induced by Cpeb1-dependent translational signature in neurons and microglia. By conducting the functional enrichment analysis on the 525 Cpeb1-associated genes (fig. S7A), we found that they were involved in synapse-related pathways such as “Synapse organization,” “Axonogenesis,” “Dendrite development,” and “Learning or memory” (fig. S7B), confirming the critical role of Cpeb1 in synaptic plasticity during fear extinction, aligning with our proteomic findings. Given the role of Cpeb1-dependent translational signature in regulating synaptic plasticity, we hypothesized that the down-regulation of Cpeb1 in both neurons and microglia might perturb synaptic structure and function, thereby impairing fear extinction learning. Comparing miniature excitatory postsynaptic currents (mEPSCs) in mPFC neurons, we found a decrease in mEPSC amplitude, but not frequency, in the *IL* of the Impaired-ext group without affecting the *PL*, suggesting the potential involvement of postsynaptic mechanisms (Fig. 4, A to E). The unchanged paired-pulse facilitation ratio further suggested intact presynaptic functions in the *IL* (Fig. 4F). Golgi staining revealed a significant decrease in both dendritic spine density and the percentage of matured spines in the Impaired-ext group (Fig. 4, G and H). In addition, Sholl analysis indicated a notable reduction in dendritic branches in the Impaired-ext group (Fig. 4, I and J). These findings strongly suggest that the postsynaptic function may play an important role in mediating the impaired fear extinction.

We then investigated whether specific knockout of *Cpeb1* in neurons and microglia could replicate the similar postsynaptic pathology as observed in Impaired-ext mice. We found that artificially knockdown of *Cpeb1* in neurons or microglia resulted in decreased dendritic spine density, dendritic tree maturation, and mEPSC amplitude. Compared to the control virus–infected group (M-shCon+N-shCon), knockdown of *Cpeb1* in both neurons (M-shCon+N-shCpeb1) and microglia (M-sh Cpeb1+N-shCon) induced a more pronounced decline in the density of dendritic spines, the percentage of mushroom-type spines, and mEPSC amplitude (Fig. 4, K to Q, and fig. S8, A and B). These data strongly suggest that the disorder of cell type–specific Cpeb1-dependent translational signature impairs synaptic transmission and structure.

### Neuron-specific translational signal regulates synaptic function via a complex miRNA network

**Fig. 5.**
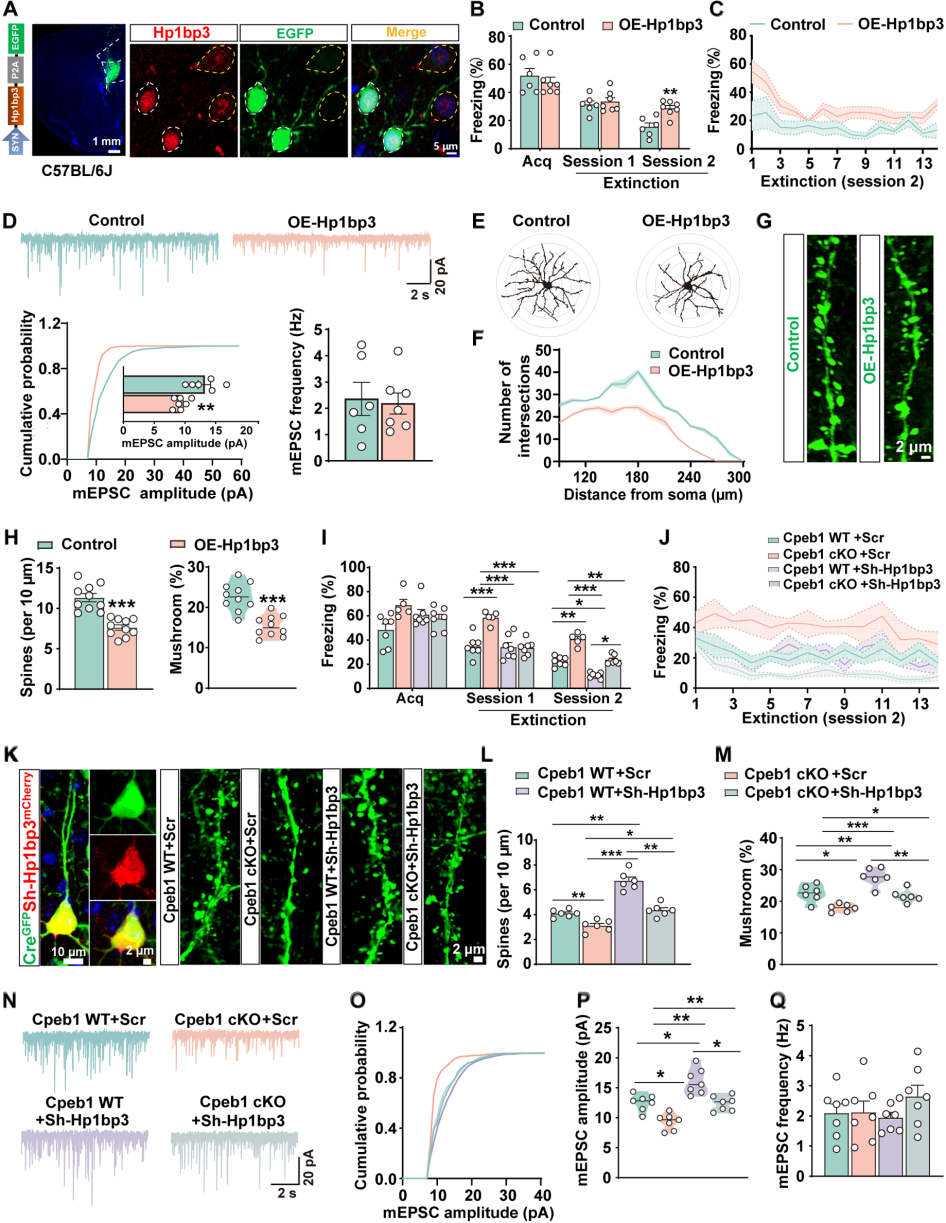
Neuronal Cpeb1-Hp1bp3 signaling regulates fear extinction by affecting synaptic function. (**A**) Confocal image for Hp1bp3- infected virus in the *IL* from C57BL/6 J mice (right), and image of Hp1bp3-infected neurons stained with anti-Hp1bp3 (red) is shown (left). White dashed lines mark virus-infected neurons, and yellow dashed lines mark noninfected ones. (**B**) Freezing percentages during fear memory acquisition and the first and second sessions of extinction learning were compared between control and OE-Hp1bp3 groups (**P < 0.01, *n* = 6 to 7 per group). (**C**) Freezing percentages across 14 trials in the second extinction session of control and OE-Hp1bp3 groups (*n* = 6 to 7 per group). (**D**) Whole-cell patch-clamp recordings of the *IL* neurons from OE-Hp1bp3 and control mice. Representative mEPSC traces (top), with quantified amplitude (left) and frequency (right) (**P < 0.01, *n* = 6 to 7 neurons from four mice per group). (**E** and **F**) 2D reconstructions of *IL* neurons from OE-Hp1bp3 and control mice (E), with Sholl analysis of dendritic complexity (F) (*n* = 5 neurons from four mice per group). (**G** and **H**) Dendritic spine density and mushroom spine percentage in IL neurons from the OE-Hp1bp3 and control groups. Representative images (G) and quantifications (H) are shown (***P < 0.001, *n* = 10 neurons from four mice per group). (**I**) Freezing percentages during fear memory acquisition and the first and second sessions of extinction learning were analyzed in Cpeb1 WT+Scr, Cpeb1 cKO+Scr, Cpeb1 WT+Sh-Hp1bp3, and Cpeb1 cKO+Sh-Hp1bp3 groups (*P < 0.05, **P < 0.01, ***P < 0.001, *n* = 5 to 7 per group). (**J**) Freezing percentages across 14 trials in the second extinction session in these groups (*n* = 5 to 7 per group). (**K** to **M**) Confocal images of dendritic spine in *IL* neurons from same groups (K), with quantified dendritic spine density (L) and mushroom spine percentage (M) (*P < 0.05, **P < 0.01, ***P < 0.001, *n* = 6 neurons from four mice per group). (**N** to **Q**) Representative mEPSC traces from these groups (N), with quantified amplitude (O and P) and frequency (Q) (*P < 0.05, **P < 0.01, *n* = 7 neurons from four mice per group).

To understand how neuron-specific translational signal affects the postsynaptic function, we generated a mouse model with neuron-specific *Hp1bp3* overexpression in the *IL* by injecting the AAV2/9-SYN-Hp1bp3-3×FLAG-P2A-EGFP virus into the *IL* of C57BL/6 J mice (OE-Hp1bp3), and control groups were injected with the control virus AAV2/9-SYN-3×FLAG-P2A-EGFP into the *IL* (Control). Immunofluorescence and Western blot data validated successful overexpression of Hp1bp3 in neurons (Fig. 5A and fig. S9A). The Western blot data indicated that the expression level of Hp1bp3 in the OE-hp1bp3 group was nearly double that observed in the control group. The molecular band corresponding to Hp1bp3 appeared ~3 kDa higher than that of the control group, attributable to the fusion of Hp1bp3 with the Flag tag (fig. S9, B and C). We then subjected these mice to fear extinction tasks and found that the OE-Hp1bp3 group displayed comparable freezing time to control mice (Control) during fear acquisition. However, during the second extinction stage, the freezing percentage in OE-Hp1bp3 mice was nearly double that of control mice, indicating impaired extinction flexibility (Fig. 5, B and C). Meanwhile, compared to control virus–infected neuron, *Hp1bp3* overexpression led to a significant reduction in the amplitude of mEPSCs, without affecting the frequency. This indicated a specific impairment in α-amino-3-hydroxy-5-methyl-4-isoxazolepropionic acid receptor (AMPAR)–dependent synaptic transmission. Furthermore, dendritic complexity, as quantified by Sholl analysis, was notably reduced following *Hp1bp3* overexpression. In addition, neurons infected with *Hp1bp3* showed decreased dendritic spine density and a substantial reduction in the proportion of mushroom-type spines, which are essential for synaptic strength and plasticity (Fig. 5, D to H). Together, these findings suggest that neuron-specific overexpression of Hp1bp3 disrupts synaptic transmission and structural integrity, paralleling the deficits observed in Impaired-ext mice. To understand whether *Hp1bp3* overexpression acts as the downstream effector of Cpeb1 loss in neuron, we administered a specific shRNA targeting *Hp1bp3* (Sh-Hp1bp3) into the IL of *Cpeb1^fl/fl^* mice (Cpeb1 WT) with *Cpeb1* specific knockout in neurons (Cpeb1 cKO) (fig. S9, D to F, injected with AAV2/9-Syn-Cre virus to *IL*) (fig. S9G). We examined whether inhibition of *Hp1bp3* could alleviate the extinction learning impairment caused by neuronal Cpeb1 loss. We found that Sh-Hp1bp3 not only attenuated the increase in Hp1bp3 due to neuronal Cpeb1 loss (fig. S9, H and I) but also restored the impaired fear extinction resulting from neuronal Cpeb1 loss (Fig. 5, I and J). In addition, Sh-Hp1bp3 ameliorated the dendritic spine loss and reduction of mEPSC amplitude in *Cpeb1* knockout neurons (Fig. 5, K to Q). On the contrary, overexpression of *Hp1bp3* in neurons disrupted the spine density and maturation and weakened the mEPSCs responses, especially the amplitude (Fig. 5, D to H). Knockdown *Hp1bp3* in WT mice also facilitated fear extinction (Fig. 5, I and J) and reinforced the spine density and maturation, as well as boosting the postsynaptic electric responses (Fig. 5, K to Q). These data strongly suggest that up-regulation of Hp1bp3 in neurons leads to the synaptic disorder, which mediates the impaired fear extinction induced by loss of Cpeb1.

**Fig. 6.**
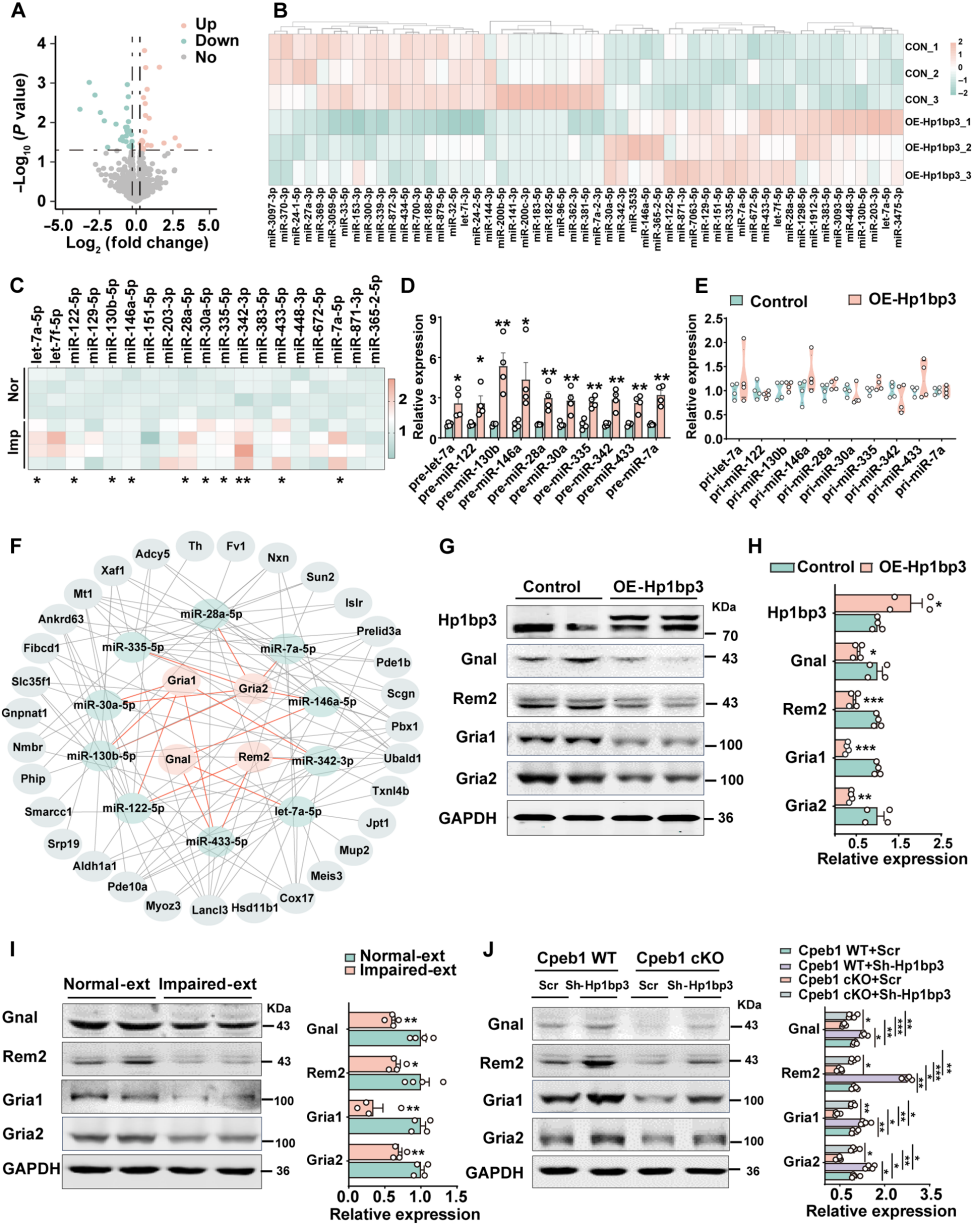
Neuronal Hp1bp3 signal regulates synaptic function via a complicated miRNA network. (**A**) Volcano plot showing the differentially regulated miRNAs in the mPFC from control and OE-Hp1bp3 groups with fold change >1.2 (pink dots) or <1.2 (cyan) and *P* value <0.05. The gray dots indicate the unchanged miRNAs (*n* = 3 per group). (**B**) Heatmap showing differential expression of miRNAs in the mPFC between control and OE-Hp1bp3 mice. (**C**) Quantitative PCR (qPCR) of 19 up-regulated miRNAs from control and OE-Hp1bp3 mice was performed in the mPFC between Impaired-ext (Imp) and Normal-ext (Nor) groups (*P < 0.05, **P < 0.01, *n* = 4). (**D** and **E**) qPCR was performed to measure the relative expression of pre-miRNA (D) and pri-miRNA (E) between control and OE-Hp1bp3 mice (*P < 0.05, **P < 0.01, *n* = 4). (**F**) An integrated analysis for the predicted targets of these 10 miRNAs. (**G** and **H**) The protein levels of Hp1bp3, Gnal, Rem2, Gria1, and Gria2 in the mPFC homogenates from control and OE-Hp1bp3 mice were examined by Western blotting. Representative images are shown in (G), and the quantitative analysis is shown in (H) (*P < 0.05, **P < 0.01, ***P < 0.001, *n* = 4). GAPDH, glyceraldehyde phosphate dehydrogenase. (**I**) The protein levels of Gnal, Rem2, Gria1, and Gria2 in the mPFC homogenates from Impaired-ext and Normal-ext mice were examined by Western blotting. The representative images are shown in right and the quantitative analysis is shown in left (*P < 0.05, **P < 0.01, *n* = 4). (**J**) The protein levels of Gnal, Rem2, Gria1, and Gria2 in the mPFC homogenates from Cpeb1 WT+Scr, Cpeb1 cKO+Scr, Cpeb1 WT+Sh-Hp1bp3, and Cpeb1 cKO+Sh-Hp1bp3 mice were examined by Western blotting. The representative images are shown in right and the quantitative analysis is shown in left. (*P < 0.05, **P < 0.01, ***P < 0.001, *n* = 4).

We then queried how neuronal Hp1bp3 translation activation could induce synaptic disorder. It is known that Hp1bp3 is a histone H1–like chromatin protein that can promote global miRNA biogenesis (*31*). We then performed a miRNA array in the neuronal *Hp1bp3* overexpression mPFC tissues. We found that overexpressing of *Hp1bp3* indeed induced the up-regulation of numerous miRNAs (Fig. 6, A and B). We further validated these deregulated miRNAs in the Impaired-ext mice and found that 10 of them (let-7a-5p, miR-122-5p, miR-130b-5p, miR-146a-5p, miR-28a-5p, miR-30a-5p, miR-335-5p, miR-342-3p, miR-433-5p, and miR-7a-5p) were increased (Fig. 6C). The up-regulation of these 10 miRNAs in neuron-specific *Cpeb1* knockout mice could be fully restored when neuron-specific shRNA targeting *Hp1bp3* was administered (fig. S10A). We then chose these 10 miRNAs for further study. Quantitative PCR (qPCR) analysis suggested that the primary transcripts (pri-) of these 10 miRNAs were not up-regulated, while all the precursors (pre-) were increased upon *Hp1bp3* overexpression, which further verified that the boost of miRNA biogenesis by Hp1bp3 was due to promoting the processing rather than transcription of pri-miRNAs (Fig. 6, D and E). We then used TargetScan to analyze and identify target genes for the 10 selected miRNAs and found that most of the targets are enriched in the “Synapse organization” and “Dendrite development” pathways (fig. S10B). Considering the negative regulatory nature of miRNAs on their target genes (*32*), we integrated these miRNA target genes with the down-regulated proteins identified in our proteomic dataset (fold change < −1.2 and *P* < 0.05) (table S1). Cytoscape for further analysis (*33*) of integrated data was used, and we noticed that these 10 miRNAs might centrally target Gnal, Rem2, Gria1 and Gria2 (Fig. 6F). Western blots and immunofluorescence data suggested that Gnal, Rem2, Gria1 and Gria2 were decreased in the IL neurons upon the neuron-specific *Hp1bp3* overexpression, neuron-specific *Cpeb1* knockout, and the Impaired-ext mice (Fig. 6, G to J, and fig. S11, A and B). Silencing of *Hp1bp3* in neurons effectively restored the levels of Gnal, Rem2, Gria1, and Gria2 in neuron-specific *Cpeb1* knockout mice (Fig. 6J). Considering the critical role of Gnal, Rem2, Gria1, and Gria2 in mediating the synaptic transmission and structure (*34*–*38*), we therefore proposed that neuronal Hp1bp3 promotes a complex miRNA network to regulate postsynaptic proteins acting as downstream effectors for Cpeb1 in neurons.

### Disturbance of the microglia-specific translational signal causes microglia to exhibit an aged-like phenotype and leads to the accumulation of perisynaptic ECM

**Fig. 7.**
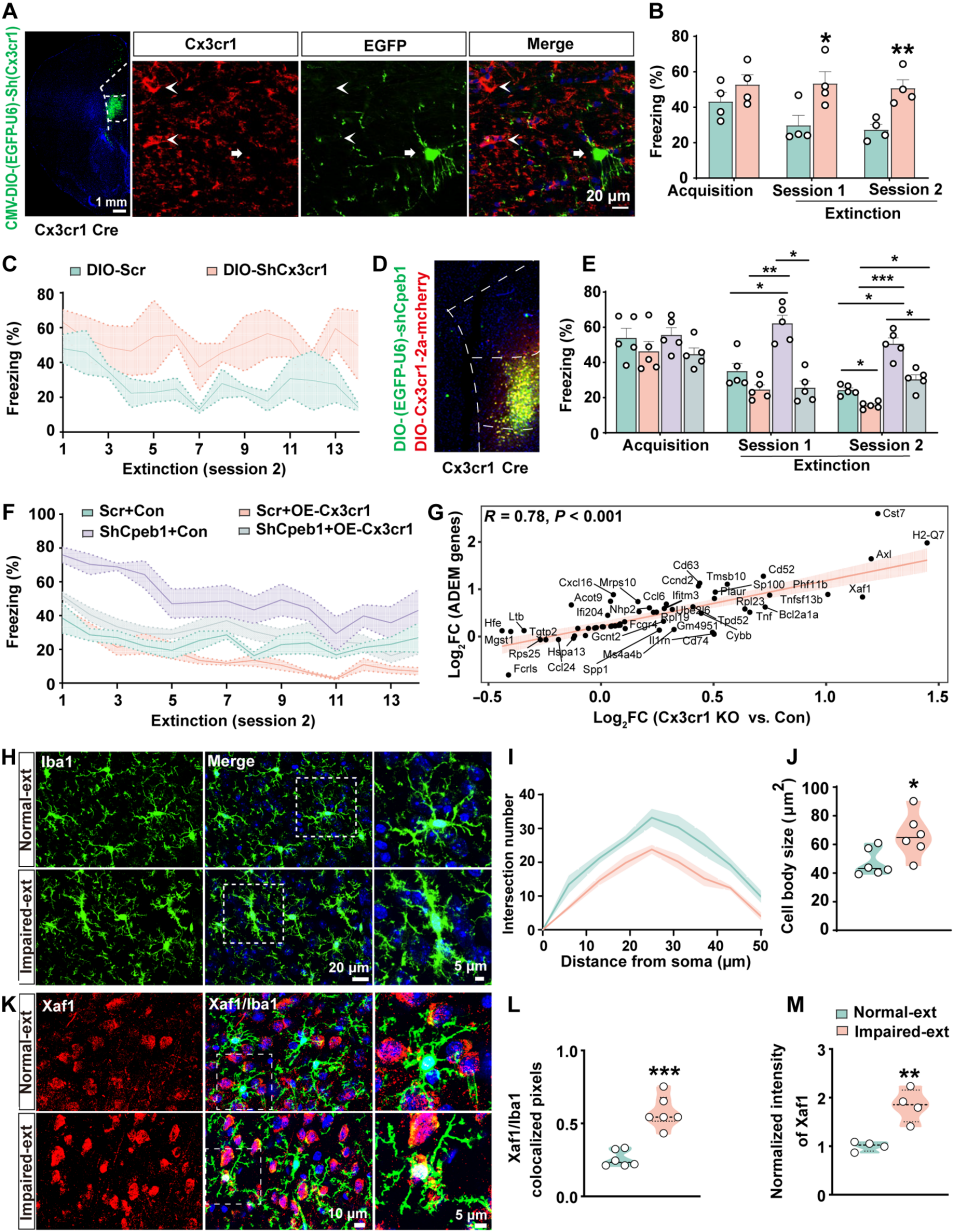
Microglial Cpeb1-Cx3cr1 signaling regulates fear extinction and induces an aged-like microglial phenotype. (**A**) Representative confocal image (right) for virus injection in the *IL* of *Cx3cr1*-*Cre* mice. Coronal slices with infected (EGFP) microglia were stained with anti-Cx3cr1 (red). Arrowheads indicate virus-infected microglia, and arrows show noninfected microglia. (**B**) Average freezing percentage during fear memory acquisition and the first and second sessions of fear extinction in DIO-Scr and DIO-ShCx3cr1 groups (*P < 0.05, **P < 0.01, *n* = 4 per group). (**C**) Freezing percentages across 14 trials in the second extinction session of DIO-Scr and DIO-ShCx3cr1 groups (*n* = 4 per group). (**D**) Representative confocal image for virus injection into the IL from *Cx3cr1*-*Cre* mice. (**E**) Average freezing percentage during fear memory acquisition and the first and second sessions of fear extinction learning in Scr+Con, Scr+OE-Cx3cr1, Sh-Cpeb1+Con, and Sh-Cpeb1+OE-Cx3cr1 groups (*P < 0.05, **P < 0.01, ***P < 0.001, *n* = 5 to 7 per group). (**F**) Freezing percentages across 14 trials in the second extinction session for Scr+Con, Scr+OE-Cx3cr1, Sh-Cpeb1+Con, and Sh-Cpeb1+OE-Cx3cr1 groups (*n* = 5 to 7 per group). (**G**) Scatter plot showing linear regression of log_2_FC of overlapping genes between ADEM gene sets and *Cx3cr1*-deficient microglia gene sets, with a positive correlation. Pink shading shows 95% confidence interval, and Pearson’s correlation coefficient and *P* value are shown. (**H** to **J**) Microglia from the Impaired-ext group display reduced branching and enlarged cell bodies. Confocal images are shown in (H), and Sholl analysis (I) and microglial cell size (J) were quantified by ImageJ (*P < 0.05, *n* = 6 cells from four mice per group). (**K** to **M**) Immunofluorescence on *IL* sections from Impaired-ext and Normal-ext groups using anti-Xaf1 (Red) and anti-Iba1 (Green) antibodies. Representative confocal images are shown in (K). Xaf1/Iba1 colocalized pixels (L) and normalized intensity of Xaf1 (M) were calculated by ImageJ (**P < 0.01, ***P < 0.001, *n* = 6 cells from four mice per group).

To investigate whether the microglial Cpeb1-Cx3cr1 signal was involved in fear extinction, we administered AAV-DIO-(EGFP-U6)-ShCx3cr1 virus into the IL of the *Cx3cr1*-*Cre* mice (fig. S12, A and B) to specifically knock down *Cx3cr1* in microglia (DIO-sh-Cx3cr1), which was validated by the immunofluorescence and Western blot data (Fig. 7A and fig. S12, C and D). In addition, Nissl staining showed that knockout of *Cx3cr1* did not affect cell density (fig. S12, E and F). Behavioral tests showed that microglial *Cx3cr1* knockdown did not affect fear memory acquisition but elevated the freezing percentage during the sessions 1 and 2 of fear extinction (Fig. 7, B and C). To further elucidate the specific role of Cx3cr1 in mediating impaired fear extinction caused by loss of Cpeb1 in microglia, we administered AAV2/6-DIO-CX3CR1-2A-mCherry and AAV2/6-DIO-(EGFP-U6)-ShCpeb1 into the *IL* of *Cx3cr1*-*Cre* mice together to overexpress *Cx3cr1* in the microglia of microglia-specific *Cpeb1* knockout mice (Fig. 7D). Western blotting and behavioral assays showed that OE-Cx3cr1 not only attenuated the down-regulation of Cx3cr1 (fig. S12, G and H) but also rescued the impaired fear extinction caused by microglial Cpeb1 loss (Fig. 7, E and F). These data indicated that microglial Cpeb1 loss hindered fear extinction learning processes by suppressing the levels of microglial Cx3cr1.

We then aimed to interrogate the underlying mechanisms through which microglial *Cx3cr1* translation suppression affected fear extinction learning. Considering that *Cx3cr1*-deficient microglia could affect microglial homeostasis, we then analyzed transcriptome characteristics of isolated brain microglia from 2-month *Cx3cr1^+/+^*, *Cx3cr1*^*+/*−^ and *Cx3cr1*^−*/*−^ mice (*39*). We found that the fold changes of overlapping genes in the *Cx3cr1*-deficient microglia were positively correlated with the age-dependent microglia (ADEM) genes (*40*) (Fig. 7G) rather than well-known senescence genes (*41*–*45*) or senescence-associated secretory phenotype (SASP) genes (*46*, *47*), which indicates an aged-like but nonsenescent phenotype (fig. S13, A and B). Meanwhile, microglia cells in the Impaired-ext group exhibited less branching morphology and larger cell bodies, indicating an aged-like state (Fig. 7, H to J). The aged-like state of microglia in Impaired-ext group was further confirmed by the immunostaining of Xaf1 and Tnfsf13b, which were significantly up-regulated ADEM genes in *Cx3cr1*-deficient microglia (Fig. 7, K to M, and fig. S13, C and D). However, the level of β-galactosidase (β-gal), a marker of cell senescence, did not change in microglia in Impaired-ext mice compared to Normal-ext mice, indicating that the microglial cells in the Impaired-ext group exhibited an aged-like but nonsenescent phenotype (fig. S13, E and F), consistent with studies showing that *Cx3cr1*-deficient microglia display premature aging (*39*).

**Fig. 8.**
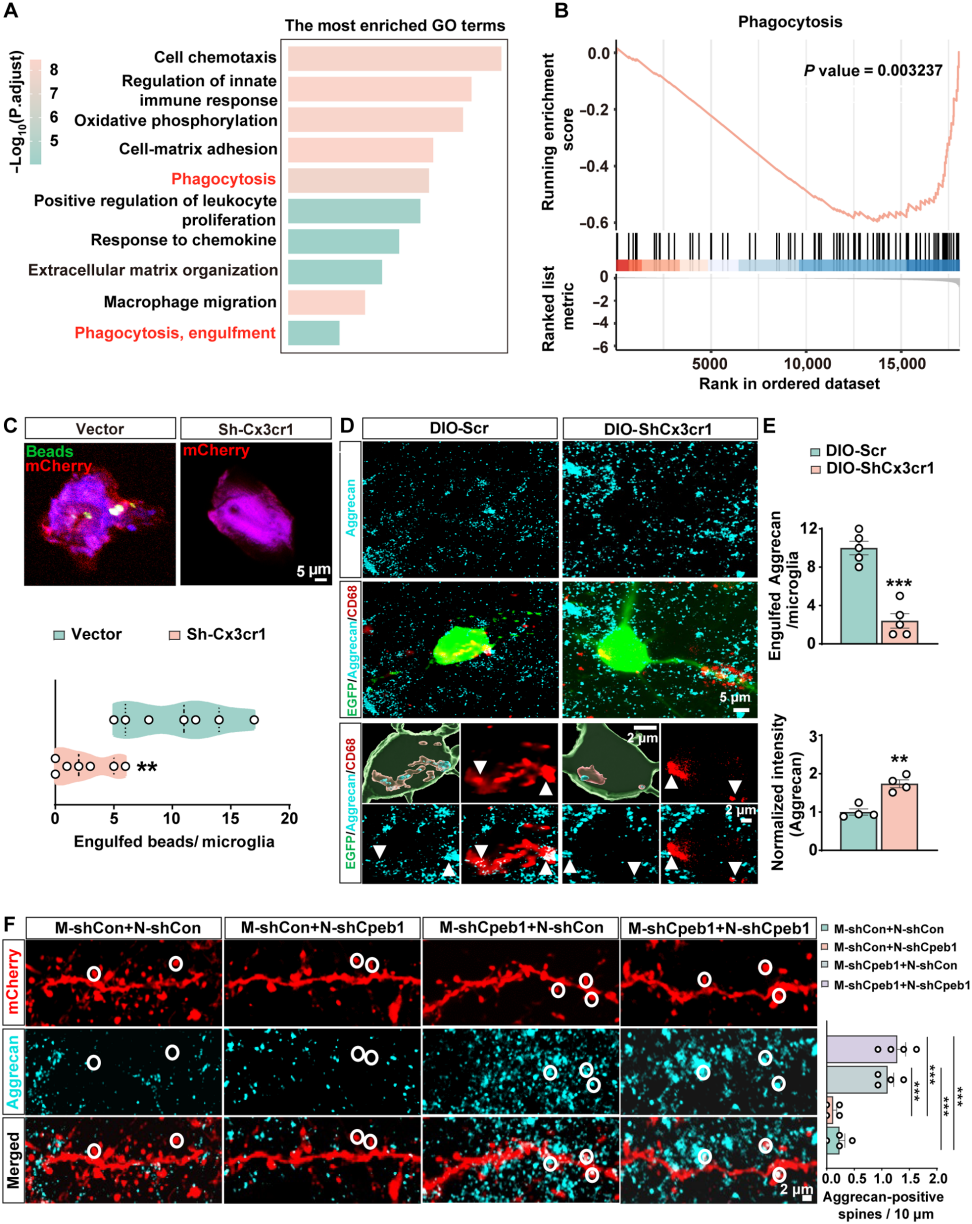
Disturbance of microglial Cpeb1-Cx3cr1 signaling leads to impaired phagocytic ability in aged microglia and the accumulation of perisynaptic ECM. (**A**) Functional enrichment analysis of differentially expressed genes between *Cx3cr1*-deficient microglia and WT microglia (fold change >1.2, *P* < 0.05). (**B**) GSEA of phagocytosis-related genes in *Cx3cr1*-deficient microglia compared with WT microglia. (**C**) BV2 cells with knocked-down *Cx3cr1* showed reduced phagocytic ability of fluorescent latex beads. Representative confocal images are shown in the top panel of (C), and engulfed beads per microglia were calculated in the bottom panel of (C) (**P < 0.01, *n* = 7 microglia cells from two replicates per group). (**D** and **E**) Aggrecan protein within microglial lysosomes from DIO-Scr and DIO-ShCx3cr1 group. Coronal slices from virus infected (EGFP) microglia cells were stained with anti-Aggrecan (cyan) and CD68 (red) antibody. The representative confocal images and 3D reconstruction are shown in (D). The number of engulfed Aggrecan in microglia and the normalized intensity of Aggrecan were calculated in (E) (**P < 0.01, ***P < 0.001, *n* = 5 microglia cells from four mice per group). (**F**) Aggrecan colocalization with dendritic spines in the *IL* labeled with AAV9-hSyn-mCherry and AAV9-hSyn-mCherry-shcpeb1 virus from M-shCon+N-shCon, M-shCon+N-shCpeb1, M-shCpeb1+N-shCon and M-shCpeb1+N-shCpeb1 mice. Representative confocal images of Aggrecan colocalization with dendritic spine are showed in the left of (F), and the Aggrecan positive dendritic spine was calculated in the right of (F) (***P < 0.001, *n* = 4 neurons from three mice per group).

To gain insight into the biological characteristics of aged microglia with *Cx3cr1* deficiency in nonaged brain, we performed pathway enrichment analysis of differentially expressed genes between WT and *Cx3cr1*-deficient microglia. Gene Ontology (GO) analysis showed that differential genes were mainly related to cell chemotaxis, immune response, phagocytosis pathways, and ECM organization (Fig. 8A). In addition, gene set enrichment analyses (GSEAs) showed a substantial reduction in a set of phagocytosis genes (Fig. 8B). Our phagocytosis assay further showed that knocking down *Cx3cr1* in primary microglia cells inhibited microglial phagocytosis of latex beads (Fig. 8C). These findings are consistent with the role of aged microglia in dampened phagocytosis (*48*) and Cx3cr1’s role in promoting microglial phagocytosis (*49*).

We next aimed to investigate the underlying mechanisms by which *Cx3cr1* translation suppression influenced synaptic function. Among the enrichment pathways between WT and *Cx3cr1*-deficient microglia, ECM-related pathways have been shown played an important role in synaptic and spine plasticity (*50*) (Fig. 8A). Aggrecan, a core component of the ECM structures in perineuronal nets, impaired the plasticity of the visual cortex in mice (*51*). Deposition of Aggrecan near synapses resulted in a decrease in the number of spines (*52*). Aggrecan could be degraded by the action of a bacterial enzyme, chondroitinase ABC (ChABC) (*53*), which could improve morphological changes in dendritic spines in the visual cortex of adult mice (*54*) and promote the erasure of fear memories (*55*). These data suggest a possible role of ECM remodeling in contributing to the impaired fear extinction. Given that aged microglia’s ability to engulf was diminished, we investigated whether their capacity to engulf Aggrecan was also affected. Using super-resolution imaging and three-dimensional (3D) reconstruction, we found that microglia-derived *Cx3cr1* reduced the amount of engulfed Aggrecan, while the relative fluorescence intensity of Aggrecan was increased compared to the control group (Fig. 8, D and E). We found that compared to the control group, the inhibition of *Cpeb1* in microglia significantly increased the number of spines fully contacted by Aggrecan, while the inhibition of *Cpeb1* in neurons did not affect the number of spines in contact with Aggrecan (Fig. 8F). These findings indicate that the *Cx3cr1*-translated suppressive microglia exhibit an aged phenotype, impairing their capability to engulf the ECM Aggrecan, resulting in the increase in the number of Aggrecan-positive spines. In summary, these data suggest that the disturbance of microglial Cpeb1-Cx3cr1 translational signal promotes ECM remodeling and ECM deposition near synapses, hindering spine formation.

## DISCUSSION

**Fig. 9.**
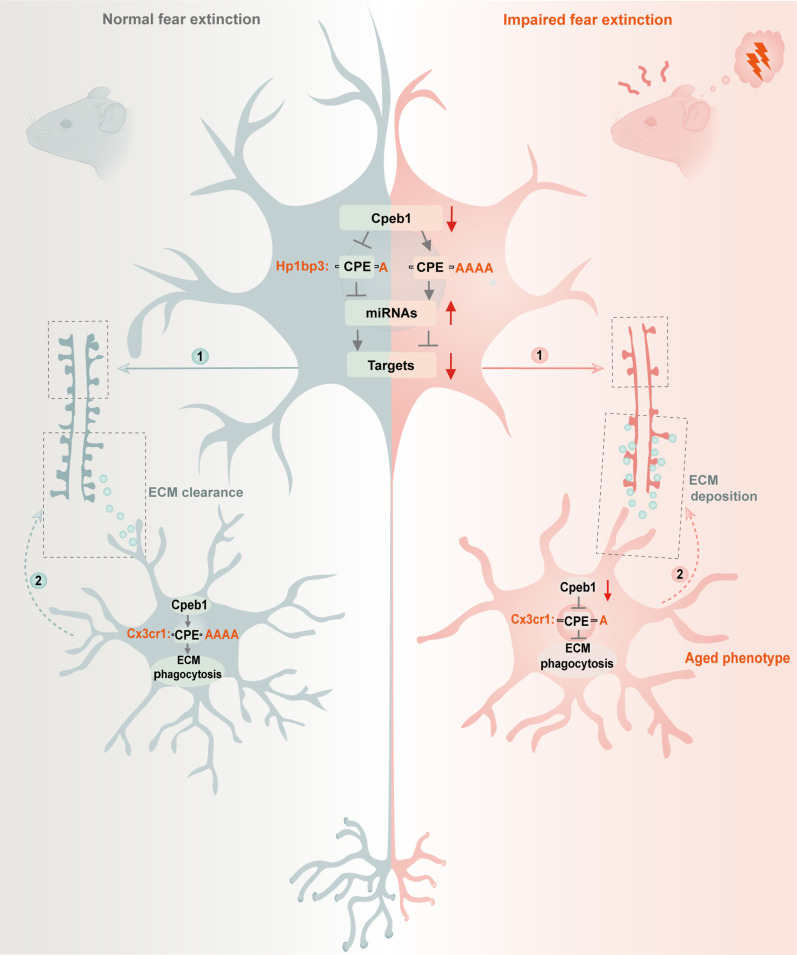
A working model illustrating Cpeb1 bidirectionally drives neuron- and microglia-specific translational programs to promote fear extinction. Our findings demonstrate that Cpeb1 in the *IL* regulates fear extinction by controlling *Hp1bp3* translation activation in neurons and *Cx3cr1* translation suppression in microglia. Neuronal *Hp1bp3* translation activation increases a network of miRNAs affecting postsynaptic proteins, hindering spine formation and plasticity. Microglial *Cx3cr1* translation suppression results in an aged phenotype impairing their ability to engulf ECM Aggrecan, promoting perisynaptic ECM deposition, and reducing dendritic spine density. Together, Cpeb1 bidirectionally drives neuron- and microglia-specific translational program to promote fear extinction.

In this study, we first verified the critical role of protein synthesis in fear extinction by using a Tet-On 3G–based genetic approach. Subsequently, we demonstrated that cell type–specific translational programs regulated by the decay of Cpeb1 in neurons and microglia of the IL played an important role in mediating impaired fear extinction. Fantastically, the translation activation of *Hp1bp3* in neurons and the translation suppression of *Cx3cr1* in microglia acted as key translational downstream events of Cpeb1 loss. Specifically, elevation of neuronal Hp1bp3 stimulates a miRNA network that affects postsynaptic proteins, hindering spine formation and plasticity, while suppression of microglial Cx3cr1 induces the aged-like microglia with impaired ability to engulf ECM Aggrecan, promoting perisynaptic ECM deposition and reducing dendritic spine density (Fig. 9).

Evidence suggests that the prelimbic cortex (*PL*) and anterior cingulate cortex (*ACC*) in the mPFC mediates fear memory expression, while the infralimbic cortex (*IL*) plays a role in fear extinction (*56*–*58*). Research has demonstrated that inhibiting protein synthesis in the prelimbic cortex (*PL*) disrupts contextual fear memory, while similar inhibition in the *ACC* does not affect contextual fear memory (*59*). In addition, previous studies have highlighted that the synthesis of specific proteins within the *PL*, such as *N*-methyl-d-aspartate receptors (NMDARs) (*60*), the plasticity-associated protein kinase M-ζ (PKM-ζ) (*61*), and the motor protein kinesin family member 3B (KIF3B) (*62*), is essential for fear memory formation. Furthermore, protein synthesis inhibition in the lateral ventricles or insular cortex does not impair the consolidation or extinction of cued fear memories (*16*). However, inhibition of protein synthesis specifically in the *IL* disrupts the extinction of cued fear memory without affecting its consolidation (*16*). This underscores the critical role of protein synthesis in the *IL* for the successful extinction of cued fear memory. The predominant approach involved the use of protein synthesis inhibitors, such as anisomycin or rapamycin, administered via subcutaneous injections (*63*, *64*) or delivered via stereotaxic injection into either the lateral ventricle or specific brain regions (*18*, *65*, *66*). Anisomycin, which acts by blocking peptidyl transferase activity during translation elongation, has been shown to result in nearly 30% inhibition of brain protein synthesis by stereotaxic injection (*67*). Subcutaneous anisomycin achieves an inhibition rate of more than 90% on brain protein synthesis in the first 2 hours and more than 60% in the following 2 hours (*64*, *68*). Rapamycin inhibits the mechanistic target of rapamycin complex 1 (mTORC1) to affect translational initiation and elongation, resulting in a 50% decrease in protein synthesis in lymphocytes and peripheral skeletal tissue (*69*, *70*). However, the administration of common drugs via microinjection can induce a range of adverse effects. Anisomycin, for instance, has been associated with heightened activation of stress signaling pathways (*71*), induction of gene superinduction (*72*), and facilitation of lysosomal degradation (*73*). Similarly, rapamycin perturbs numerous intricate intracellular signaling cascades implicated in cellular growth, proliferation, autophagy, and metabolic regulation (*74*). Furthermore, subcutaneous injection of anisomycin, despite high protein synthesis inhibition rates, fails to inhibit protein translation in certain brain regions and increases adverse reactions, such as skin allergies. In addition, conventional protein synthesis inhibitors lack temporal control over protein synthesis and the ability to selectivity target specific molecularly diverse cell populations, making it impossible to accurately regulate protein synthesis within distinct cellular subsets. Given the limitations of current approaches for protein translation inhibition in memory extinction research, it is urgent to propose a more effective and temporally controllable method for achieving protein translation inhibition in vivo. Ribosome inactivating protein (RIP) (*75*), that depurinates A4324 on the sarcin-ricin loop of the 28S rRNA and blocks translation elongation, forms the basis of gePSI and achieves almost complete inhibition of neuronal protein synthesis in vitro (*20*). However, its effects in vivo are not clear. Here, we used the Tet-on system, in which the introduction of the Dox induces the expression of gePSI in mice. Our findings showed that this approach achieved nearly 60% general protein inhibition in the brain of live animals and hindered fear extinction, while ceasing the administration of Dox reversed fear extinction inflexibility by restoring protein synthesis. Moreover, gePSI expression in a single neuron in vitro impairs the structural plasticity of neuronal synapses (*20*), which is consistent with our results on the disruption of neuronal synapse structure and plasticity caused by translational disturbances in neurons. This further proves the feasibility of our approach for protein translation inhibition. Together, our approach enables the rapid, controllable, and reversible achievement of cell type–specific temporal control of protein synthesis in a specific brain region, without affecting cellular viability. This approach holds significant importance for future investigations into the impact of protein synthesis inhibition on learning and memory in various cell populations of animals.

The cellular composition of the mPFC is notably complex, comprising a diverse array of neuronal subtypes, glial cells, and intricate synaptic networks (*76*). This complexity underscores the pivotal roles of diverse cell types in encoding and regulating many different cognitive behaviors (*77*). For instance, in combination with single-cell RNA sequencing, microendoscopic Ca^2+^ imaging, and photoinhibition approaches, the cell type–specific neural circuits in the mPFC had been identified in the goal-directed behavior (*77*), flexible behavior (*78*), social interaction (*79*), and choice and reward context encoding (*80*). Optogenetic modulation of specific neuron subtypes (*81*) or cell type–specific modulation of NMDARs (*82*) in the mPFC has been implicated in rapid antidepressant responses. Moreover, cocaine administration can induce specific transcriptional adaptations in multiple neuron subtypes, with these changes becoming particularly pronounced during extended periods of drug withdrawal (*83*). In addition, a cell type–specific transcriptomic analysis suggested the oligodendrocytes and inhibitory neurons are most affected in alcohol dependence in mPFC (*84*). Therefore, investigating the cell type–specific responses of the mPFC in fear extinction, which rely on a complex pattern of brain region and cell type–specific processes (*85*), will benefit the comprehensive understanding of its underlying mechanisms and offer promising avenues for therapeutic interventions targeting extinction disability disorders.

CPEB isoforms play distinct roles in regulating synaptic plasticity, memory formation, and extinction. Cpeb1, the most studied isoform, is essential for extinction but not for memory consolidation, as *Cpeb1*-*gKO* mice exhibit intact fear memory consolidation but impaired extinction of contextual fear memories (*24*). Cpeb2 is critical for hippocampus-dependent memory; *Cpeb2*-*cKO* mice display deficits in contextual fear memory and spatial memory consolidation in the Morris water maze (MWM) tests (*86*). Cpeb3 appears to enhance memory consolidation, with *Cpeb3-gKO* mice showing improved spatial memory and enhanced consolidation of short-term fear memory (*86*). Cpeb4, however, does not seem to affect general learning and memory, as *Cpeb4-gKO* mice exhibit normal performance in MWM and contextual fear conditioning, along with unaltered synaptic transmission and spine morphology (*87*). Despite these findings, it remains unclear how different CPEB isoforms in specific cell types contribute to memory processes. In our study, we observed that reducing Cpeb1 in neurons and microglia within the *IL* region impairs extinction of cued fear memory without affecting its consolidation, indicating cell type–specific role of Cpeb1 in extinction pathways. Moreover, cell type–specific loss of Cpeb1 initiates cell type–specific translation programs, consequently playing a crucial role in fear extinction. Although the absence of Cpeb1 in both neurons and microglia is implicated in fear extinction, the magnitude of their respective contributions varies. Compared to the control group, the absence of Cpeb1 in neurons causes mice to display increased freezing during the initial phase of extinction learning, while its absence in microglia leads to higher freezing levels during the second phase of extinction learning rather than the first. In addition, the absence of Cpeb1 in neurons leads to mice exhibiting a higher freezing tendency compared to the absence of Cpeb1 in microglia during the second phase of extinction. These findings indicate that the absence of Cpeb1 in neurons may have a more pronounced and direct effect on fear extinction inflexibility.

In our study, we provided previously unknown insights into the involvement of Cpeb1-mediated protein translation events across distinct cellular populations, further affirming the importance of Cpeb1 in fear extinction. It is known that Cpeb1 is a critical regulator of RNA translation and orchestrates various important physiological functions by modulating cytoplasmic polyadenylation, a crucial process for regulating the translation of specific mRNAs (*25*). The precise mechanism by which Cpeb1 modulates translation and its specific impact on fear extinction across various cell types remains elusive and warrants further investigation. By integrating data from proteomics, RNA sequencing, and RNA immunoprecipitation sequencing, our study reveals that the absence of Cpeb1 triggers distinct translation events in neurons and microglia, respectively, thereby impairing fear extinction. Specifically, Cpeb1 binds with the unique CPEs and affects the poly(A) tail elongation of *Hp1bp3* and *Cx3cr1*, leading to the translation suppression of *Hp1bp3* in neurons and the translation activation of *Cx3cr1* in microglia. In the Impaired-ext mice, neuronal Hp1bp3 is elevated while microglial Cx3cr1 is reduced, as seen in the *Cpeb1* knockout mice. Silencing of neuronal *Hp1bp3* or supplementing microglial *Cx3cr1* effectively rescues fear extinction inflexibility in *Cpeb1* conditional knockout mice. It may seem somewhat paradoxical that Cpeb1 regulates the translation of *Hp1bp3* and *Cx3cr1* in neurons and microglia in opposite directions. However, because Cpeb1’s activation or inhibition of target gene translation is dependent on the number and relative positioning of CPEs within the 3′UTR, it is reasonable for Cpeb1 to exert a bidirectional regulatory effect on *Hp1bp3* and *Cx3cr1* in different cell types (*26*).

Here, we also delve into the contribution of cell type–specific translational programs to fear extinction, uncovering that both neuronal Hp1bp3 and microglial Cx3cr1 are implicated in regulating synaptic function through direct and indirect pathways. Hp1bp3 is a multifunctional protein involved in various biological processes, including gene expression regulation, cell cycle progression, cell development, and miRNA biogenesis. By interacting with heterochromatin protein HP1, Hp1bp3 influences chromatin compaction and modulates gene silencing (*88*). Hp1bp3 contributes to cell cycle progression by maintaining heterochromatin integrity during G_1_-S phase transition, thus regulating the duration of G_1_ phase (*89*). In addition, Hp1bp3 binds to nucleosomes and protects linker DNA from degradation by micrococcal nuclease, which also has been identified as a mediator of cell viability and growth (*90*). Recently, Hp1bp3 has also been found to specifically associate with the microprocessor, promoting cotranscriptional miRNA processing (*31*) and adding a previously unknown dimension to its regulatory role in gene expression. In the current study, we characterized the miRNA transcriptome and found that Hp1bp3 enhanced miRNA biogenesis by facilitating pri-miRNA processing rather than transcription, suggesting a widespread and conserved mechanism for Hp1bp3 in eukaryotes. Furthermore, we have identified the specific downstream molecules (Rem2, Gnal, Gria1, and Gria2) that acted as the common targets by the network of miRNAs. Rem2 is a member of the small Ras-like guanosine triphosphatase family, identified as an important mediator of synaptic development (*91*). Knocking out *Rem2* in hippocampal neurons reduces dendritic spine density and maturation (*35*). The Gα (olf) subunit of the Gnal-encoded guanine nucleotide–binding protein plays a key role in dopamine signaling in striatal medium spiny neurons (*92*). Studies have found that the complexity of dendrites in striatal neurons of heterozygous *Gnal* knockout mice is significantly reduced (*34*). Gria1 and Gria2 are two subunits of the AMPA receptor, which play a crucial role in synaptic transmission and plasticity (*38*). It has been reported that interfering with AMPAR by peptides to inhibit the expression of GluA1 and GluA2 in optic tectal neurons of the *Xenopus* retinotectal system results in decreased dendritic branching and complexity (*37*). Therefore, the down-regulation of these proteins in neurons is critical for the synaptic transmission and structure.

In various settings, microglial cells, as resident brain macrophages, exhibit alterations in morphology, phagocytic capacity, and transcriptional profile to promptly detect neuronal changes and maintain brain homeostasis (*93*). Cx3cr1 is a chemokine receptor expressed on microglial cells in the brain, and disruption of Cx3cr1 is accompanied by dysfunction of microglial status and function (*39*). In our study, the transcriptomes of *Cx3cr1*-deficient microglia and fluorescence staining unveiled the aged-like microglia rather than a senescent phenotype in impaired fear extinction. Aged and senescent microglia are two distinct states of microglial cells that occur with advancing age. Aging microglia refer to those that have naturally aged over time (*94*), exhibiting characteristics such as oxidative stress, DNA damage, telomere shortening, reduced phagocytic function, lipid metabolism disorders, and altered morphology (including fewer dendritic branches and larger cell bodies) (*94*–*97*). Senescence represents a more severe and irreversible stage of aging (*98*), marked by irreversible growth arrest and development of a SASP (*99*). Aged microglia and senescent microglia exhibit different characteristics under various stimuli or conditions. For example, microglia isolated from newborn mice show an aged phenotype in prolonged culture, featuring reduced phagocytic ability, motility, and autophagy, along with increased SA-β-gal activity (*100*). Similarly, aged microglia isolated from aging transgenic mice via flow cytometry exhibit lipofuscin deposits, reduced process complexity, increased granularity, and enhanced expression of proinflammatory and anti-inflammatory cytokines (*97*). Senescent microglia induced by chronic tau exposure exhibit cell cycle arrest, DNA damage, lamin-B1 loss, impaired tau clearance, and formation of a SASP (*99*). Iron-overloaded senescent microglia display decreased branching, elevated iron storage and ferritin expression, increased release of neurotoxic substances, and reduced capacity for phagocytosis of debris and toxic protein aggregates (*101*). Senescent microglia induced by corticosteroid hormones show increased SA-β-gal activity, increased expression of tumor suppressor genes, and impaired phagocytic function (*102*). In addition, transcriptomic differences are also observed in aged and senescence microglia. Senescent microglia induced by replicative stress are enriched in DAM signatures, accompanied by increased of SA-β-gal activity, telomere shortening, neuritis, and synaptic damage (*42*). Aged-like microglia induced by three rounds of depletion–repopulation stimulation (3×DR) is enriched in ADAM landscapes, along with reduced phagocytic capacity, shortened telomeres, and hindered myelin development (*40*). A subset of microglia rich in ADAM genes is found in *Cx3cr1*-deficient mice with extinction learning deficits, representing an age-like phenotype induced by fear extinction disorder. Moreover, GSEAs showed a substantial reduction in a set of phagocytosis genes in *Cx3cr1*-deficient microglia compared to WT microglia, suggesting a potential decline in phagocytic function in aged-like microglia influenced by fear extinction disorder.

How does Cpeb1’s downstream effector Cx3cr1 in microglia impair fear extinction? Loss of Cx3cr1 in microglia was shown to result in the disruptions in the communications of neurons and microglia and influence synaptic pruning (*30*). Previous researches have shown that activated microglia inhibit gamma-aminobutyric acid (GABA) neuronal activity by engulfing their dendritic spines, ultimately leading to the extinction of anxiety-like behaviors induced by restraint stress (*103*). Developing juvenile microglia exhibit an immature state reminiscent that contributed to extinction learning deficits by elevated spine pruning and reduced spine formation (*104*). Our results would be of much interest to raise that the aged-like microglia in nonaged brain disrupt synapse structural plasticity by preventing local clearance of the ECM around synapses, leading to the impairments in fear extinction. Our findings align with previous reports revealing that the ablation of *Cx3cr1* in mice resulted in the deposition of the ECM in the hippocampal dentate gyrus (*DG*) region (*105*), the deposition of ECM near synapses resulted in a decrease in the number of spines (*52*), and degradation of ECM by the ChABC promoted the extinction of fear memories (*55*).

Although our findings suggest that Cpeb1-dependent translational events mediating fear extinction is predominantly restricted to the *IL* cortex, the contribution of Cpeb1 and its downstream signaling pathways to fear extinction is likely not confined to this region alone. For instance, Cpeb1 has been implicated in hippocampal-dependent memory and cortical plasticity, suggesting that the processes identified here could extend to memory systems beyond the mPFC (*106*). Overexpression of *Cpeb1* has been shown to rescue deficits in long-term potentiation, a synaptic mechanism fundamental to learning and memory across various brain regions, including the hippocampus (*107*). This indicates that Cpeb1 plays a crucial role in synaptic plasticity and memory formation, and its influence may generalize to other regions beyond the mPFC. *Hp1bp3* knockdown in the hippocampus results in impaired working memory, linked to reduced synaptic plasticity in hippocampal neurons (*108*). Hp1bp3’s epigenetic functions, particularly in chromatin remodeling, suggest that it could affect synaptic plasticity and memory formation not only in the mPFC but also in regions like the hippocampus and neocortex (*109*). Meanwhile, Cx3cr1 signaling is critical for maintaining synaptic structure and plasticity across brain regions. Deletion of *Cx3cr1* in mice has been shown to disrupt dendritic spine pruning, synapse maturation, and connectivity between the hippocampus and mPFC (*110*). These mice also exhibit cognitive deficits, including impaired social interaction and repetitive behaviors and hippocampus-dependent spatial memory deficits (*29*), highlighting Cx3cr1’s broader role in cognitive function. Our studies further indicate that Hp1bp3 overexpression in the *IL* cortex impairs AMPAR-mediated synaptic transmission, dendritic complexity, and spine morphology. Similarly, loss of *Cx3cr1* in microglia disrupts synaptic structural plasticity by preventing local clearance of the ECM around synapses. These changes are both indicative of impaired synaptic plasticity, which is essential for adaptive learning and behavioral flexibility. Thus, the Cpeb1-mediated dysregulation of Hp1bp3 and Cx3cr1 likely contributes to deficits not only in fear extinction but also in broader cognitive functions, such as hippocampus-dependent working memory and behavioral flexibility.

In summary, we identified cell type–specific translational programs in the IL that are essential for fear extinction, underscoring the importance of thoroughly investigating the molecular mechanisms of cell type–specific translation. These findings have significant implications for the development of previously unidentified tools and approaches aimed at addressing PTSD and potentially other disorders characterized by deficits in extinction.

## MATERIALS AND METHODS

### Animals

Adult male C57BL/6 J mice were purchased from the National Resource Center of Model mice in Nanjing, China. Genotyping for *Cx3cr1*-*Cre* mice (obtained from Shanghai Model Organisms, China) was performed via multiplex PCR using the primer pair 5′-ACACCAGAGACGGAAATCCATCG-3′ and 5′-CAACGAGTGATGAGGTTCGCAAG-3′. *Cpeb1^flox/flox^* mice were generated from GemPharmatech Co., Ltd. in Jiangsu, China. Genotyping for *Cpeb1^flox/flox^* mice was conducted using PCR with primer sequences 5′-GAGCTCACTGTTTCTTGGTGTCCCT-3′ and 5′-CACAGTTGTTACCAGTCACAAGTGG-3′. All mice were housed in a controlled environment, maintaining a 12-hour light/dark cycle, with a stable temperature (22° ± 2°C) and humidity ranging from 40 to 60%. They were provided with ad libitum access to food and water. Ethical approval for all experimental procedures was obtained from the Institutional Animal Care and Use Committee of Huazhong University of Science and Technology (approval no. 2020-S2317). The mice, along with their littermates, were bred at the Experimental Animal Central of Tongji Medical College, Huazhong University of Science and Technology.

### Microarray-based mRNA and miRNA expression profiling

Microarray-based mRNA and miRNA expression profiling were conducted following standardized procedures. Tissues were homogenized using a TissueRuptor homogenizer (Qiagen, Palo Alto, CA, USA) operating at 50/60 Hz, in accordance with the manufacturer’s instructions. Total RNA extraction was performed, and samples were treated with ribonuclease (RNase)–free deoxyribonuclease (Qiagen, Palo Alto, CA, USA). RNA concentration was determined using a spectrophotometer (DU-640, Beckman, USA), and RNA integrity was assessed via 1% agarose gel electrophoresis. miRNA expression analysis was performed using the Mouse OneArray v2 (RmiOA.2) chip, while gene expression analysis used the OneArray microarray v2 (MOA-002) chip. Data normalization and analysis were carried out using the GeneChip Robust Multichip Average method, with normalization conducted using OneArray Software (Phalanx Biotech Group). Only hybridized chips meeting predefined quality control standards were included in the analysis to determine fold changes. Statistical significance was defined as *P* < 0.05.

### Mass spectrometry and data analysis

Proteins were extracted from the mPFC of both the Normal-ext and Impaired-ext groups. The protein lysate underwent heat treatment at 70°C for 1.5 hours to facilitate the release of cross-linked fixed proteins. Subsequently, protein precipitation was achieved by adding precooled acetone, followed by sequential washing steps with precooled acetone, ethanol, and acetone. The resulting protein pellet was resuspended in UA buffer (8 M urea in 0.1 M tris-HCl), and dithiothreitol (final concentration, 2 mM) was added, followed by incubation at 30°C for 1.5 hours. Iodoacetamide (Sigma-Aldrich) was then introduced to the sample, which was incubated in darkness for 60 min. Trypsin was added for overnight digestion, and the reaction was terminated with trifluoroacetic acid (Sigma-Aldrich). Following salt depletion using C18 spin tips (Thermo Fisher Scientific), the sample was loaded onto the Bruker timsTOF Pro mass spectrometer following the manufacturer’s protocol. The assay was performed as previously described (*111*).

Data processing was conducted using PEAKS software. The spectral abundance factor was determined by normalizing the number of protein spectra to the length of the respective proteins. Subsequently, normalization among samples was achieved by dividing the spectral abundance factor by the sum of all spectral abundance factors. Statistical significance was determined with a threshold of *P* < 0.05 and fold change > 1.2. Protein expression hierarchical clustering analysis was performed using Biovinci 1.1.5.

### Cpeb1 RNA immunoprecipitation sequencing and data analysis

The mPFC from C57BL6/J mice was isolated, and the tissue in ice-cold phosphate-buffered saline (PBS) was gently dissociated using a Dounce homogenizer. The cells were collected by centrifuging at 1500 rpm for 5 min at 4°C, and the supernatant was discarded. The cell pellet was resuspended in an equal volume of complete RIP Lysis Buffer (containing RIP lysis buffer, protease inhibitor cocktail, and RNase inhibitor). The lysate was incubated on ice for 5 min, then transferred into nuclease-free microcentrifuge tubes, and stored at −80°C.

For immunoprecipitation bead preparation, 50 μl of magnetic bead suspension was taken and the beads were washed with RIP wash buffer. After washing, the beads were resuspended in 100 μl of RIP wash buffer, and 5 μg of the Cpeb1 antibody (lot 13274-1-AP, Proteintech, China) was added. The mixture at room temperature was rotated for 30 min and briefly centrifuged. The tubes were placed on a magnetic separator, and the supernatant was discarded. After a final wash with RIP wash buffer, the antibody-bound beads were resuspended in RIP immunoprecipitation buffer.

To begin the immunoprecipitation reaction, the RIP lysate was mixed with RIP immunoprecipitation buffer (comprising RIP wash buffer, 0.5 M EDTA, and RNase inhibitor), adjusting the final volume to 1 ml and incubated at 4°C overnight with gentle rotation.

The following day, the supernatant was discarded, and the beads were washed six times with PIP wash buffer, rotating for 5 min at 4°C for each wash. Each immunoprecipitate was resuspended in the proteinase K buffer (containing RIP wash buffer, 10% SDS, and proteinase K) and incubated at 55°C for 30 min with gentle shaking to ensure complete protein digestion. The supernatant was transferred to a fresh microcentrifuge tube, and 250 μl of RIP wash buffer was added, followed by 400 μl of phenol:chloroform:isoamyl alcohol. RNA extraction and purification were performed as previous protocol (*112*).

CPEB1 immunoprecipitation, RNA extraction and cDNA generation were carried out following the protocol of the RIP kit (lot 3778111, Millipore, USA). The cDNA library products ranging from 200 to 500 bp were enriched, quantified, and ultimately sequenced on a NovaSeq 6000 sequencer (Illumina) using the PE150 model.

Raw data analysis was carried out according to the published protocol (*113*). Briefly, STAR software (version 2.5.3a) was used with default parameters. RSeQC (version 2.6) was used for reads distribution analysis. The ExomePeak software (version 3.8) was used for peak calling, with subsequent annotation using BedTools (version 2.25.0). DeepTools (version 2.4.1) facilitated peak distribution analysis. Differentially binding peaks were identified through a Python script using Fisher test. HomerSoftware (version 4.10) was used to determine sequence motifs enriched in peak regions. To mitigate potential false-positive results, criteria were set for fold change of IP/IgG FPKM (fragments per kilobase transcript per million mapping reads) > 2 and *P* < 0.05 for each experiment. Scatter plots of Cpeb1-bound genes were generated using GraphPad 8. GO analysis was conducted using KOBAS software (version: 2.1.1).

### Behavioral assays

#### Fear conditioning and extinction paradigm

On the first day of fear conditioning, animals underwent habituation in the conditioning chamber (context A) for 3 min. Subsequently, they were exposed to an auditory fear conditioning paradigm comprising four pairings of a 30-s tone (CS, 1 kHz, 80 dB) with a 1-s foot shock (US, 0.5 mA), with a 20-s intertrial interval. The onset of the US coincided with the termination of the CS. Following training, animals were returned to their home cages, and the conditioning chambers were sanitized with 70% ethanol.

On the second day, mice were allowed to rest in their cages. Concurrently, Con (On) and gePSI (On) groups administered Dox-containing water (1 mg/ml) for gene expression manipulation.

On the third and fourth days, extinction training and recall testing were conducted in context B. Animals received 14 CS presentations with a 20-s intertrial interval each day. The “No-extinction” group was placed in context B for the same duration without any stimuli. “Acquisition” referred to the average freezing during the initial three trials of extinction training on the third day. “Extinction in session 1 and session 2” denoted the average freezing across all trials during the extinction phase on the third and fourth days, indicative of extinction learning and retrieval, respectively (*19*). Video tracking technology was used to monitor animal activity and freezing behavior, with freezing defined as the absence of movement for 2 s.

Behavioral process for fear conditioning training, Tet-on system, and fear extinction experiment in Fig. 1B was performed as follows:

On day 1, mice underwent habituation (Hab) followed by fear conditioning (Cond). On day 2, gePSI-expressing mice were treated with Dox (gePSI-On) to inhibit protein synthesis, while the control group (gePSI-Off) were treated with vehicle. On days 3 and 4, both groups underwent two extinction sessions (session 1 and session 2). During these sessions, Group gePSI (On) continued receiving Dox, and Group gePSI (Off) continued with the vehicle. On day 5, Group gePSI (On) was split into two subgroups: gePSI (On) and gePSI (On-Off). The gePSI (On) group maintained Dox administration, while the gePSI (On-Off) group switched to the vehicle for 24 hours. On days 7 and 8, mice underwent another two extinction sessions (session 3 and session 4), with Group gePSI (On) continuing Dox and gePSI (On-Off) receiving the vehicle.

#### Elevated plus maze test

The elevated plus maze consisted of two closed arms with 15-cm high transparent walls and two open arms with a slight threshold along the sides. Each mouse was placed in the center of the maze, facing one of the open arms. The animal’s movements were recorded for 10 min using video capture software. The number of visits and the time spent in the open and closed arms were quantified. This experiment was conducted as previously explained (*112*).

#### Open-field test

This behavioral paradigm was performed as previously described (*114*). The open-field test involved placing each mouse in a 40 cm by 40 cm by 30 cm arena for 10 min. Video capture software (WinAVI Video Capture, ZJMedia Digital Technology, China) recorded the mice’s movements. The total distance traveled and the time spent in the center of the arena were measured. Before each trial, the open-field arena was sanitized with 70% ethanol to maintain cleanliness and hygiene.

### Principal components analysis

A comprehensive understanding of the neural mechanisms underlying fear extinction heavily depends on distinguishing individual mice that exhibit different extinction characteristics, particularly those mice that exhibit resilience to the extinction process (Impaired-ext) and those more susceptible to it (Normal-ext). To identify these differences, we analyzed the mean percentage of freezing across the 14 trials in session 2 of extinction learning, as well as the average freezing percentage during the last three trials in a large sample.

Following manual pattern identification, we used PCA (*19*) for dimensionality reduction and clustering of the mice’s original test data. By integrating manual identification with data-driven multivariate analysis, we aim to systematically capture potential behavioral variability within the mouse population.

### Stereotaxic injection

Mice were anesthetized with ketamine (100 mg/kg) and dexmedetomidine (0.5 mg/kg), The head was then secured in the stereotaxic apparatus (RWD Life Science, China). The scalp was sterilized with iodophors, bilateral holes were drilled, and 0.3 μl of the virus was microinfused into the *IL* using an automatic microinjection system (Reno, NV, USA). The AAV2/8-SYN-Cre-P2A-EGFP, AAV2/8-SYN-P2A-EGFP, AAV2/9-SYN-Hp1bp3-3×FLAG-P2A-EGFP, AAV2/9-SYN-3×FLAG-P2A-EGFP, U6-shRNA (Hp1bp3)-CMV-mCherry, and U6-shRNA (NC)-CMV-mCherry were purchased from Obio Technology (Shanghai, China). The AAV/9-SYN-mCherry-5′miR-30a-shRNA (*Cpeb1*)-3′miR-30a, AAV2/6-DIO-Cx3cr1-P2A-mCherry, AAV2/6-CMV-DIO-(EGFP-U6)-shRNA (*Cx3cr1*), and corresponding control virus were purchased from BrainVTA (Wuhan, China). In addition, AAV9-TRE3G-α-gePSI-P2A-β-gePSI-T2A-EGFP-bGHpolyA-CMV-rtTA-hGHpolyA, AAV2/6-CMV-DIO-(EGFP-U6)-shRNA (*Cpeb1*), AAV/9-SYN-Cre-P2A-mCherry, and corresponding control virus were obtained from Brain Case (Shenzhen, China). The correct valid sequence for *sh-Cpeb1* is GCCGAAGGATGCGCTGCAA, for sh-Cx3cr1 is GTTCATGTTCACAAAGAGAAA, and for sh-Hp1bp3 is CAATCTTAACTGAGGCCATTA. All viral vectors were targeted bilaterally to the *IL* region in C57BL/6 J, *Cpeb1^flox/flox^*, and *Cx3cr1*-*Cre* mice at coordinates anterior-posterior (AP): −1.75 mm, medial-lateral (ML): ±0.3 mm, dorsal-ventral (DV): −2.8 mm. hU6-shRNA (*Cpeb1*)-Hef1a-mCherry-3×FLAG and hU6-shRNA (NC)-Hef1a-mCherry-3×FLAG viruses were targeted bilaterally to the *PL* region in C57BL/6 J mice at coordinates AP: −2.4 mm, ML: ±0.25 mm, DV: −1.8 mm, and 0.3 μl of the virus for each site at an infusion rate of 0.03 μl/min using a Hamilton microsyringe (Hamilton). For SUnSET in vivo immunoprecipitation, 3 weeks after the injection of gePSI or control virus, mice underwent a second surgical procedure to implant stainless steel guide cannulas (RWD, China) into the left lateral ventricle (AP: −0.50 mm, ML: −1.10 mm, DV: −2.20 mm). A skull screw was inserted, and dental cement (RWD, China) was used to securely fix the cannula in place. For SUnSET in vivo immunohistochemistry, mice injected with either gePSI or control virus were implanted with two guide cannulas (spaced 0.7 mm apart) in the *IL* region to allow for puromycin infusion.

### Electrophysiology recording

Mice were induced into anesthesia, and coronal brain slices were prepared in artificial cerebrospinal fluid (ACSF) aerated with 95% oxygen and 5% carbon dioxide. The composition of the ACSF solution was 124 mM NaCl, 3.0 mM KCl, 2.0 mM CaCl_2_, 1.2 mM MgSO_4_, 1.25 mM KH_2_PO_4_, 26 mM NaHCO_3_, and 11 mM glucose. Brain sections of 300-μm thickness were obtained using a vibrating microtome (Leica). Subsequently, the slices were incubated in oxygenated ACSF at 30°C for a minimum of 30 min before being transferred to a recording chamber equipped with a planar multielectrode recording setup (MED64, Alpha Med Science, Tokyo, Japan).

For mEPSC recordings, neurons were maintained at −70 mV in voltage-clamp mode. Recordings used an internal solution comprising 140 mM potassium gluconate, 10 mM Hepes, 0.2 mM EGTA, 2 mM NaCl, 2 mM MgATP, and 0.3 mM NaGTP. The external solution contained 10 μM bicuculline (GABA_A_R antagonists), 1 μM tetrodotoxin (sodium channel blocker) and 50 μM AP-5 (NMDAR antagonists). Events were filtered at 2 kHz and sampled at 10 kHz (Molecular Devices, Sunnyvale, CA, USA) using pClamp 10.2 software. Currents were recorded in 10-s epochs for a minimum total duration of 200 s per recording. The electrophysiology experiment was performed as described previously (*115*). Analysis of the mEPSC signal was performed in Clampfit 10.0 software.

### Paired-pulse stimulation

We introduced 10 μM CNQX into the perfusate and positioned the stimulation electrode in mPFC neurons to record evoked somatic currents. The paired-pulse paradigm used a 50-ms interpulse interval, with the objective of assessing the presynaptic release probability of the observed synapses. We administered five pairs of stimuli with a 5-s interval and measured the peak amplitudes of the EPSCs, as described previously (*115*). The paired-pulse ratio was determined by calculating the peak amplitude ratios of the first and second responses. Signal analysis was conducted using Clampfit 10.0 software.

### Golgi staining

Golgi staining was performed with the FD Rapid Golgi Stain Kit (catalog no. PK401, FD Neuro Technologies Inc.) following the manufacturer’s protocol. Mice were anesthetized and underwent perfusion with 0.9% saline, followed by prompt extraction of the brain. After rinsing with double distilled water, the brain was immersed in an impregnation solution (A/B solution in equal parts) and left to incubate in darkness at room temperature for a duration of 2 weeks, with the solution refreshed every 3 days. Subsequently, the brain was transferred to solution C and further incubated in darkness at room temperature for 1 week. Brain sections of 100-μm thickness were obtained using a vibrating microtome (Leica VT1000s) and mounted on high-adhesion glass slides with solution C. These sections were then dehydrated in sequential ethanol solutions for 10 min each and cleared in xylene thrice for 10 min each. The staining procedure was carried out in accordance with established protocols (*32*). Images were captured using a Coolpix 5000 Nikon camera. Sholl analysis and assessment of dendritic spines were performed using Fiji software.

### Sholl analysis

Sholl analysis was conducted to assess the complexity of the dendritic trees and microglia using established methods (*116*, *117*).

### Immunofluorescence

Mice were anesthetized with ketamine (100 mg/kg) and dexmedetomidine (0.5 mg/kg) and perfused with 0.9% saline, followed by 4% paraformaldehyde (PFA) for fixation. Then, brains were extracted and fixed with 4% PFA for an additional 24 hours. Brain slices (30 μm) were then obtained using a vibratome (VT1000S, Leica, Germany). Brain sections underwent three PBS washes and were penetrated with 0.1% Triton X-100 and 5% bovine serum albumin in PBS for 1 hour at room temperature. Subsequently, the sections were incubated with specific primary antibodies overnight at 4°C. Following primary antibody incubation, the sections were washed three times with PBS and then exposed to Alexa Fluor 546– or Alexa Fluor 488–conjugated donkey secondary antibodies (Invitrogen, USA) for 1 hour at room temperature. 4′,6-Diamidino-2-phenylindole (DAPI) staining was performed to visualize cell nuclei. Imaging was conducted using a laser confocal microscope (LSM800, Zeiss, Germany). Immunofluorescence data acquisition was carried out in a blinded manner, with random imaging and cell counting performed in multiple fields per sample to quantify the number of positively stained cells. Primary antibodies used for immunohistochemistry and immunofluorescence are listed in table S2.

### 3D resconstruction

The 3D reconstruction of images was performed using Imaris x64 9.5.0 software (Bitplane Software). Immunofluorescence images were captured with a laser confocal microscope (LSM800, Zeiss, Germany), with the z-stack z-step size adjusted as necessary to encompass the desired range. Subsequently, the acquired 3D images were processed, reconstructed, and analyzed using Imaris x64 9.5.0 software.

### Western blotting

Protein extraction from the mPFC was conducted, and the protein concentration was quantified using the BCA Assay Reagent (Thermo Fisher Scientific, USA). Western blotting was performed as described in previous studies (*112*). The membranes were blocked with 3% defatted milk for 1 hour at room temperature, followed by overnight incubation with primary antibodies at 4°C. Subsequently, protein bands were probed with goat anti-mouse or anti-rabbit antibodies conjugated to IRdye 800 (LI-COR Biosciences, USA) at room temperature for 1 hour. Visualization of protein bands was achieved using Odyssey software (LI-COR Bioscience, USA) after washing with Tris-Buffered Saline with Tween 20 (TBST), and analysis was performed using ImageJ software. Primary antibodies are shown in table S2.

### Surface labeling of translation in vivo

The SUnSET assay was performed as previously described (*11*, *21*), In brief, for the SUnSET in vivo immunoprecipitation, mice injected with gePSI or control virus were fed with Dox or solvent. After 24 hours, 1 μl of 25 μg/μl puromycin (P8833; Sigma-Aldrich) was injected into the left lateral ventricle. One hour after puromycin injection, mPFC tissue was removed and stored at −80°C, following the procedure of the Western blotting. For SUnSET in vivo immunofluorescence, mice injected with gePSI or control virus were treated with DOX or solvent. Twenty-four hours later, puromycin (0.5 μl of 10 μg/μl) was injected into the *IL*. One hour after puromycin injection, the brain was perfused, sliced for staining, and underwent the steps outlined for immunofluorescence staining.

### Real-time PCR

Total RNA was extracted from either cultured cells or brain tissues using TRIzol Reagent (Life Technologies, China) following the manufacturer’s instructions. Reverse transcription PCR (RT-PCR) was carried out as previously detailed (*118*). Briefly, 1 μg of total RNA was reverse transcribed into mRNA or miRNA using either the Reverse Transcription Kit (FSK-100, Toyobo, Japan) or the miRcute Plus miRNA First-Strand cDNA Kit (Tiangen, Beijing, China), respectively. RT-PCR was performed using SYBR Green PCR Master Mix (Takara, Japan) on the CFX96 Real-Time PCR Detection System (Bio-Rad, CA, USA). Gene expression levels were analyzed relative to glyceraldehyde phosphate dehydrogenase using the 2^-ΔΔCt^ method. The primers designed are listed in table S3.

### Poly(A) tail-length assay

Total RNAs were extracted from cells treated with different interventions. Polyadenylation and reverse transcription were carried out using the Poly(A)-Tail Length Assay kit (lot 01272451, Thermo Fisher Scientific, USA) as per the manufacturer’s instructions. Poly(A) tail PCR was conducted using a universal reverse primer and gene-specific forward primers. The poly(A) tail and gene-specific PCR products were separated on a 2% agarose gel. In addition, the poly(A) tail PCR products were sequenced to determine the actual length of poly(A) tails. Gene-specific PCR was performed with a pair of gene-specific primers, and the primers designed are listed in table S4.

### Dual luciferase assay

*Cpeb1* was inserted into the pcDNA3.1(+) vector (Addgene), while the *Hp1bp3* or *Cx3cr1* 3′UTR was inserted into the psiCHECK-2 vector (Promega). Mutated CPE sequences in the *Hp1bp3* or *Cx3cr1* 3′UTR were inserted into the psiCHECK-2 vector using the ClonExpress Ultra One Step Cloning Kit (Vazyme, Nanjing, China, catalog no. C115-02). Subsequently, the psiCHECK-2- *Hp1bp3* or *Cx3cr1* 3′UTR, alone with pcDNA3.1(+) or pcDNA3.1(+)-*Cpeb1*, were cotransfected into 293 T cells cultured in 24-well plates. Luciferase activity was quantified using the Dual Luciferase Reporter Assay Kit (Vazyme, Nanjing, China, catalog no. DL101-01) and measured on the BioTek Synergy2.

### Cell culture

Mouse BV2 cells were brought from American Type Culture Collection (MD, USA), while mouse HT22 cells were brought from the MilliporeSigma (catalog no. SCC129). BV2 cells were cultured in cell culture medium (Pricella, China), whereas HT22 cells were cultured in Dulbecco’s modified Eagle’s medium (Thermo Fisher Scientific, Carlsbad, USA) supplemented with 8% fetal bovine serum (v/v) and 1% penicillin/streptomycin. Cell culture was performed as previously described (*119*). Both cells were incubated at 37°C in a 5% CO_2_ environment. Cells were randomly allocated to different experimental groups. Plasmid transfections were performed using Lipofectamine 3000 (Invitrogen, MA, USA) according to the manufacturer’s instructions.

### Phagocytosis assay

The BV-2 cells were cultured in plates and then randomly assigned to two groups. Each group was transfected with plasmids that silenced *Cx3cr1* gene, along with their respective control groups. In addition, these plasmids carried a red fluorescent marker. After 48 hours, the yellow-green fluorescence-labeled beads (Sigma-Aldrich, USA, L1030) were added for 60 min at 37°C. After that, the cells were rinsed three times with PBS to eliminate any beads that were not engulfed and were then immobilized using 4% PFA. Then, the cells were treated with 0.1% Triton X-100 for 5 min to allow permeabilization, followed by incubation with DAPI (Servicebio, Wuhan, China) for 15 min to stain the cell nuclei. Last, the phagocytosis of beads by microglia was observed using a confocal microscope.

### Statistics

All the statistical analyses were done using GraphPad Prism 8.0 software. All data are shown as means ± SEM. Unpaired or paired *t* tests (two tailed) were used for individual comparisons, while one-way or two-way analyses of variance (ANOVAs) followed by Tukey’s multiple comparisons test were used for single-variable comparisons. The statistical significance level was defined as *P* < 0.05. The number of biological replicates and the statistical tests used in the article are detailed in table S5.

## Supplementary Materials

### The PDF file includes:

Figs. S1 to S13 Tables S1 to S5 Legend for data S1 Uncropped blot and gel images
