# Supplementary material for: Cpeb1 remodels cell type–specific translational program to promote fear extinction
Source: Sci Adv. 2025 Jan 10;11(2):eadr8687. doi: 10.1126/sciadv.adr8687 (PMC11721575; doi:10.1126/sciadv.adr8687)

Science Advances
AAAS

# Supplementary Materials for

## Cpeb1 remodels cell type–specific translational program to promote fear extinction

Juan Zhang *et al.*

Corresponding author: Ling-Qiang Zhu, zhulq@mail.hust.edu.cn; Dan Liu, liudan_echo@mail.hust.edu.cn

*Sci. Adv.* **11**, eadr8687 (2025)
DOI: 10.1126/sciadv.adr8687

**The PDF file includes:**

Figs. S1 to S13
Tables S1 to S5
Legend for data S1
Uncropped blot and gel images

**Other Supplementary Material for this manuscript includes the following:**

Data S1

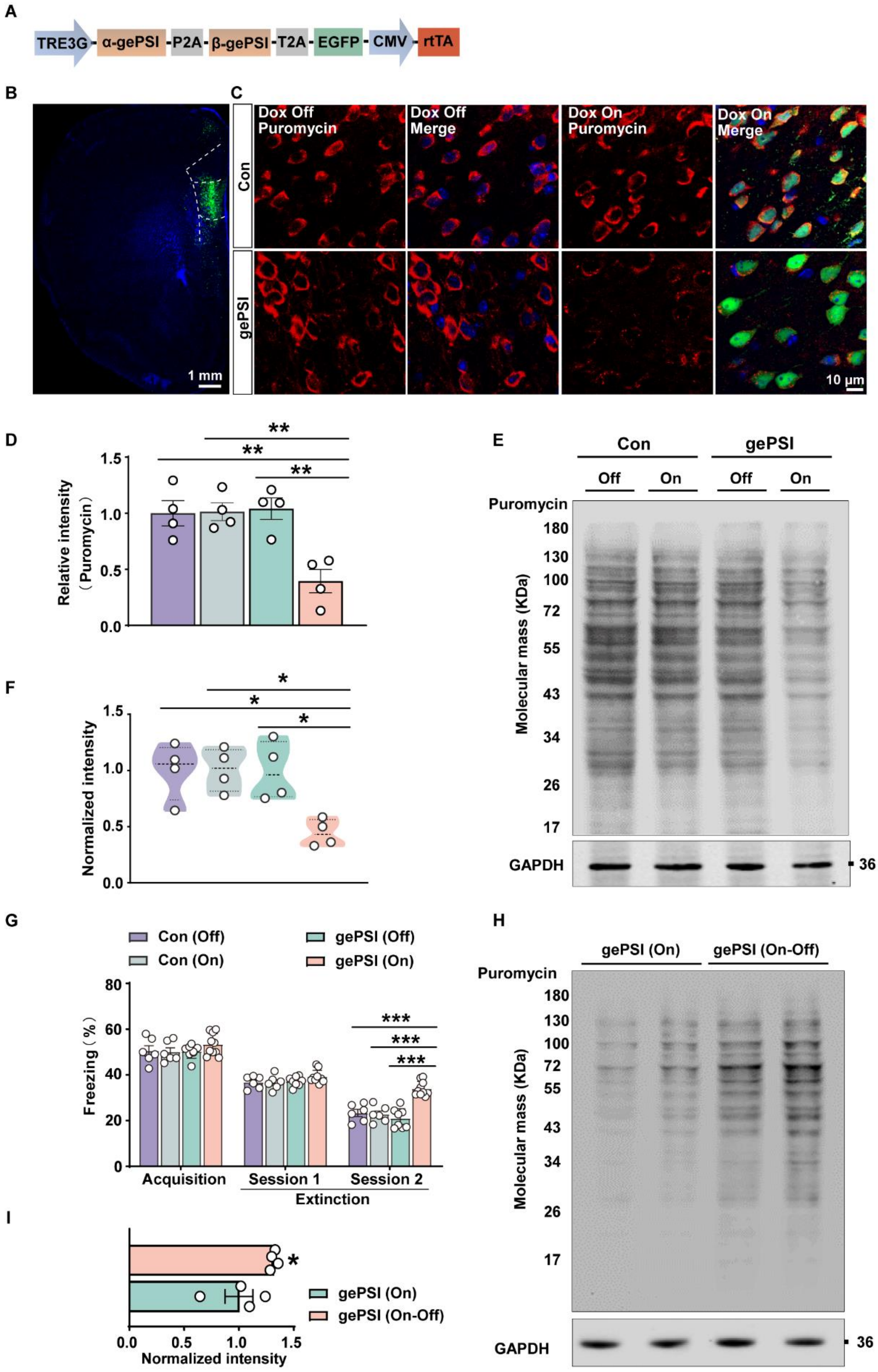


**Figure S1 The effective inhibition of translation in the IL by the gePSI system impairs the fear extinction.**

**(A-B)** Schematic diagram of the virus structure **(A)** and a representative confocal image showing virus injection into the IL from C57BL/6J mice **(B)**.

**(C-D)** In vivo SUnSET assay: Coronal slices from virus infected (EGFP+) neurons or non-infected (EGFP-) neurons from Con (On), Con (Off), gePSI (On), and gePSI (Off) mice were stained with the anti-Puromycin (Red) antibody. Representative images are shown in **(C)** and the analysis of relative intensity was performed in **(D)** (**$p<0.01$, n=4 per group).

**(E-F)** Assessment of newly translated proteins using SUnSET: Results demonstrated that translation was effectively suppressed in the gePSI (On) group. Representative images are shown in **(E)** and the quantitative analysis is shown in **(F)** (*$p<0.05$, n=4 per group).

**(G)** The percentage of freezing during fear memory acquisition and the average percentage of freezing over 14 trials during the two stages of extinction learning are shown for the Con (On), Con (Off), gePSI (On) and gePSI (Off) groups (***$p<0.001$, n=6~10).

**(H-I)** Restoration of protein synthesis in gePSI (On-Off) mice: SUnSET analysis showed that protein synthesis was restored in the gePSI (On-Off) group compared to the gePSI (On) group. Representative images are shown in **(H)** and the quantitative analysis is shown in **(I)** (*$p<0.05$, n=4 per group).

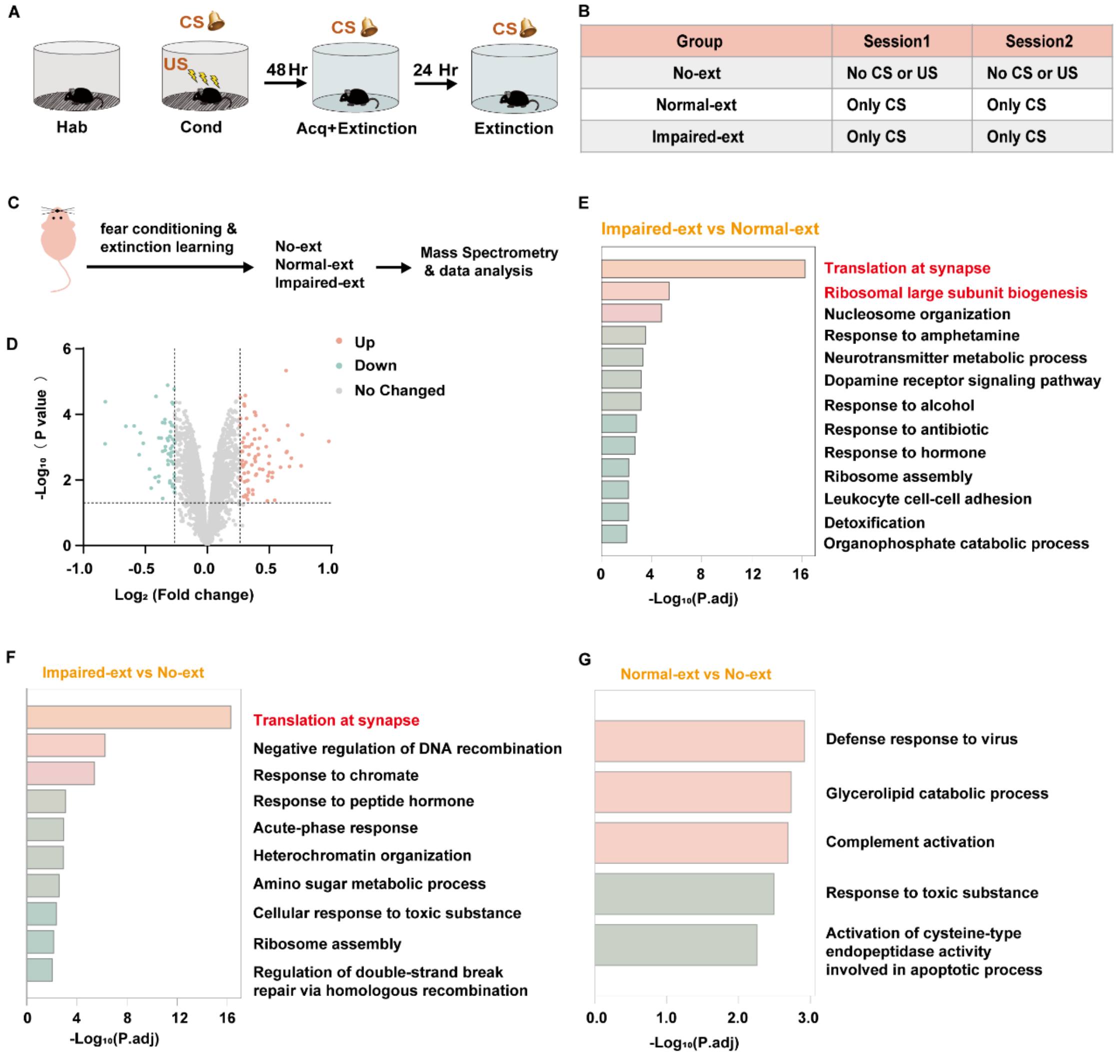


**Figure S2 Proteomic analysis of proteins alteration in the mPFC of mice with impaired fear extinction.**

**(A)** Schematic diagram of the behavioral process for fear conditioning training and fear extinction learning experiments. On day 1, mice were habituated (Hab) and fear conditioned (Cond). On day 2, mice remained in their cages without any intervention. On day 3 and 4, mice were subjected to two trials of extinction tones (Session 1 and Session 2).

**(B)** Schematic diagram of experimental procedure at session 1 and 2 of fear extinction learning in the No-ext, Normal-ext and Impaired-ext groups (CS: conditioned stimuli;

US: unconditioned stimulus).

**(C)** Schematic illustration of the workflow for the proteomic analysis of the mPFC from No-ext, Normal-ext and Impaired-ext groups (n=2 per group).

**(D)** Volcano plot showing the differentially regulated proteins in the mPFC between the Impaired-ext and Normal-ext groups, with fold change > 1.2 (Pink dots) or < -1.2 (Cyan dots) and $p < 0.05$. The gray dots indicate the unchanged proteins.

**(E)** Functional enrichment analysis of differentially expressed proteins between the Impaired-ext group and the Normal-ext group (Fold change > 1.2, $p < 0.05$).

**(F)** Functional enrichment analysis of differentially expressed proteins between the Impaired-ext group and the No-ext group (Fold change > 1.2, $p < 0.05$).

**(G)** Functional enrichment analysis of differentially expressed proteins comparing the Normal-ext group to the No-ext group (Fold change > 1.2, $p < 0.05$).

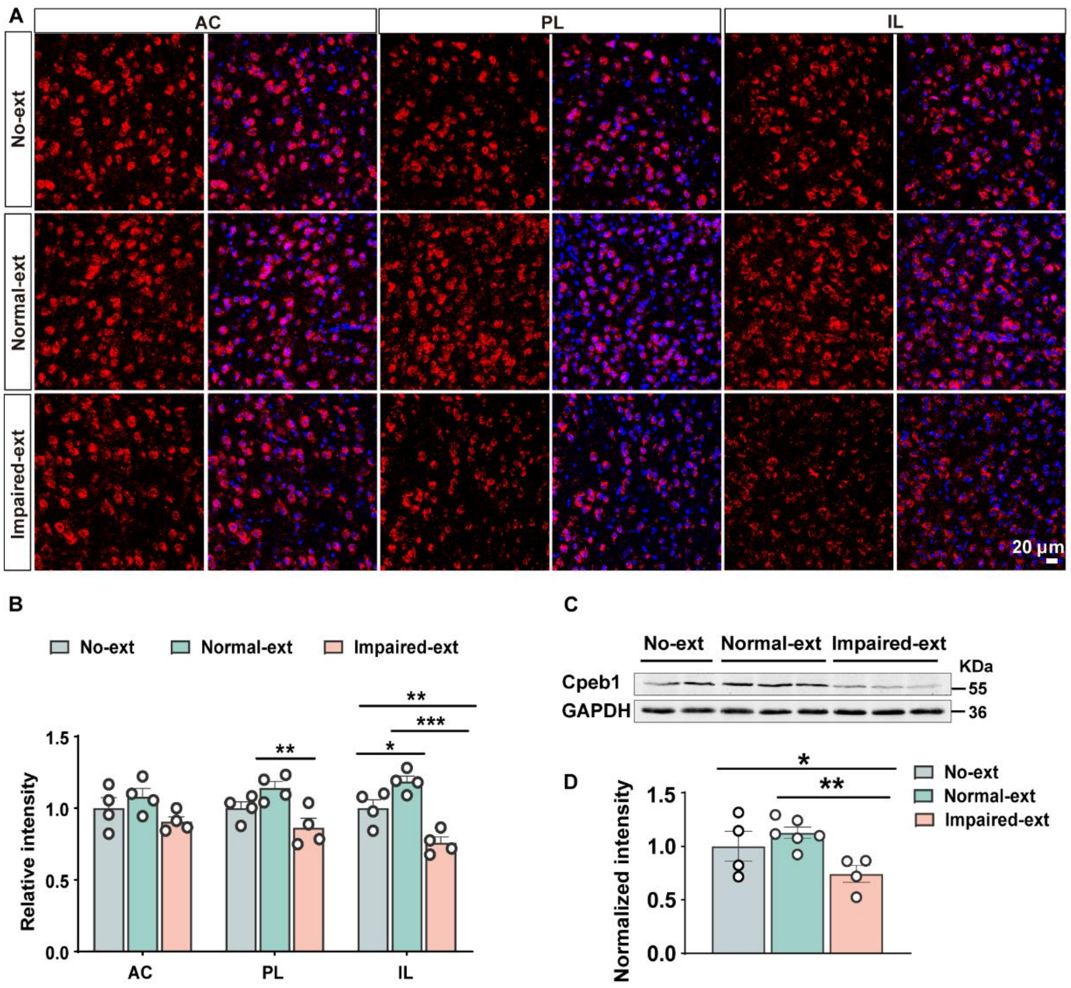


**Figure S3 Significant reduction of Cpeb1 protein in the subregions of the IL in Impaired-extinction mice.**

**(A-B)** Immunofluorescence staining was performed in the subregions of the mPFC sections from the Impaired-ext and Normal-ext group using anti-Cpeb1 (red) antibodies. Representative images were shown in **(A)**, and the relative intensity of Cpeb1 was measured using ImageJ software **(B).** The staining results showed that, compared to the Impaired-ext group, the expression of the translation-related protein Cpeb1 in the PL and IL brain regions of the Normal-ext group was reduced, with a more significant decrease in the IL region. In contrast to the No-ext group, the expression of Cpeb1 in the IL region of the Normal-ext group was upregulated, while

it was downregulated in the IL region of the Impaired-ext group. No such change was observed in the PL region. (*$p<0.05$, **$p<0.01$, ***$p<0.001$, n=4 slices from 4 mice per group).

**(C-D)** The protein levels of Cpeb1 in mPFC homogenates from No-ext, Normal-ext and Impaired-ext mice were examined by western blotting. Representative images are shown in **(C)**, and the quantitative analysis is shown in **(D)** (**$p<0.01$, n=4~6 per group).

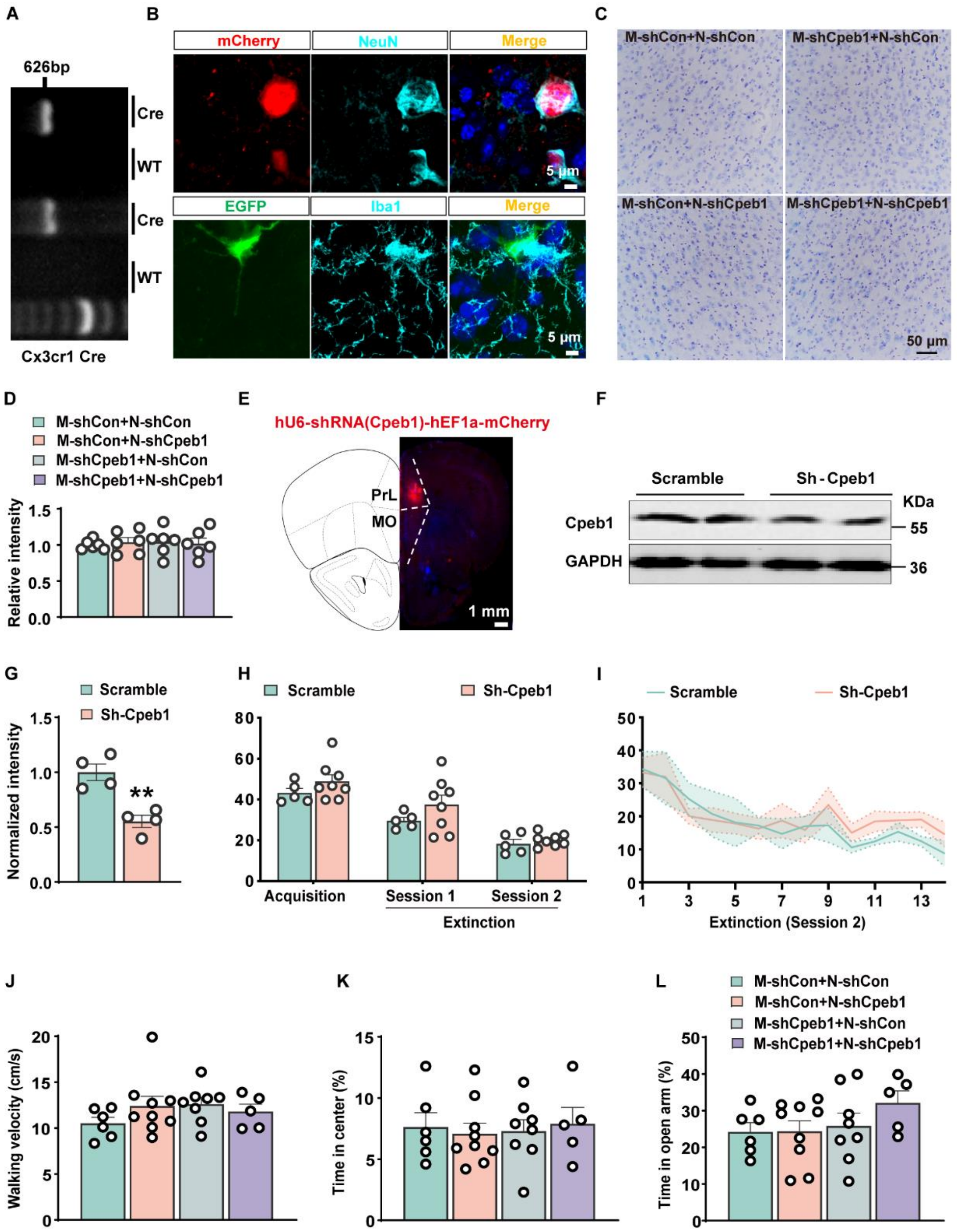


**Figure S4 Knockdown of *Cpeb1* in the PL region does not affect fear extinction in mice.**

**(A)** Genotyping PCR was performed on genomic DNA from *Cx3cr1-Cre* mouse tails, and the sizes of PCR products are provided.

**(B)** Coronal slices from virus-infected (mCherry) neurons were stained with

anti-NeuN (cyan) antibody, and coronal slices from virus infected (EGFP) microglia cells were stained with anti-Iba1 (cyan) antibody.

**(C-D)** The Nissl staining was performed in the IL from the M-shCon+N-shCon, M-shCon+N-shCpeb1, M-shCpeb1+N-shCon and M-shCpeb1+N-shCpeb1 groups. Representative images are shown in panel **(C)**, and the quantitative analysis of Nissl staining is shown in panel **(D)** (n=6 per group).

**(E)** A representative confocal image of virus injection into the PL from C57BL/6J mice is shown.

**(F-G)** The mPFC homogenates were collected and subjected to western blotting assay by using Cpeb1 antibodies **(F)**, and quantitative analysis is shown in panel **(G)** (**$p<0.001$, n=4 per group).

**(H)** Average freezing percentage during fear memory acquisition and in the first and second sessions of fear extinction learning is shown for the Scramble and Sh-Cpeb1 groups (n=5~8 per group).

**(I)** Freezing percentage across 14 trials in the second session of extinction learning is shown for the Scramble and Sh-Cpeb1 groups (n=5~8 per group).

**(J-K)** The percentage of walking velocity (cm/s) **(J)** and time spent in the center **(K)** in the OFT (n=5~9 per group).

**(L)** The percentage of time spent in open arm in the EPM (n=5~9 per group).

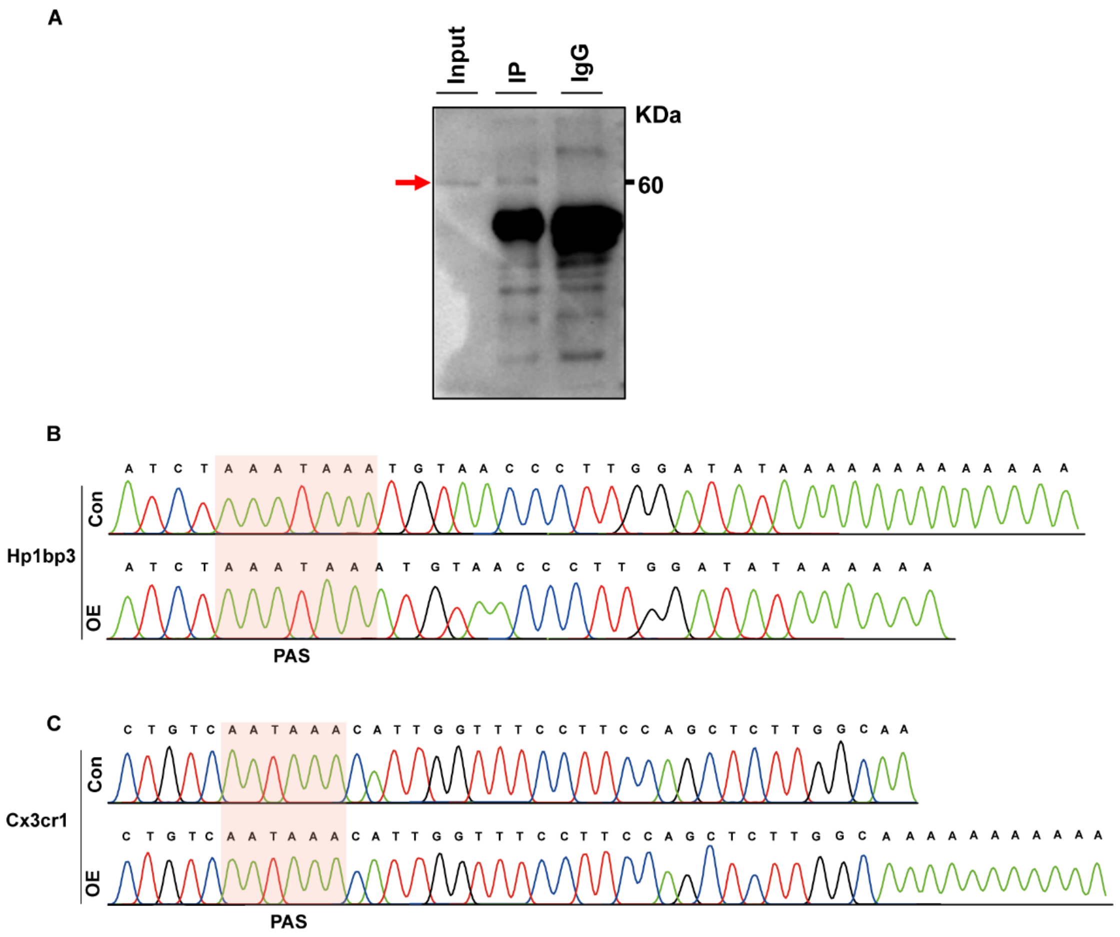


**Figure S5 The sequencing chromatograms of polyadenylated Hp1bp3 and Cx3cr1 transcripts are shown.**

**(A)** Western blot analysis of Cpeb1 was performed following immunoprecipitation with a Cpeb1 antibody. The red arrow indicates the location of the target band.

**(B-C)** Sequencing chromatograms show the polyadenylation of *Hp1bp3*(**B**) and *Cx3cr1*(**C**) transcripts in empty vector (Con)- or Cpeb1 (OE)-transfected HT22 cells or BV2 cells.

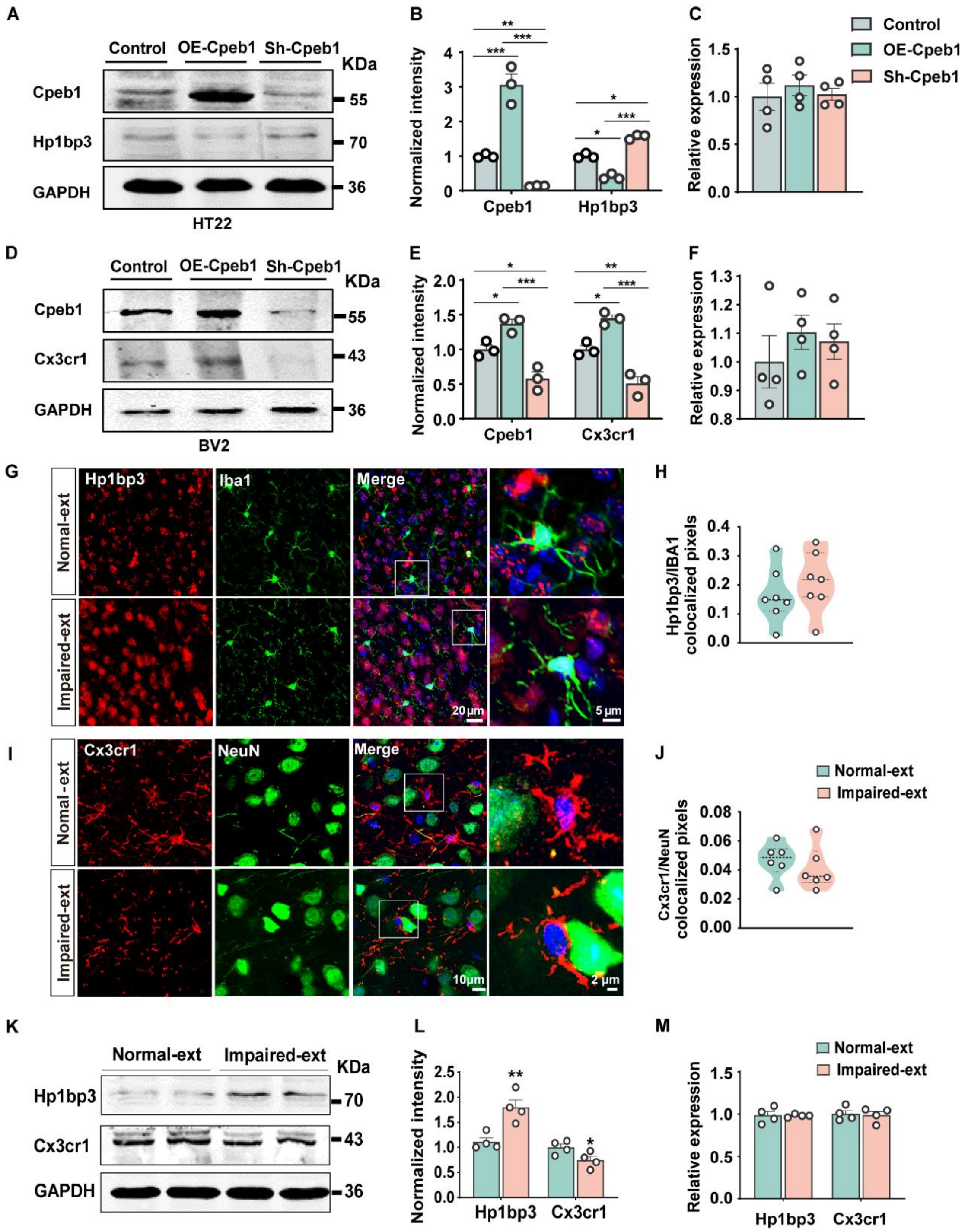


**Figure S6 Cpeb1 regulates Hp1bp3 and Cx3cr1 expression in *vivo* and in *vitro*.**

**(A-C)** HT22 cells were transfected with vector (Control), shRNA against *Cpeb1* (Sh-Cpeb1), or Cpeb1 overexpress plasmid (OE-Cpeb1). Cell lysates were collected 72 hours post-transfection to examine the levels of Cpeb1 and Hp1bp3. Representative images are shown in panel **(A)**, with quantitative analysis in panel **(B)**.

Levels of *Hp1bp3* mRNA were examined by RT-PCR, and the quantitative analysis is shown in panel **(C)** (**p*<0.05, ***p*<0.01, ****p*<0.001, n=3~4 replicates).

**(D-F)** BV2 cells were transfected with vector (Control), shRNA against *Cpeb1* (Sh-Cpeb1), or Cpeb1 overexpression plasmid (OE-Cpeb1). Cell lysates were collected 72 hours post-transfection to examine the levels of Cpeb1 and Cx3cr1. Representative images are shown in panel **(D)**, with quantitative analysis in panel **(F)**. Levels of *Cx3cr1* mRNA were examined by RT-PCR, and the quantitative analysis is shown in panel **(F)** (**p*<0.05, ***p*<0.01, ****p*<0.001, n=3~4 replicates).

**(G-H)** Immunofluorescence staining was performed in the IL sections from the Impaired-ext and Normal-ext groups using anti-Hp1bp3 (Red) and Iba1 (Green) antibodies. Representative confocal images are shown in panel **(G)**, and the quantification of colocalized pixels of Hp1bp3/Iba1 is displayed in panel **(H)** (n=7 cells from four mice per group).

**(I-J)** Immunofluorescence staining was performed in the IL sections from the Impaired-ext and Normal-ext groups using anti-Cx3cr1 (Red) and NeuN (Green) antibodies. Representative confocal images are shown in panel **(I)**, and the quantification of colocalized pixels of Cx3cr1/NeuN is displayed in panel **(J)** (n=6 cells from four mice per group).

**(K-M)** Protein levels of Hp1bp3 and Cx3cr1 in mPFC homogenates from Normal-ext and Impaired-ext mice were examined by western blotting. Representative images are shown in panel **(K)**, with quantitative analysis in panel **(L)** Levels of *Hp1bp3* and *Cx3cr1* mRNA from Normal-ext and Impaired-ext mice were examined by RT-PCR,

and the quantitative analysis is shown in panel **(M)** (*$p<0.05$, **$p<0.01$, n=4 per group).

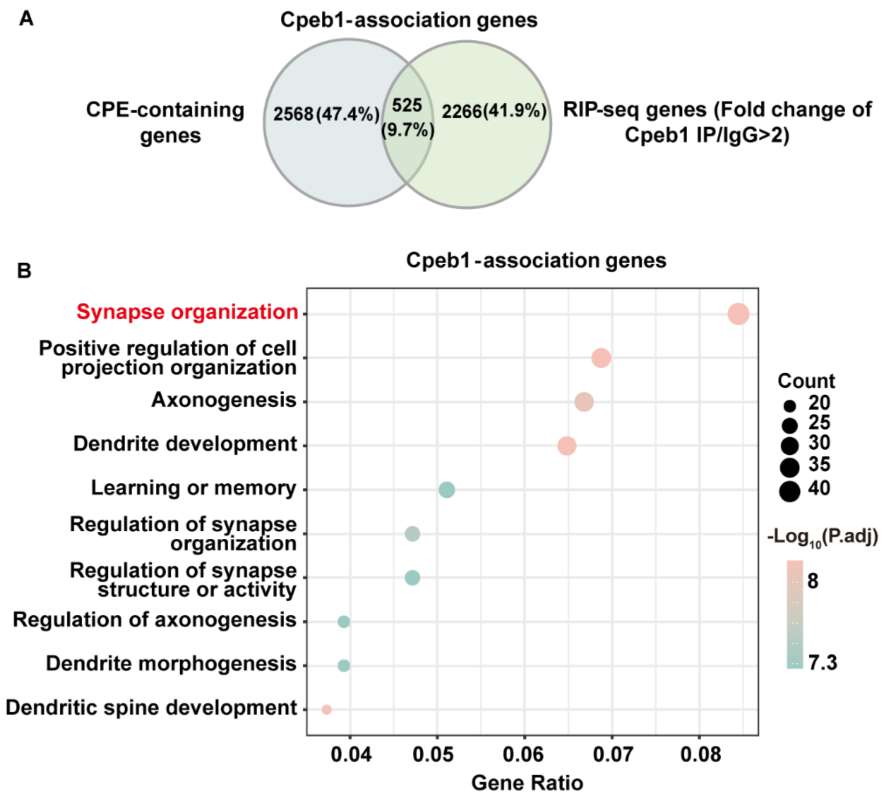


**Figure S7 Cpeb1 regulates transcripts encoding proteins involved in synapse organization, dendrite development, and leaning.**

**(A)** Venn diagram illustrating the overlap between CPE-containing genes and genes identified through RIP-seq (fold change of IP/IgG >2).

**(B)** Functional enrichment analysis of genes associated with Cpeb1.

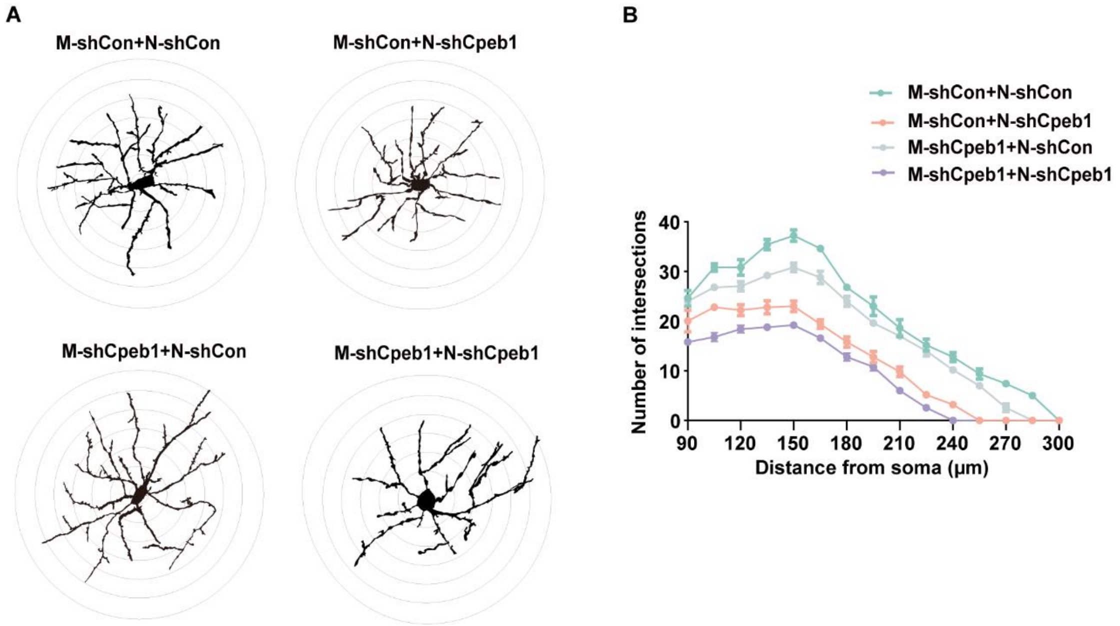


**Figure S8 Neuron- and microglia-specific Cpeb1-dependent translational signatures are necessary for synaptic function.**

**(A-B)** Two-dimensional reconstructions of IL neurons infected with the virus from M-shCon+N-shCon, M-shCon+N-shCpeb1, M-shCpeb1+N-shCon, M-shCpeb1+N-shCpeb1 mice are shown in panel **(A)**. Sholl analysis was performed to evaluate the dendritic complexity, and the results are shown in panel **(B)** (n=5 neurons from four mice per group).

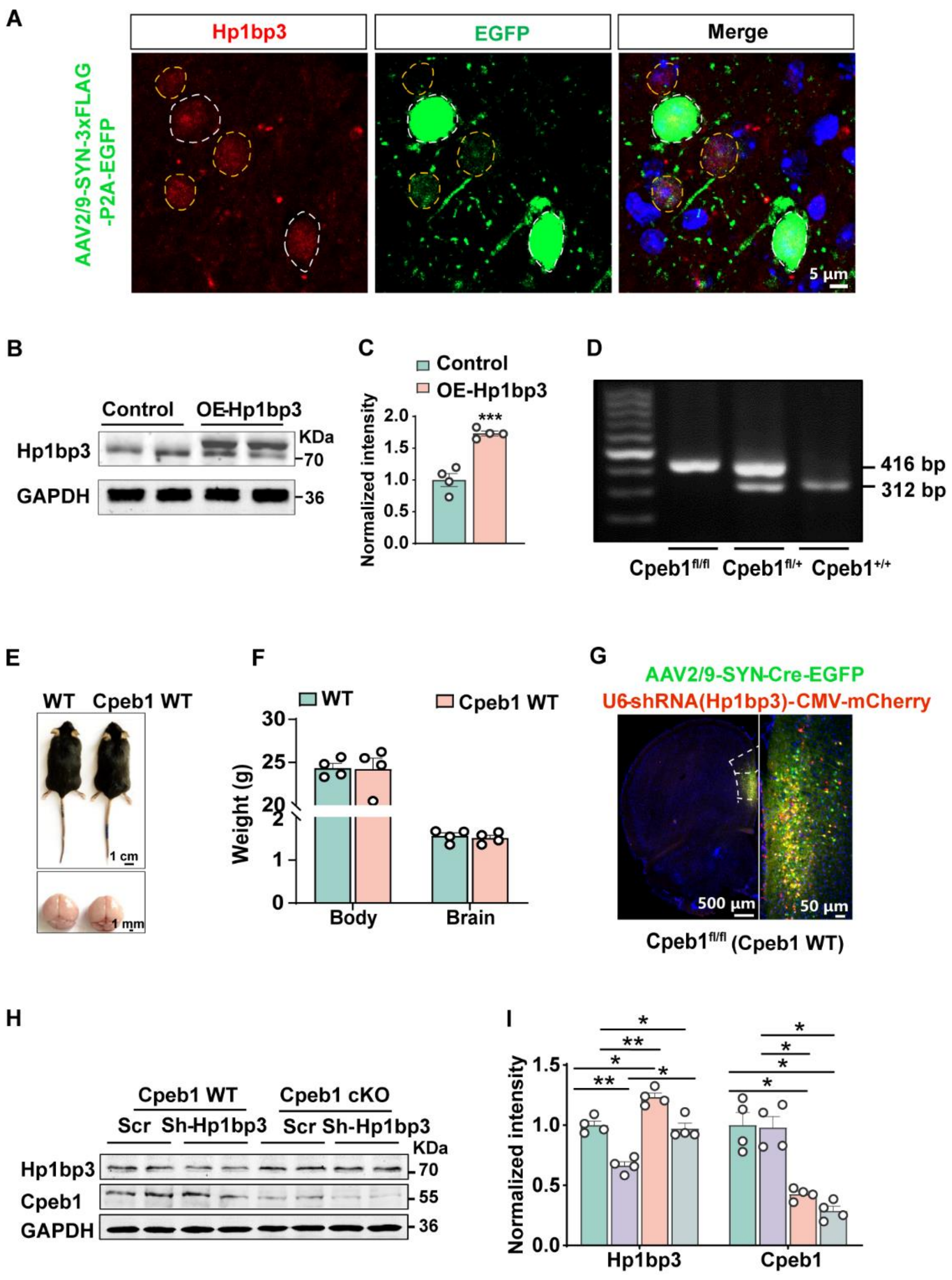


**Figure S9 Neuronal overexpression of Hp1bp3 and neuronal-specific inhibition of Hp1bp3 are effective.**

**(A)** Control mice were injected with the control virus AAV2/9-SYN-3×FLAG-P2A-EGFP into the IL of C57BL/6J mice (Control). Coronal slices from virus-infected (EGFP+) neurons were stained with an anti-Hp1bp3 (red) antibody. The white dashed line indicates neurons with virus infection, while the

yellow dashed line indicates neurons without virus infection.

**(B-C)** Protein levels of Hp1bp3 in mPFC homogenates from control and OE-Hp1bp3 mice were examined by western blotting. Representative images are shown in panel **(B)**, and the quantitative analysis is shown in panel **(C).** (***$p$<0.001, n=4 per group).

**(D)** Genotyping PCR was performed on genomic DNA from *Cpeb1*$^{fl/fl}$ (Cpeb1 WT) mouse tails, and the sizes of PCR products are provided.

**(E-F)** Representative images of body size and brain size from the wide-type and Cpeb1 WT mice are showed in panel **(E)**. The body and brain weights of the wide-type and Cpeb1 WT mice are quantified in panel **(F)**.

**(G)** Representative confocal image (right) showing virus injection into the IL of *Cpeb1*$^{fl/fl}$ (Cpeb1 WT) mice.

**(H-I)** mPFC homogenates were collected and subjected to a western blotting assay using antibodies against Cpeb1 and Hp1bp3 **(H)**, and quantitative analysis is shown in panel **(I)** (*$p$<0.05, **$p$<0.001, n=4 per group).

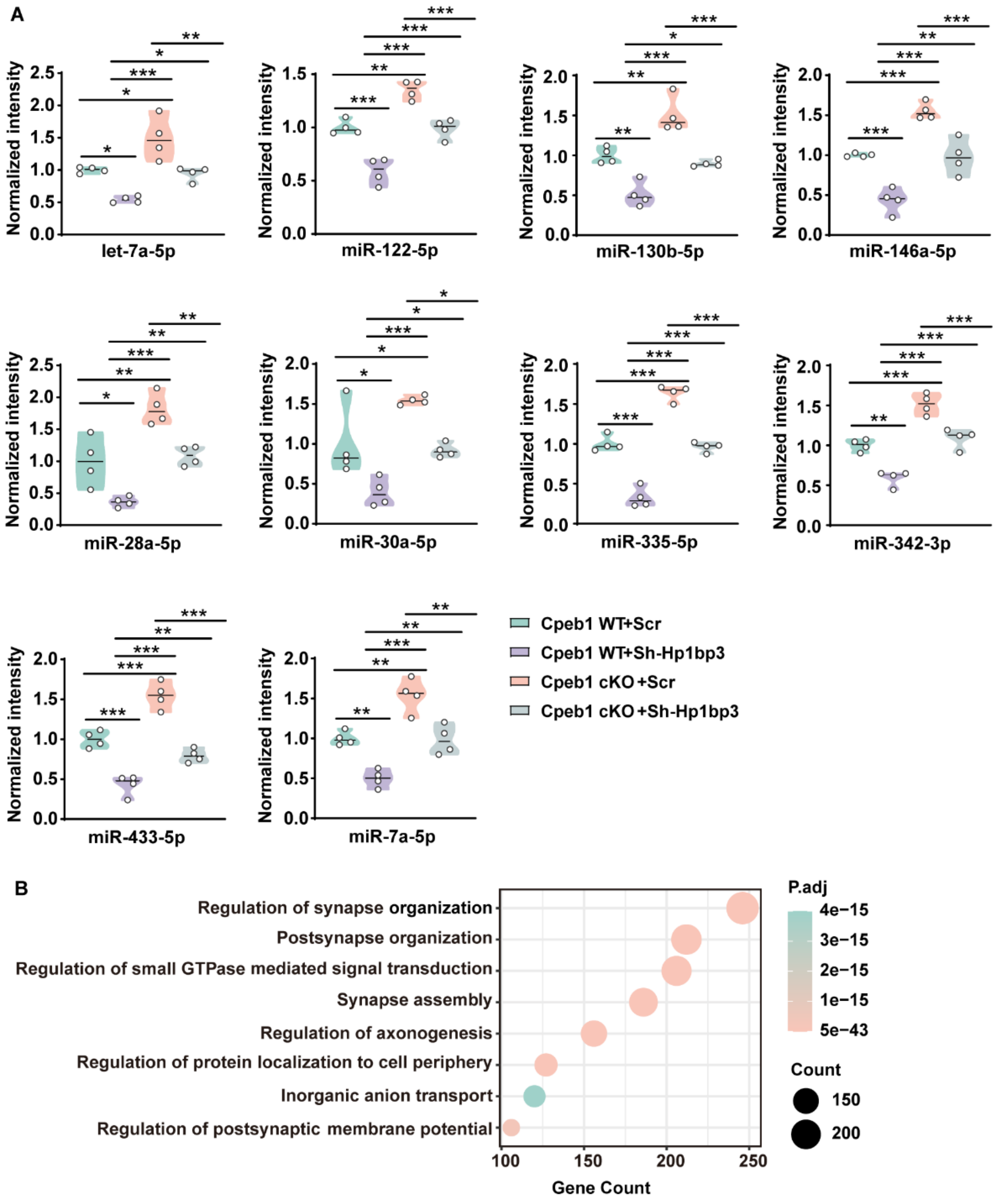


**Figure S10 The complex miRNA networks regulate postsynaptic proteins.**

**(A)** qPCR was performed to measure the relative expression levels of let-7a-5p, miR-122-5p, miR-130b-5p, miR-146a-5p, miR-28a-5p, miR-30a-5p, miR-335-5p, miR-342-3p, miR-433-5p, miR-7a-5p in mPFC tissues from Cpeb1 WT+Scr, Cpeb1 cKO+Scr, Cpeb1 WT+Sh-Hp1bp3, and Cpeb1 cKO+Sh-Hp1bp3 mice (*$p$<0.05, **$p$<0.01, ***$p$<0.001, n=4).

**(B)** Functional enrichment analysis was performed for the predicted targets of these

10 miRNAs.

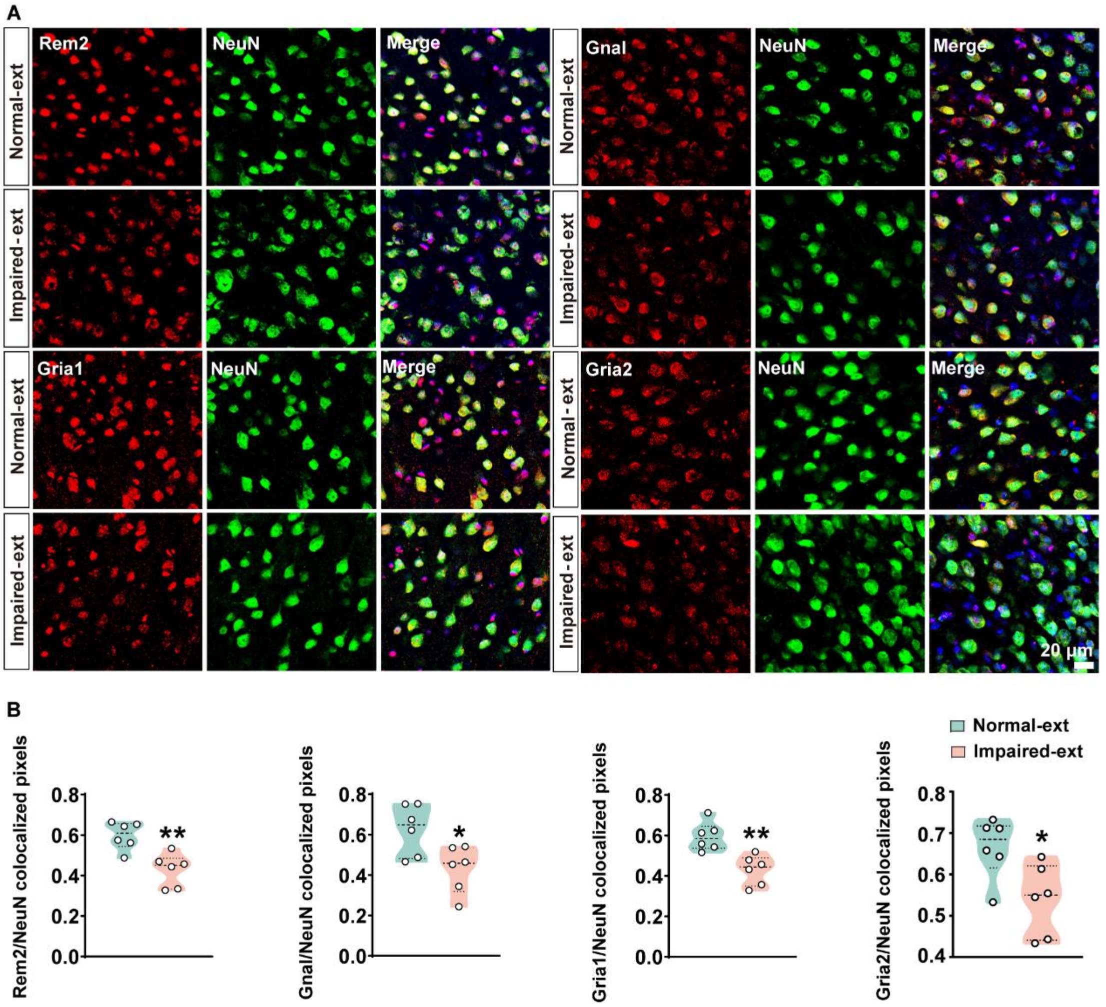


**Figure S11 The targets regulated by the miRNA networks are reduced in the IL neurons of Impaired-extinction mice.**

**(A-B)** Coronal slices from the Impaired-ext and Normal-ext groups were stained with anti-NeuN (Green) and either anti-Rem2, anti-Gnal, anti-Gria1 and anti-Gria2 (Red) antibodies. Representative images are shown in **(A)**, and colocalized pixels were quantified in **(B)** (*$p$<0.05, **$p$<0.01, n= 6 cells from four mice per group).

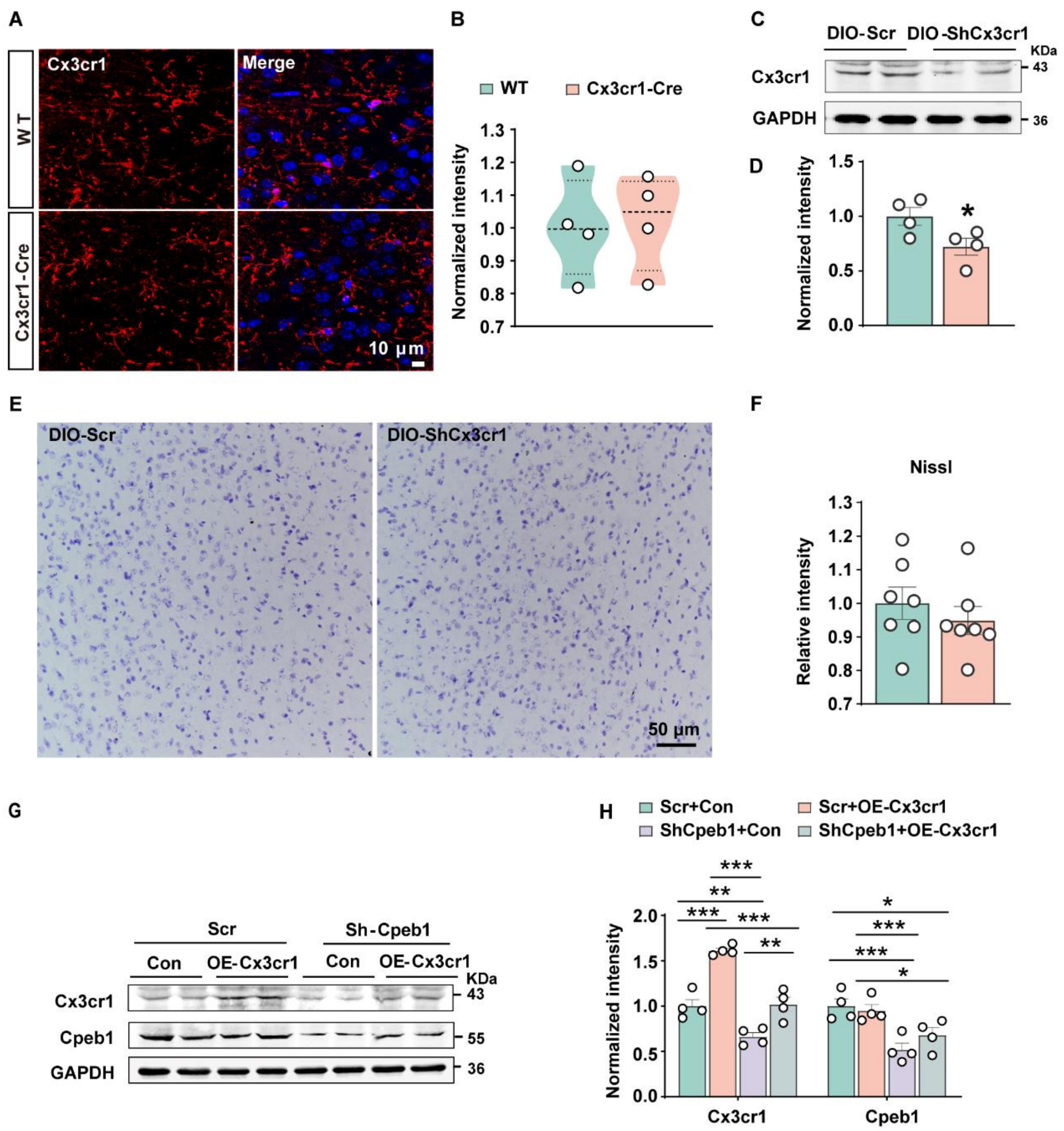


**Figure S12 Inhibition of microglial Cx3cr1 and microglia-specific overexpression of Cx3cr1 are effective.**

**(A-B)** Immunofluorescence staining was performed on IL sections from the WT and *Cx3cr1-Cre* groups using anti-Cx3cr1 (Red) and DAPI (Blue) antibodies (Bar= 10 μm). Representative confocal images are shown in **(A)**, and the normalized intensity is displayed in **(B)** (n=4 per group).

**(C-D)** The protein levels of Cx3cr1 in mPFC homogenates from the DIO-Scr and DIO-Sh-Cx3cr1 groups were examined by western blotting. Representative images

are shown in **(C)**, and the quantitative analysis is shown in **(D)** (*$p<0.05$, n=4 per group).

**(E-F)** Nissl staining was performed on IL sections from DIO-Scr and DIO-Sh-Cx3cr1 groups. Representative images are shown in **(E)**, and the quantitative analysis of Nissl staining is displayed in **(F)** (n=7 per group).

**(G-H)** mPFC homogenates were collected from the Scr+Con, Scr+OE-Cx3cr1, Sh-Cpeb1+Con, and Sh-Cpeb1+OE-Cx3cr1 groups and subjected to western blot analysis using antibodies against Cpeb1 and Cx3cr1 **(G)**, with quantitative analysis shown in **(H)** (*$p<0.05$, **$p<0.01$, ***$p<0.001$, n=4 per group).

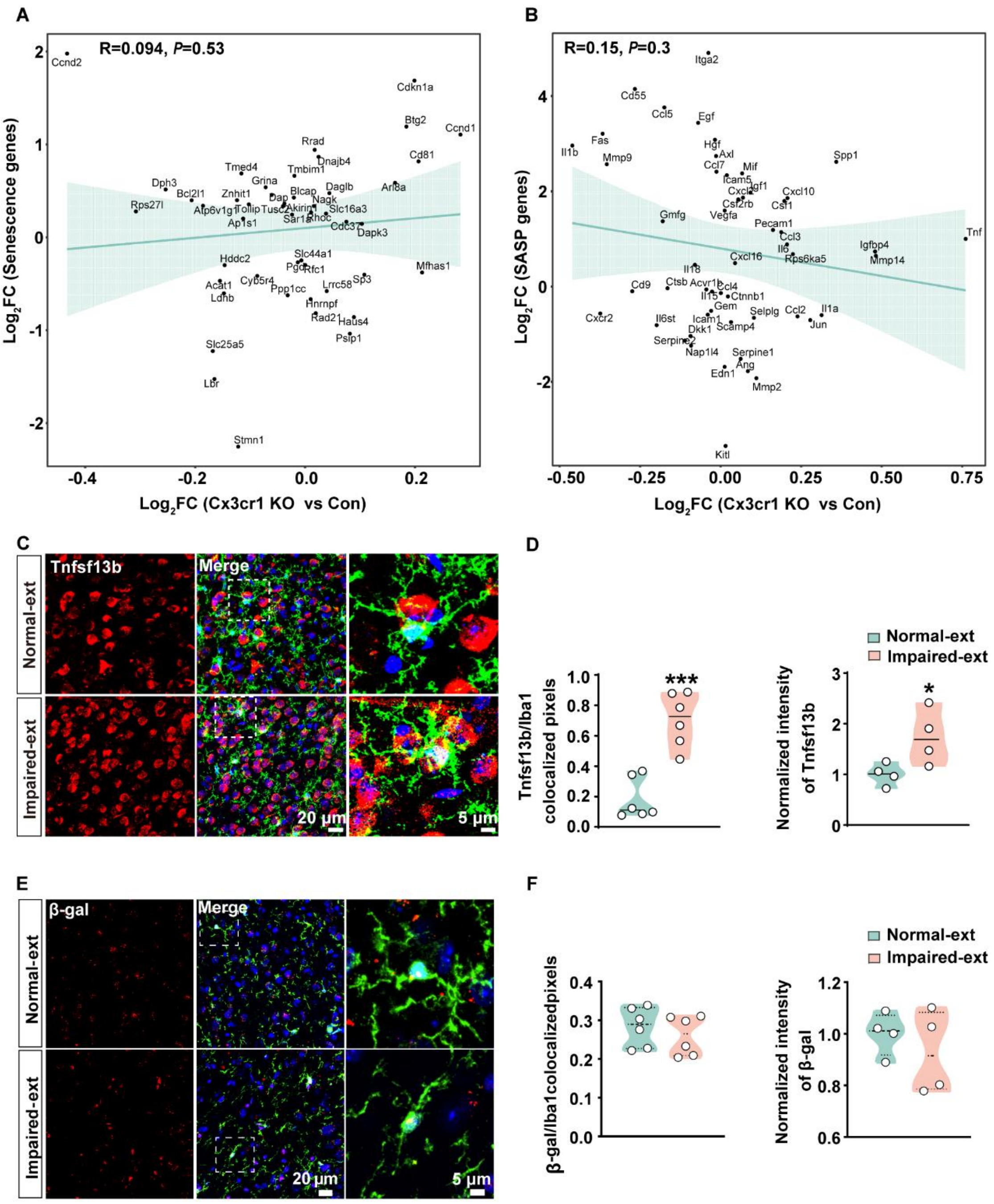


**Figure S13 Aged-like microglia in the Impaired-extinction group do not exhibit a senescent phenotype.**

**(A)** Scatter plot showing the linear regression of $log_2$FC values for overlapping genes between well-known senescence gene sets and gene sets of *Cx3cr1*-deficient microglia, revealing no significant correlation. Green shading represents the 95% confidence interval, and Pearson's correlation coefficient with the

corresponding *P*-value is shown at the top.

**(B)** Scatter plot displaying the correlation between well-defined SASP (Senescence-associated secretory phenotype) genes and genes of *Cx3cr1*-deficient microglia, suggesting that SASP genes are not associated with Cx3cr1-deficient microglia.

**(C-D)** Coronal slices from the Impaired-ext and Normal-ext group were stained with anti-Tnfsf13b (Red) with anti-Iba1 (Green) antibodies in the IL. Representative images are shown in **(C)**, and colocalized pixels and relative intensity are quantified in **(D)** (*$p$<0.01, ***$p$<0.001, n= 6 cells from four mice per group).

**(E-F)** Coronal sections from the Impaired-ext and Normal-ext groups were stained with anti-β-gal (Red) and anti-Iba1 (Green) antibodies in the IL. Representative images are shown in **(E)**, and colocalized pixels with relative intensities are quantified in **(F)** (n= 6 cells from four mice per group).

**Table S1. The predicted targets of the miRNAs**

| miRNA name | miRNAs targets (Targetscan) ∩Proteome (Fold change< -1.2&*p*<0.05) |
|---|---|
| let-7a-5p | Pbx1, Smarcc1, Ubald1, Aldh1a1, Cox17, Fibcd1, Gnal, Hsd11b1, Jpt1, Meis3, Mup2, Myoz3, Nmbr, Nxn, Pde10a, Txnl4b |
| miR-122-5p | Mt1, Ubald1, Ankrd63, Lancl3, Adcy5, Aldh1a1, Cox17, Fibcd1, Pbx1, Prelid3a, Rem2, Gria1, Slc35f1 |
| miR-130b-5p | Pbx1, Xaf1, Aldh1a1, Myoz3, Nxn, Smarcc1, Ankrd63, Cox17, Gnpnat1, Phip, Prelid3a, Slc35f1, Ubald1, Gria1, Gria2 |
| miR-146a-5p | Pbx1, Pde10a, Ubald1, Adcy5, Gnal, Lancl3, Mt1, Nxn, Slc35f1, Gria2, Xaf1 |
| miR-28a-5p | Cox17, Ubald1, Sun2, Fibcd1, Fv1Islr, Lancl3, Mt1, Nxn, Gria2, Pbx1 |
| miR-30a-5p | Ankrd63, Lancl3, Mt1, Nmbr, Nxn, Pde10a, Phip, Slc35f1, Srp19, Ubald1, Gria1, Gria, 2Xaf1 |
| miR-335-5p | Lancl3, Mt1, Pbx1, Pde1b, Prelid3a, Scgn, |

| | |
|---|---|
| | Sun2, Ubald1, Xaf1, Adcy5, Ankrd63, Islr, Nmbr, Gria2, Th |
| miR-342-3p | Prelid3a, Aldh1a1, Cox17, Fibcd1, Lancl3, Mt1, Myoz3, Pde10a, Rem2, Scgn, Gria1, Txnl4b |
| miR-433-5p | Gnal, Lancl3, Pde10a, Gria1, Rem2 |
| miR-7a-5p | Mt1, Pbx1, Sun2, Adcy5, Ankrd63, Cox17, Lancl3, Pde10a, Pde1b, Prelid3a, Scgn, Ubald1, Gria2, Xaf1 |

**Table S2. Antibodies used in the study**

| Antibody | Catalog number/manufacturer | Dilution |
|---|---|---|
| Cpeb1 | 13274-1-AP, Proteintech; ab181051, Abcam | 1:1000 for WB<br>1:500 for IF |
| Puromycin | MABE343, Millipore | 1:1000 for WB<br>1:500 for IF |
| Hp1bp3 | 24556-1-AP, Proteintech | 1:1000 for WB<br>1:500 for IF |
| Cx3cr1 | DF7096, Affinity<br>149002, BioLegend | 1:1000 for WB<br>1:600 for IF |
| Aggrecan | 13880-1-AP, Proteintech | 1:200 for IF |
| Rem2 | DF9553, Affinity Biosciences | 1:800 for WB<br>1:200 for IF |
| IBA1 | Ab283346, Abcam | 1:600 for IF |
| Xaf1 | Ab17204, Abcom | 1:600 for IF |
| Tnfsf13b | NBP1-76843, Novus | 1:600 for IF |

| | | |
|---|---|---|
| | Biologicals | |
| Gnal | PA5-101625, Thermo Fisher | 1:1000 for WB<br>1:600 for IF |
| Gria1 | 67642-1-Ig, proteintech | 1:1000 for WB<br>1:600 for IF |
| Gria2 | MAB397, Milipore | 1:1000 for WB<br>1:600 for IF |
| Beta galactosidase | A-11132, Thermo fisher | 1:600 for IF |
| GAPDH | 60004-1-Ig, proteintech | 1:3000 for WB |

IF, Immunofluorescence;

WB, Western blot.

**Table S3. Sequences of mRNA and miRNA primers**

| Target gene | Species | primer sequence |
|---|---|---|
| Cpeb1 forward | mus | AAGAACCCCATCTACTCA |
| Cpeb1 reverse | mus | GCTTGCCATCCTTACCAG |
| Hp1bp3 forward | mus | CTATACGTCGAGCCGTGAAT |
| Hp1bp3 reverse | mus | CCAGGCAGGAATCGTCTTTT |
| Cx3cr1 forward | mus | GAGCATCACTGACATCTACCTCC |
| Cx3cr1 reverse | mus | AGAAGGCAGTCGTGAGCTTGCA |
| let-7a-5p forward | mus | TGAGGTAGTAGGTTGTATAGTT |
| let-7f-5p forward | mus | TGAGGTAGTAGATTGTATAGTT |
| miR-122-5p forward | mus | TGGAGTGTGACAATGGTGTTTG |
| miR-129-5p forward | mus | CTTTTTGCGGTCTGGGCTTGC |
| miR-130b-5p forward | mus | ACTCTTTCCCTGTTGCACTACT |
| miR-146a-5p forward | mus | TGAGAACTGAATTCCATGGGTT |
| miR-151-5p forward | mus | TCGAGGAGCTCACAGTCTAGT |
| miR-203-3p forward | mus | GTGAAATGTTTAGGACCACTAG |
| miR-28a-5p forward | mus | AAGGAGCTCACAGTCTATTGAG |
| miR-30a-5p forward | mus | TGTAAACATCCTCGACTGGAAG |

| | | |
|---|---|---|
| miR-335-5p forward | mus | TCAAGAGCAATAACGAAAAATGT |
| miR-342-3p forward | mus | TCAAGAGCAATAACGAAAAATGT |
| miR-383-5p forward | mus | AGATCAGAAGGTGACTGTGGCT |
| miR-433-5p forward | mus | TACGGTGAGCCTGTCATTATTC |
| miR-448-3p forward | mus | TTGCATATGTAGGATGTCCCAT |
| miR-672-5p forward | mus | TGAGGTTGGTGTACTGTGTGTGA |
| miR-7a-5p forward | mus | TGGAAGACTAGTGATTTTGTTGT |
| miR-871-3p forward | mus | TGACTGGCACCATTCTGGATAAT |
| miR-365-2-5p forward | mus | AGGGACTTTCAGGGGCAGCTGTG |
| pre-let-7a forward | mus | TTCACTGTGGGATGAGGT |
| pre-let-7a reverse | mus | ATCACCTTAGGAAAGACAGTAG |
| pre-miR-122 forward | mus | AGCTGTGGAGTGTGACA |
| pre-miR-122 reverse | mus | AGCTATTTAGTGTGATAATGGCG |
| pre-miR-130b forward | mus | GGCTTGTTGGACACTCTTTC |
| pre-miR-130b reverse | mus | GGCCCGACAGATGCC |
| pre-miR-146a forward | mus | AGCTCTGAGAACTGAATTCCA |
| pre-miR-146a reverse | mus | AGCTGAAGAACTGAATTTCACAG |
| pre-miR-28a forward | mus | GGTCCCTACCTTCAAGGAGC |
| pre-miR-28a reverse | mus | AGTGCCTGCCCTCCAG |
| pre-miR-30a forward | mus | GCGACTGTAAACATCCTCGA |
| pre-miR-30a reverse | mus | GCAGCTGCAAACATCCG |
| pre-miR-335 forward | mus | TCTTTTGGGCGGGGGT |

| | | |
|---|---|---|
| pre-miR-335 reverse | mus | TGGCTATAACCCATGAGAGGG |
| pre-miR-342 forward | mus | GAAAATGGGCTCAAGGTGAG |
| pre-miR-342 reverse | mus | TCAGCAGGCCAAGGTG |
| pre-miR-433 forward | mus | TGCCCGGGGAGAAGT |
| pre-miR-433 reverse | mus | GGGCTGCCTTCATGGT |
| pre-miR-7a forward | mus | TTGGATGTTGGCCTAGTTCTG |
| pre-miR-7a reverse | mus | CTGTAGAGGTGGCCTGTG |
| pri-let-7a forward | mus | TGAGCCATATCGTCTCTGCTTCTAG |
| pri-let-7a reverse | mus | CTGGAATAAGCATCAGTCTCTTTCAAG |
| pri-miR-122 forward | mus | CAGTTTCAGCGTTTGGAACC |
| pri-miR-122 reverse | mus | TACATTACACACAATGGAGAACTC |
| pri-miR-130b forward | mus | TCACCTACTGTTCCTTCTTTCTTCCC |
| pri-miR-130b reverse | mus | CTCTGCAGGCTTCTTGTTCCAA |
| pri-miR-146a forward | mus | CAATCCAGTCATCTCTGCTATTGATG |
| pri-miR-146a reverse | mus | CCTCTTTGTTGAGACACAATCTCACT |
| pri-miR-28a forward | mus | CCTATGCAATGTCATTTAGCCTTAGG |
| pri-miR-28a reverse | mus | GTACAATCAAATCAACATGTCCTGCC |
| pri-miR-30a forward | mus | AACATCAGCTGACTGTACAGAGC |
| pri-miR-30a reverse | mus | CTAAACCAAGAAGGGGTAGGTGG |
| pri-miR-335 forward | mus | CAACAGCATTTGGAGATAGGTGC |
| pri-miR-335 reverse | mus | CCTATGCAAATAAGATGTAACACTGA |
| pri-miR-342 forward | mus | ATTGTGCACCTTGCAAACAT |

| | | |
|---|---|---|
| pri-miR-342 reverse | mus | TCCTTCCACAGCTCACACAC |
| pri-miR-433 forward | mus | GCCTCTCTGGTTCCAACATCT |
| pri-miR-433 reverse | mus | GACGAAACTGGCAAGAAATCTACC |
| pri-miR-7a forward | mus | GAAACTGGAAGCTGTTAATACTTGGT |
| pri-miR-7a reverse | mus | CCTTAACCAAGCAAACTTCTCATT |
| Universal R primer | mus | GCTGTCAACGATACGCTACG |
| U6 | mus | GATGACACGCAAATTCGTGAA |
| GAPDH forward | mus | TGTTTCCTCGTCCCGTAG |
| GAPDH reverse | mus | CAATCTCCACTTTGCCACT |

**Table S4. Gene-specific primers for Poly(A) tail-length assay**

| Target gene | Species | primer sequence |
|---|---|---|
| Hp1bp3 forward | mus | AGATGCTGGAAGCAAGGTGG |
| Hp1bp3 reverse | mus | ACCAAGCCTGTCCTTTCTGG |
| Cx3cr1 forward | mus | ATGGCAGGCTTAGTCAAT |
| Cx3cr1 reverse | mus | TGCCAAGAGCTGGAAGGA |

**Table S5 Statistics.**

**Fig 1-9**

| **Fig** | **Statistic Methods** | **Comparison** | **Number** | **Statistics** | **P value** |
|---|---|---|---|---|---|
| **1B** | Unpaired t test | Session 2 | 8,7 | | <0.0001 |
| | One-way ANOVA, Tukey's multiple comparisons test | Session 3 | | | |
| | | - | 13,210 (Fn,Fd) | F=9.228 | <0.0001 |
| | | gePSI (Off) vs gePSI (On-Off) | 8,5 | | 0.0131 |
| | | gePSI (Off) vs gePSI (On) | 8,5 | | <0.0001 |
| | | gePSI (On-Off) vs gePSI (On) | 5,5 | | <0.0001 |
| | One-way ANOVA, Tukey's multiple comparisons test | Session 4 | | | |
| | | - | 13,210 (Fn,Fd) | F=3.833 | <0.0001 |
| | | gePSI (Off) vs gePSI (On) | 8,5 | | <0.0001 |
| | | gePSI (On-Off) vs gePSI (On) | 5,5 | | <0.0001 |
| **1C** | One-way ANOVA, Tukey's multiple comparisons test | Session 2 | | | |
| | | - | 2,39 (Fn,Fd) | F=64.76 | <0.0001 |
| | | Normal-ext vs Impaired-ext | 4,4 | | <0.0001 |
| | | No-ext vs Impaired-ext | 4,4 | | <0.0001 |
| **2B** | Unpaired t test | Relative intensity (Cpeb1) | 5,5 | | 0.005 |
| | Unpaired t test | Cpeb1/NeuN | 6,6 | | 0.0044 |
| | Unpaired t test | Cpeb1/Iba1 | 6,6 | | 0.0285 |
| **2E** | Two-way ANOVA, Tukey's multiple comparisons test | - | 1.365,10.92 (Fn,Fd) | F=26.75 | 0.0002 |
| | | Session 1 M-shCon+N-shCon vs M-shCon+N-shCpeb1 | 6,9 | | 0.0486 |
| | | Session 2 M-shCon+N-shCon vs M-shCon+N-shCpeb1 | 6,9 | | 0.0094 |
| | | M-shCon+N-shCon vs M-shCpeb1+N-shCon | 6,8 | | 0.0324 |
| | | M-shCon+N-shCon vs M-shCpeb1+N-shCpeb1 | 6,5 | | 0.0056 |

| | | | | | |
|---|---|---|---|---|---|
| | | M-shCon+N-shCpeb1 vs M-shCpeb1+N-shCpeb1 | 9,5 | | 0.0418 |
| | | M-shCpeb1+N-shCon vs M-shCpeb1+N-shCpeb1 | 8,5 | | 0.0155 |
| **3D** | Multiple t test adjusted with Holm-Sidak | Seg.1 | 3,3 | | 0.000153 |
| | | Mut1-Seg.1 | 3,3 | | 0.006466 |
| | | Seg.3 | 3,3 | | 0.006835 |
| | | Mut4-Seg.3 | 3,3 | | 0.0008 |
| | | WT | 3,3 | | <0.0001 |
| **3E** | Multiple t test adjusted with Holm-Sidak | Seg.2 | 2,2 | | 0.0437 |
| | | Mut4-Seg.2 | 2,2 | | 0.0075 |
| | | WT | 2,2 | | 0.0023 |
| **3J** | Multiple t test adjusted with Holm-Sidak | Hp1bp3 | 4,4 | | 0.0062 |
| | | Cx3cr1 | 4,4 | | 0.035 |
| | Unpaired t test | | 9,9 | | 0.0014 |
| | Unpaired t test | | 7,7 | | 0.0133 |
| **4B** | Unpaired t test | mEPSC amplitude | 8,7 | | 0.013 |
| **4H** | Unpaired t test | Spines | 8,7 | | 0.0149 |
| | Unpaired t test | Mushroom | 8,7 | | 0.0015 |
| **4L** | Two-way ANOVA, Tukey's multiple comparisons test | - | 3,20 (Fn,Fd) | F=14.34 | <0.0001 |
| | | M-shCon+N-shCon vs M-shCon+N-shCpeb1 | 6,6 | | 0.0086 |
| | | M-shCon+N-shCon vs M-shCpeb1+N-shCon | 6,6 | | 0.0409 |
| | | M-shCon+N-shCon vs M-shCpeb1+N-shCpeb1 | 6,6 | | <0.0001 |
| | | M-shCon+N-shCpeb1 vs M-shCpeb1+N-shCpeb1 | 6,6 | | 0.0405 |
| | | M-shCpeb1+N-shCon vs M-shCpeb1+N-shCpeb1 | 6,6 | | 0.0085 |
| **4M** | Two-way ANOVA, Tukey's multiple | - | 3,20 (Fn,Fd) | F=22.56 | <0.0001 |
| | | M-shCon+N-shCon vs M-shCon+N-shCpeb1 | 6,6 | | 0.0004 |

| | | | | | |
|---|---|---|---|---|---|
| | comparisons test | M-shCon+N-shCon vs M-shCpeb1+N-shCon | 6,6 | | 0.0027 |
| | | M-shCon+N-shCon vs M-shCpeb1+N-shCpeb1 | 6,6 | | <0.0001 |
| | | M-shCon+N-shCpeb1 vs M-shCpeb1+N-shCpeb1 | 6,6 | | 0.0243 |
| | | M-shCpeb1+N-shCon vs M-shCpeb1+N-shCpeb1 | 6,6 | | 0.0035 |
| **4P** | Two-way ANOVA, Tukey's multiple comparisons test | - | 3,35 (Fn,Fd) | F=17.92 | <0.0001 |
| | | M-shCon+N-shCon vs M-shCon+N-shCpeb1 | 10,9 | | 0.0008 |
| | | M-shCon+N-shCon vs M-shCpeb1+N-shCon | 10,10 | | 0.0006 |
| | | M-shCon+N-shCon vs M-shCpeb1+N-shCpeb1 | 10,10 | | <0.0001 |
| | | M-shCon+N-shCpeb1 vs M-shCpeb1+N-shCpeb1 | 9,10 | | 0.0407 |
| | | M-shCpeb1+N-shCon vs M-shCpeb1+N-shCpeb1 | 10,10 | | 0.0309 |
| **5B** | Multiple t test adjusted with Holm-Sidak | Session 2 | 6,7 | | 0.002412 |
| **5D** | Unpaired t test | mEPSC amplitude | 6,7 | | 0.0031 |
| **5H** | Unpaired t test | Spines | 10,10 | | <0.0001 |
| | Unpaired t test | Mushroom | 10,10 | | <0.0001 |
| **5I** | Two-way ANOVA, Tukey's multiple comparisons test | - | 2,66 (Fn,Fd) | F=114.2 | <0.0001 |
| | | Session 1 | | | |
| | | Cpeb1 WT+Scr vs Cpeb1 cKO+Scr | 7,5 | | <0.0001 |
| | | Cpeb1 cKO+Scr vs Cpeb1 WT+Sh-Hp1bp3 | 5,7 | | <0.0001 |
| | | Cpeb1 cKO+Scr vs Cpeb1 cKO+Sh-Hp1bp3 | 5,7 | | <0.0001 |
| | | Session 2 | | | |
| | | Cpeb1 WT+Scr vs Cpeb1 cKO+Scr | 7,5 | | 0.0016 |
| | | Cpeb1 WT+Scr vs | 7,7 | | 0.0496 |

| | | | | | |
|---|---|---|---|---|---|
| | | Cpeb1WT+Sh-Hp1bp3 | | | |
| | | Cpeb1 cKO+Scr vs Cpeb1 WT+Sh-Hp1bp3 | 5,7 | | <0.0001 |
| | | Cpeb1 cKO+Scr vs Cpeb1 cKO+Sh-Hp1bp3 | 5,7 | | 0.0039 |
| | | Cpeb1 WT+Sh-Hp1bp3 vs Cpeb1cKO+Sh-Hp1bp3 | 7,7 | | 0.0233 |
| **5L** | One-way ANOVA, Tukey's multiple comparisons test | - | 1.735,8.675 (Fn,Fd) | F=52.64 | <0.0001 |
| | | Cpeb1 WT+Scr vs Cpeb1 cKO+Scr | 6,6 | | 0.0022 |
| | | Cpeb1 WT+Scr vs Cpeb1WT+Sh-Hp1bp3 | 6,6 | | 0.0038 |
| | | Cpeb1 cKO+Scr vs Cpeb1 WT+Sh-Hp1bp3 | 6,6 | | 0.0006 |
| | | Cpeb1 cKO+Scr vs Cpeb1 cKO+Sh-Hp1bp3 | 6,6 | | 0.0336 |
| | | Cpeb1 WT+Sh-Hp1bp3 vs Cpeb1cKO+Sh-Hp1bp3 | 6,6 | | 0.0013 |
| **5M** | One-way ANOVA, Tukey's multiple comparisons test | - | 3,20 (Fn,Fd) | F=18.17 | <0.0001 |
| | | Cpeb1 WT+Scr vs Cpeb1 cKO+Scr | 6,6 | | 0.012 |
| | | Cpeb1 WT+Scr vs Cpeb1WT+Sh-Hp1bp3 | 6,6 | | 0.0051 |
| | | Cpeb1 cKO+Scr vs Cpeb1 WT+Sh-Hp1bp3 | 6,6 | | <0.0001 |
| | | Cpeb1 cKO+Scr vs Cpeb1 cKO+Sh-Hp1bp3 | 6,6 | | 0.0479 |
| | | Cpeb1 WT+Sh-Hp1bp3 vs Cpeb1cKO+Sh-Hp1bp3 | 6,6 | | 0.0012 |
| **5P** | One-way ANOVA, Tukey's multiple comparisons test | - | 2.085,12.51 (Fn,Fd) | F=21.4 | <0.0001 |
| | | Cpeb1 WT+Scr vs Cpeb1 cKO+Scr | 7,7 | | 0.0423 |
| | | Cpeb1 WT+Scr vs Cpeb1WT+Sh-Hp1bp3 | 7,7 | | 0.0119 |
| | | Cpeb1 cKO+Scr vs Cpeb1 WT+Sh-Hp1bp3 | 7,7 | | 0.0011 |
| | | Cpeb1 cKO+Scr vs Cpeb1 cKO+Sh-Hp1bp3 | 7,7 | | 0.0039 |
| | | Cpeb1 WT+Sh-Hp1bp3 vs | 7,7 | | 0.0385 |

| | | | | |
|---|---|---|---|---|
| | | Cpeb1cKO+Sh-Hp1bp3 | | |
| **6D** | Multiple t test adjusted with Holm-Sidak | pre-let-7a | 4,4 | 0.0224 |
| | | pre-miR-122 | 4,4 | 0.0301 |
| | | pre-miR-130b | 4,4 | 0.0047 |
| | | pre-miR- 146a | 4,4 | 0.0394 |
| | | pre-miR-28a | 4,4 | 0.0068 |
| | | pre-miR-30a | 4,4 | 0.0096 |
| | | pre-miR-335 | 4,4 | 0.0011 |
| | | pre-miR-342 | 4,4 | 0.0027 |
| | | pre-miR-433 | 4,4 | 0.0022 |
| | | pre-miR-7a | 4,4 | 0.001 |
| **6H** | Multiple t test adjusted with Holm-Sidak | Hp1bp3 | 4,4 | 0.023 |
| | | Gnal | 4,4 | 0.0111 |
| | | Rem2 | 4,4 | <0.0001 |
| | | Gria1 | 4,4 | <0.0001 |
| | | Gria2 | 4,4 | 0.0066 |
| **6I** | Multiple t test adjusted with Holm-Sidak | Gnal | 4,4 | 0.0029 |
| | | Rem2 | 4,4 | 0.0368 |
| | | Gria1 | 4,4 | 0.0043 |
| | | Gria2 | 4,4 | 0.0026 |
| **6J** | Two-way ANOVA, Tukey's multiple comparisons test | - | 2.426,7.279 (Fn,Fd) | <0.0001 |
| | | Gnal | | |
| | | Cpeb1 WT+Scr vs Cpeb1 WT+Sh-Hp1bp3 | 4,4 | 0.0107 |
| | | Cpeb1 WT+Scr vs Cpeb1 cKO+Scr | 4,4 | 0.0014 |
| | | Cpeb1cKO+Scr vs Cpeb1 WT+Sh-Hp1bp3 | 4,4 | <0.0001 |
| | | Cpeb1 WT+Sh-Hp1bp3 vs Cpeb1cKO+Sh-Hp1bp3 | 4,4 | 0.0045 |
| | | Cpeb1 cKO+Scr vs Cpeb1 cKO+Sh-Hp1bp3 | 4,4 | 0.02 |
| | | Rem2 | | |
| | | Cpeb1 WT+Scr vs Cpeb1 WT+Sh-Hp1bp3 | 4,4 | 0.0014 |
| | | Cpeb1 WT+Scr vs Cpeb1 cKO+Scr | 4,4 | 0.0167 |
| | | Cpeb1cKO+Scr vs Cpeb1 WT+Sh-Hp1bp3 | 4,4 | 0.0007 |
| | | Cpeb1 WT+Sh-Hp1bp3 vs Cpeb1cKO+Sh-Hp1bp3 | 4,4 | 0.0016 |

| | | | | | |
|---|---|---|---|---|---|
| | | Cpeb1 cKO+Scr vs Cpeb1 cKO+Sh-Hp1bp3 | 4,4 | | 0.011 |
| | | Gria1 | | | |
| | | Cpeb1 WT+Scr vs Cpeb1 WT+Sh-Hp1bp3 | 4,4 | | 0.0044 |
| | | Cpeb1 WT+Scr vs Cpeb1 cKO+Scr | 4,4 | | 0.012 |
| | | Cpeb1cKO+Scr vs Cpeb1 WT+Sh-Hp1bp3 | 4,4 | | 0.0057 |
| | | Cpeb1 WT+Sh-Hp1bp3 vs Cpeb1cKO+Sh-Hp1bp3 | 4,4 | | 0.0174 |
| | | Cpeb1 cKO+Scr vs Cpeb1 cKO+Sh-Hp1bp3 | 4,4 | | 0.0017 |
| | | Gria2 | | | |
| | | Cpeb1 WT+Scr vs Cpeb1 WT+Sh-Hp1bp3 | 4,4 | | 0.0146 |
| | | Cpeb1 WT+Scr vs Cpeb1 cKO+Scr | 4,4 | | 0.0155 |
| | | Cpeb1cKO+Scr vs Cpeb1 WT+Sh-Hp1bp3 | 4,4 | | 0.0062 |
| | | Cpeb1 WT+Sh-Hp1bp3 vs Cpeb1cKO+Sh-Hp1bp3 | 4,4 | | 0.0234 |
| | | Cpeb1 cKO+Scr vs Cpeb1 cKO+Sh-Hp1bp3 | 4,4 | | 0.0145 |
| **7B** | Multiple t test adjusted with Holm-Sidak | Session 1 | 4,4 | | 0.03628 |
| | | Session 2 | 4,4 | | 0.00622 |
| **7E** | Two-way ANOVA, Tukey's multiple comparisons test | - | 1.206,4.822 (Fn,Fd) | F=42.53 | 0.0013 |
| | | Session 1 | | | |
| | | Scr+Con vs ShCpeb1+Con | 4,4 | | 0.0144 |
| | | Scr+OE-Cx3cr1 vs ShCpeb1+Con | 4,4 | | 0.0017 |
| | | ShCpeb1+Con vs ShCpeb1+OE-Cx3cr1 | 4,4 | | 0.0189 |
| | | Session 2 | | | |
| | | Scr+Con vs Scr+OE-Cx3cr1 | 4,4 | | 0.0278 |
| | | Scr+Con vs ShCpeb1+Con | 4,4 | | 0.0089 |
| | | Scr+OE-Cx3cr1 vs ShCpeb1+Con | 4,4 | | 0.0009 |
| | | Scr+OE-Cx3cr1 vs ShCpeb1+OE-Cx3cr1 | 4,4 | | 0.022 |

| | | | | | |
|---|---|---|---|---|---|
| | | ShCpeb1+Con vs ShCpeb1+OE-Cx3cr1 | 4,4 | | 0.0151 |
| 7J | Unpaired t test | | 6,6 | | 0.0264 |
| 7L | Unpaired t test | Xaf1/Iba1 colocalized pixels | 6,6 | | <0.0001 |
| 7M | Unpaired t test | Xaf1 | 6,6 | | 0.0033 |
| 8C | Unpaired t test | | 7,7 | | 0.0011 |
| 8E | Unpaired t test | | 5,5 | | <0.0001 |
| | Unpaired t test | | 4,4 | | 0.0014 |
| 8F | One-way ANOVA, Tukey's multiple comparisons test | - | 3,12 (Fn,Fd) | F=29.09 | <0.0001 |
| | | M-shCon+N-shCon vs M-shCpeb1+N-shCon | 4,4 | | 0.0006 |
| | | M-shCon+N-shCon vs M-shCpeb1+N-shCpeb1 | 4,4 | | 0.0001 |
| | | M-shCon+N-shCpeb1 vs M-shCpeb1+N-shCon | 4,4 | | 0.0002 |
| | | M-shCon+N-shCpeb1 vs M-shCpeb1+N-shCpeb1 | 4,4 | | <0.0001 |

**Supplementary Fig 1-13**

| Fig | Statistic Methods | Comparison | Number | Statistics | P value |
|---|---|---|---|---|---|
| 1D | One-way ANOVA, Tukey's multiple comparisons test | - | 3,12 (Fn,Fd) | F=9.959 | 0.0014 |
| | | Con (Off) vs gePSI (On) | 4,4 | | 0.0047 |
| | | Con (On) vs gePSI (On) | 4,4 | | 0.004 |
| | | gePSI (Off) vs gePSI (On) | 4,4 | | 0.0029 |
| 1F | One-way ANOVA, Tukey's multiple comparisons test | - | 3,12 (Fn,Fd) | F=6.719 | 0.0065 |
| | | Con (Off) vs gePSI (On) | 4,4 | | 0.0149 |
| | | Con (On) vs gePSI (On) | 4,4 | | 0.0138 |
| | | gePSI (Off) vs gePSI (On) | 4,4 | | 0.0158 |
| 1G | Two-way | - | 6,78 | F=4.716 | 0.0004 |

| | | | | | |
|---|---|---|---|---|---|
| | ANOVA, Tukey's multiple comparisons test | | (Fn,Fd) | | |
| | | Session 2 | | | |
| | | Con (Off) vs gePSI (On) | 6,10 | | <0.0001 |
| | | Con (On) vs gePSI (On) | 6,10 | | <0.0001 |
| | | gePSI (Off) vs gePSI (On) | 8,10 | | <0.0001 |
| **1I** | Unpaired t test | | 4,4 | | 0.0478 |
| **3B** | Two-way ANOVA, Tukey's multiple comparisons test | - | 2,27 (Fn,Fd) | F=23.53 | <0.0001 |
| | | PL | | | |
| | | Normal-ext vs Impaired-ext | 4,4 | | 0.0024 |
| | | IL | | | |
| | | No-ext vs Normal-ext | 4,4 | | 0.0475 |
| | | No-ext vs Impaired-ext | 4,4 | | 0.0083 |
| | | Normal-ext vs Impaired-ext | 4,4 | | <0.0001 |
| **3D** | One-way ANOVA, Tukey's multiple comparisons test | - | 2,12 (Fn,Fd) | F=8.127 | 0.0059 |
| | | No-ext vs Impaired-ext | 5,4 | | 0.0378 |
| | | Normal-ext vs Impaired-ext | 6,4 | | 0.0048 |
| **4G** | Unpaired t test | Scramble vs Sh-Cpeb1 | 4,4 | | 0.003 |
| **6B** | Two-way ANOVA, Tukey's multiple comparisons test | - | 2,8 (Fn,Fd) | F=134.6 | <0.0001 |
| | | Cpeb1 | | | |
| | | Control vs OE-Cpeb1 | 3,3 | | <0.0001 |
| | | Control vs Sh-Cpeb1 | 3,3 | | 0.0032 |
| | | OE-Cpeb1 vs Sh-Cpeb1 | 3,3 | | <0.0001 |
| | | Hp1bp3 | | | |
| | | Control vs OE-Cpeb1 | 3,3 | | 0.0229 |
| | | Control vs Sh-Cpeb1 | 3,3 | | 0.0334 |
| | | OE-Cpeb1 vs Sh-Cpeb1 | 3,3 | | 0.0005 |
| **6E** | Two-way ANOVA, Tukey's multiple comparisons test | - | 2,8 (Fn,Fd) | F=51.43 | <0.0001 |
| | | Cpeb1 | | | |
| | | Control vs OE-Cpeb1 | 3,3 | | 0.037 |
| | | Control vs Sh-Cpeb1 | 3,3 | | 0.0198 |
| | | OE-Cpeb1 vs Sh-Cpeb1 | 3,3 | | 0.0005 |
| | | Cx3cr1 | | | |
| | | Control vs OE-Cpeb1 | 3,3 | | 0.0149 |
| | | Control vs Sh-Cpeb1 | 3,3 | | 0.0088 |
| | | OE-Cpeb1 vs Sh-Cpeb1 | 3,3 | | 0.0001 |
| **6L** | Multiple t | Hp1bp3 | 4,4 | | 0.00689 |

| | | | | | |
|---|---|---|---|---|---|
| | test adjusted with Holm-Sidak | Cx3cr1 | 4,4 | | 0.0498 |
| **9C** | Unpaired t test | | 4,4 | | 0.0005 |
| **9I** | Two-way ANOVA, Tukey's multiple comparisons test | - | 1,6 (Fn,Fd) | F=158.4 | <0.0001 |
| | | Hp1bp3 | | | |
| | | Cpeb1 WT+Scr vs Cpeb1 WT+Sh-Hp1bp3 | 4,4 | | 0.0035 |
| | | Cpeb1 WT+Scr vs Cpeb1 cKO+Scr | 4,4 | | 0.0477 |
| | | Cpeb1 WT+Sh-Hp1bp3 vs Cpeb1cKO+Scr | 4,4 | | 0.0025 |
| | | Cpeb1 WT+Sh-Hp1bp3 vs Cpeb1cKO+Sh-Hp1bp3 | 4,4 | | 0.0278 |
| | | Cpeb1 cKO+Scr vs Cpeb1 cKO+Sh-Hp1bp3 | 4,4 | | 0.0491 |
| | | Cpeb1 | | | |
| | | Cpeb1 WT+Scr vs Cpeb1 cKO+Scr | 4,4 | | 0.0307 |
| | | Cpeb1 WT+Scr vs Cpeb1 cKO+Sh-Hp1bp3 | 4,4 | | 0.0254 |
| | | Cpeb1 WT+Sh-Hp1bp3 vs Cpeb1cKO+Sh-Hp1bp3 | 4,4 | | 0.0363 |
| | | Cpeb1 cKO+Scr vs Cpeb1 cKO+Sh-Hp1bp3 | 4,4 | | 0.0202 |
| **10A** | One-way ANOVA, Tukey's multiple comparisons test | - | 3,12 (Fn,Fd) | F=18.8 | <0.0001 |
| | | let-7a-5p | | | |
| | | Cpeb1 WT+Scr vs Cpeb1 WT+Sh-Hp1bp3 | 4,4 | | 0.0162 |
| | | Cpeb1 WT+Scr vs Cpeb1 cKO+Scr | 4,4 | | 0.0104 |
| | | Cpeb1 WT+Sh-Hp1bp3 vs Cpeb1cKO+Scr | 4,4 | | <0.0001 |
| | | Cpeb1 WT+Sh-Hp1bp3 vs Cpeb1cKO+Sh-Hp1bp3 | 4,4 | | 0.0353 |
| | | Cpeb1 cKO+Scr vs Cpeb1 cKO+Sh-Hp1bp3 | 4,4 | | 0.0048 |
| | | - | 3,12 (Fn,Fd) | F=42.93 | <0.0001 |
| | | miR-122-5p | | | |
| | | Cpeb1 WT+Scr vs Cpeb1 | 4,4 | | 0.0003 |

| | | | | |
|---|---|---|---|---|
| | WT+Sh-Hp1bp3 | | | |
| | Cpeb1 WT+Scr vs Cpeb1 cKO+Scr | 4,4 | | 0.001 |
| | Cpeb1 WT+Sh-Hp1bp3 vs Cpeb1cKO+Scr | 4,4 | | <0.0001 |
| | Cpeb1 WT+Sh-Hp1bp3 vs Cpeb1cKO+Sh-Hp1bp3 | 4,4 | | 0.0004 |
| | Cpeb1 cKO+Scr vs Cpeb1 cKO+Sh-Hp1bp3 | 4,4 | | 0.0008 |
| | - | 3,12 (Fn,Fd) | F=30.6 | <0.0001 |
| | miR-130b-5p | | | |
| | Cpeb1 WT+Scr vs Cpeb1 WT+Sh-Hp1bp3 | 4,4 | | 0.0026 |
| | Cpeb1 WT+Scr vs Cpeb1 cKO+Scr | 4,4 | | 0.0021 |
| | Cpeb1 WT+Sh-Hp1bp3 vs Cpeb1cKO+Scr | 4,4 | | <0.0001 |
| | Cpeb1 WT+Sh-Hp1bp3 vs Cpeb1cKO+Sh-Hp1bp3 | 4,4 | | 0.0143 |
| | Cpeb1 cKO+Scr vs Cpeb1 cKO+Sh-Hp1bp3 | 4,4 | | 0.0004 |
| | - | 3,12 (Fn,Fd) | F=38.2 | <0.0001 |
| | miR-146a-5p | | | |
| | Cpeb1 WT+Scr vs Cpeb1 WT+Sh-Hp1bp3 | 4,4 | | 0.0008 |
| | Cpeb1 WT+Scr vs Cpeb1 cKO+Scr | 4,4 | | 0.001 |
| | Cpeb1 WT+Sh-Hp1bp3 vs Cpeb1cKO+Scr | 4,4 | | <0.0001 |
| | Cpeb1 WT+Sh-Hp1bp3 vs Cpeb1cKO+Sh-Hp1bp3 | 4,4 | | 0.0011 |
| | Cpeb1 cKO+Scr vs Cpeb1 cKO+Sh-Hp1bp3 | 4,4 | | 0.0007 |
| | - | 3,12 (Fn,Fd) | F=23.68 | <0.0001 |
| | miR-28a-5p | | | |
| | Cpeb1 WT+Scr vs Cpeb1 WT+Sh-Hp1bp3 | 4,4 | | 0.0147 |
| | Cpeb1 WT+Scr vs Cpeb1 cKO+Scr | 4,4 | | 0.0024 |
| | Cpeb1 WT+Sh-Hp1bp3 vs | 4,4 | | <0.0001 |

| | | | | |
|---|---|---|---|---|
| | Cpeb1cKO+Scr | | | |
| | Cpeb1 WT+Sh-Hp1bp3 vs Cpeb1cKO+Sh-Hp1bp3 | 4,4 | | 0.0064 |
| | Cpeb1 cKO+Scr vs Cpeb1 cKO+Sh-Hp1bp3 | 4,4 | | 0.0053 |
| | - | 3,12 (Fn,Fd) | F=14.51 | 0.0003 |
| | miR-30a-5p | | | |
| | Cpeb1 WT+Scr vs Cpeb1 WT+Sh-Hp1bp3 | 4,4 | | 0.021 |
| | Cpeb1 WT+Scr vs Cpeb1 cKO+Scr | 4,4 | | 0.0389 |
| | Cpeb1 WT+Sh-Hp1bp3 vs Cpeb1cKO+Scr | 4,4 | | 0.0001 |
| | Cpeb1 WT+Sh-Hp1bp3 vs Cpeb1cKO+Sh-Hp1bp3 | 4,4 | | 0.0471 |
| | Cpeb1 cKO+Scr vs Cpeb1 cKO+Sh-Hp1bp3 | 4,4 | | 0.0173 |
| | - | 3,12 (Fn,Fd) | F=115.7 | <0.0001 |
| | miR-335-5p | | | |
| | Cpeb1 WT+Scr vs Cpeb1 WT+Sh-Hp1bp3 | 4,4 | | <0.0001 |
| | Cpeb1 WT+Scr vs Cpeb1 cKO+Scr | 4,4 | | <0.0001 |
| | Cpeb1 WT+Sh-Hp1bp3 vs Cpeb1cKO+Scr | 4,4 | | <0.0001 |
| | Cpeb1 WT+Sh-Hp1bp3 vs Cpeb1cKO+Sh-Hp1bp3 | 4,4 | | <0.0001 |
| | Cpeb1 cKO+Scr vs Cpeb1 cKO+Sh-Hp1bp3 | 4,4 | | <0.0001 |
| | - | 3,12 (Fn,Fd) | F=47.79 | <0.0001 |
| | miR-342-3p | | | |
| | Cpeb1 WT+Scr vs Cpeb1 WT+Sh-Hp1bp3 | 4,4 | | 0.001 |
| | Cpeb1 WT+Scr vs Cpeb1 cKO+Scr | 4,4 | | 0.0001 |
| | Cpeb1 WT+Sh-Hp1bp3 vs Cpeb1cKO+Scr | 4,4 | | <0.0001 |
| | Cpeb1 WT+Sh-Hp1bp3 vs Cpeb1cKO+Sh-Hp1bp3 | 4,4 | | 0.0002 |
| | Cpeb1 cKO+Scr vs Cpeb1 | 4,4 | | 0.0007 |

| | | | | | |
|---|---|---|---|---|---|
| | | cKO+Sh-Hp1bp3 | | | |
| | | - | 3,12 (Fn,Fd) | F=53.52 | <0.0001 |
| | | miR-433-5p | | | |
| | | Cpeb1 WT+Scr vs Cpeb1 WT+Sh-Hp1bp3 | 4,4 | | 0.0002 |
| | | Cpeb1 WT+Scr vs Cpeb1 cKO+Scr | 4,4 | | 0.0003 |
| | | Cpeb1 WT+Sh-Hp1bp3 vs Cpeb1cKO+Scr | 4,4 | | <0.0001 |
| | | Cpeb1 WT+Sh-Hp1bp3 vs Cpeb1cKO+Sh-Hp1bp3 | 4,4 | | 0.0074 |
| | | Cpeb1 cKO+Scr vs Cpeb1 cKO+Sh-Hp1bp3 | 4,4 | | <0.0001 |
| | | - | 3,12 (Fn,Fd) | F=23.38 | <0.0001 |
| | | miR-7a-5p | | | |
| | | Cpeb1 WT+Scr vs Cpeb1 WT+Sh-Hp1bp3 | 4,4 | | 0.0038 |
| | | Cpeb1 WT+Scr vs Cpeb1 cKO+Scr | 4,4 | | 0.0022 |
| | | Cpeb1 WT+Sh-Hp1bp3 vs Cpeb1cKO+Scr | 4,4 | | <0.0001 |
| | | Cpeb1 WT+Sh-Hp1bp3 vs Cpeb1cKO+Sh-Hp1bp3 | 4,4 | | 0.0051 |
| | | Cpeb1 cKO+Scr vs Cpeb1 cKO+Sh-Hp1bp3 | 4,4 | | 0.0017 |
| **11B** | Unpaired t test | Rem2/NeuN | 6,6 | | 0.0029 |
| | Unpaired t test | Gnal/NeuN | 6,6 | | 0.0187 |
| | Unpaired t test | Gria1/NeuN | 6,6 | | 0.0026 |
| | Unpaired t test | Gria2/NeuN | 6,6 | | 0.0209 |
| **12D** | Unpaired t test | | 4,4 | | 0.0475 |
| **12H** | Two-way ANOVA, Tukey's multiple comparisons test | - | 3,12 (Fn,Fd) | F=74.33 | <0.0001 |
| | | Cx3cr1 | | | |
| | | Scr+Con vs Scr+OE-Cx3cr1 | 4,4 | | <0.0001 |
| | | Scr+Con vs ShCpeb1+Con | 4,4 | | 0.0096 |
| | | Scr+OE-Cx3cr1 vs | 4,4 | | <0.0001 |

| | | | | |
|---|---|---|---|---|
| | | ShCpeb1+Con | | |
| | | Scr+OE-Cx3cr1 vs ShCpeb1+OE-Cx3cr1 | 4,4 | <0.0001 |
| | | ShCpeb1+Con vs ShCpeb1+OE-Cx3cr1 | 4,4 | 0.0062 |
| | | Cpeb1 | | |
| | | Scr+Con vs ShCpeb1+Con | 4,4 | 0.0002 |
| | | Scr+Con vs ShCpeb1+OE-Cx3cr1 | 4,4 | 0.0148 |
| | | Scr+OE-Cx3cr1 vs ShCpeb1+Con | 4,4 | 0.0009 |
| | | Scr+OE-Cx3cr1 vs ShCpeb1+OE-Cx3cr1 | 4,4 | 0.0482 |
| **13D** | Unpaired t test | | 6,6 | 0.0002 |
| | Unpaired t test | | 4,4 | 0.0452 |

**Data S1:RNA and Protein data from the mPFC in Normal-ext and Impaired-ext mice**

**Unedited blot and gel images:**

## Fig. 6G

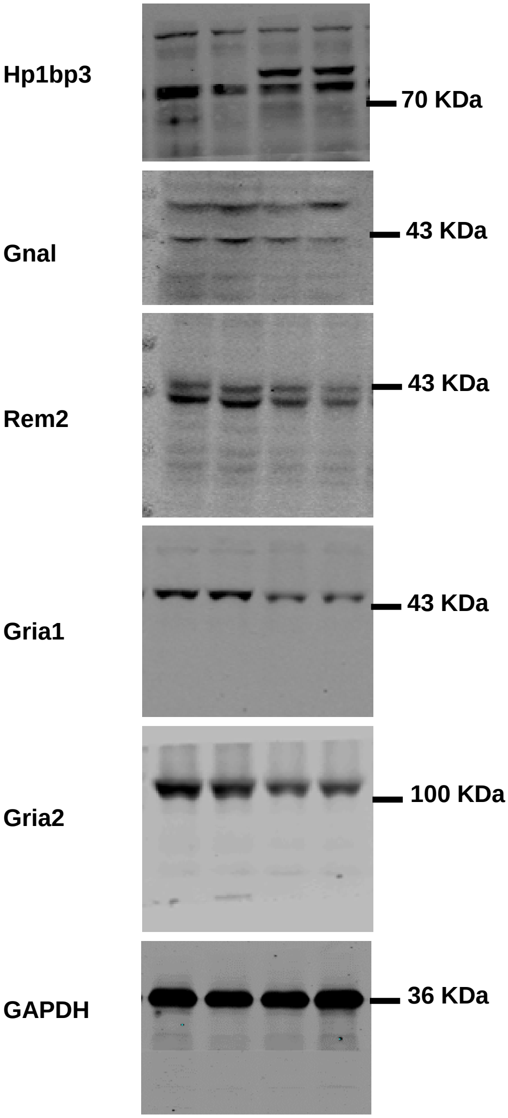

Hp1bp3
70 KDa
Gnal
43 KDa
Rem2
43 KDa
Gria1
43 KDa
Gria2
100 KDa
GAPDH
36 KDa


## Fig. 6I

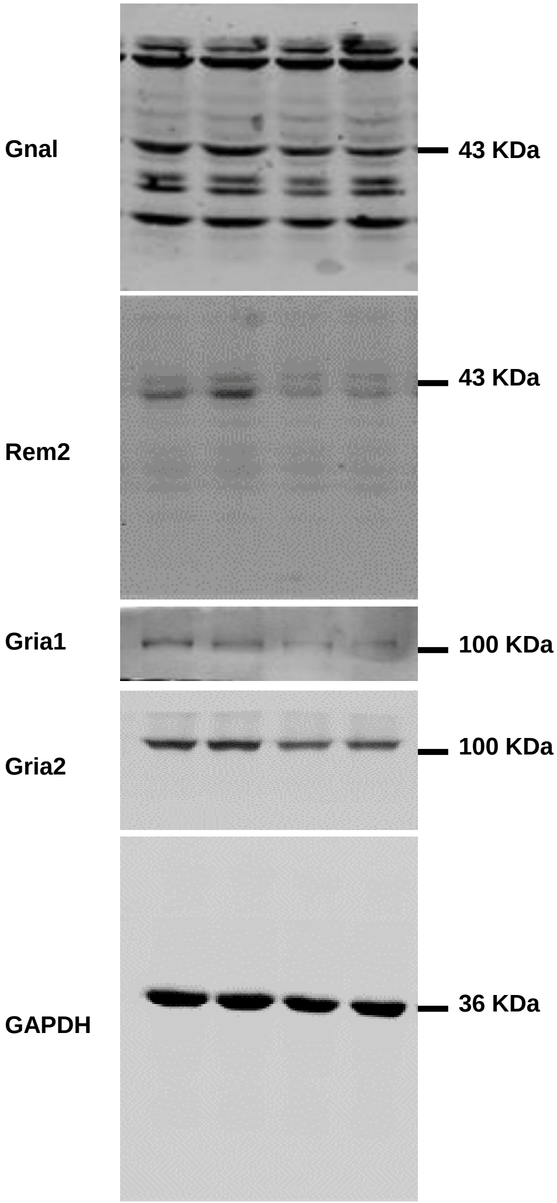

Gnal
43 KDa
Rem2
43 KDa
Gria1
100 KDa
Gria2
100 KDa
GAPDH
36 KDa

# Fig. 6J

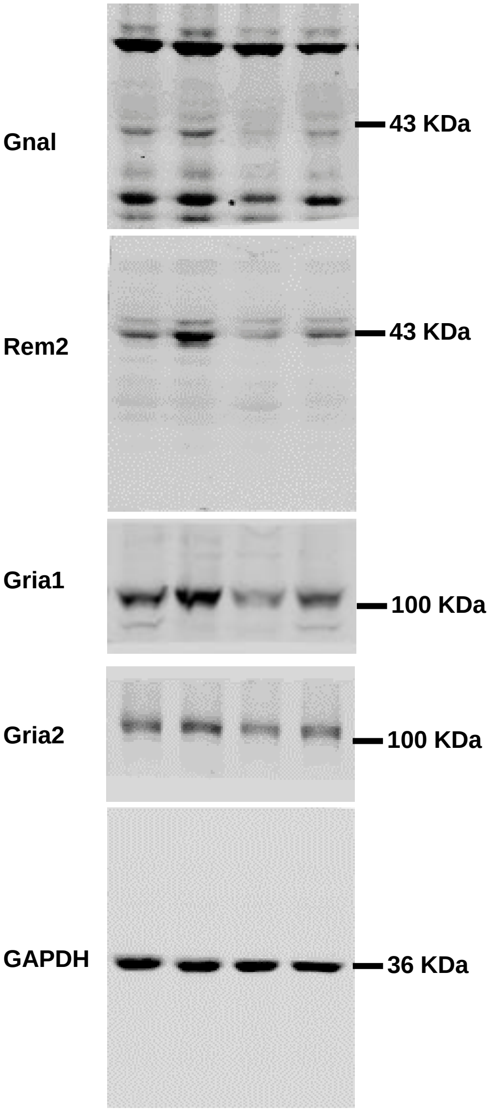


# Fig. S1E

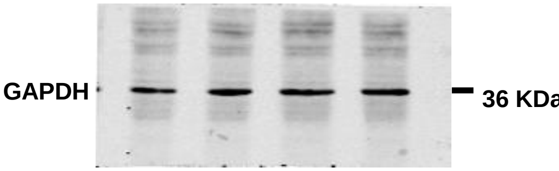


# Fig. S1H

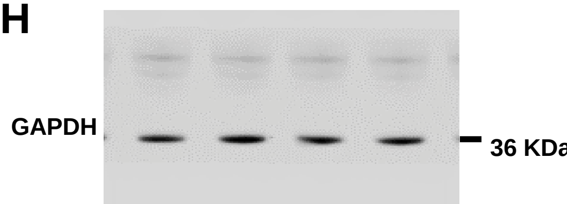


# Fig.S3C

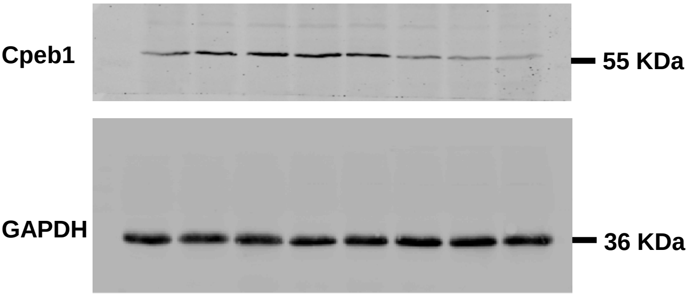


# Fig.S4F

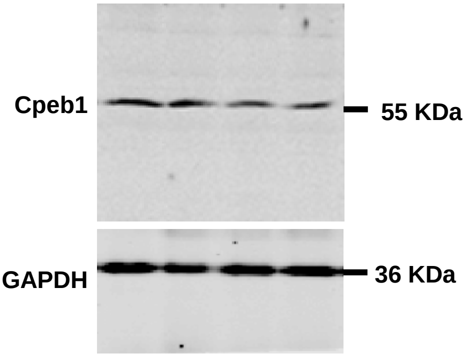

# Fig. S6A

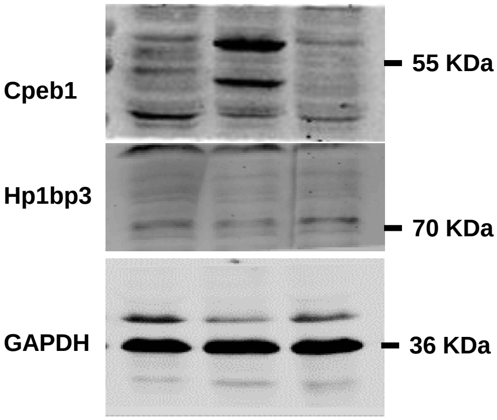


# Fig. S6D

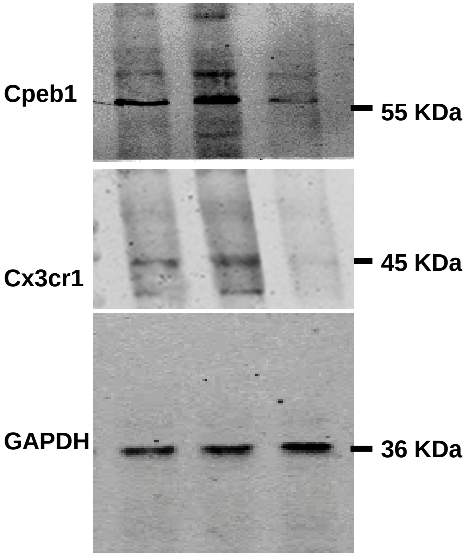


# Fig. S6K

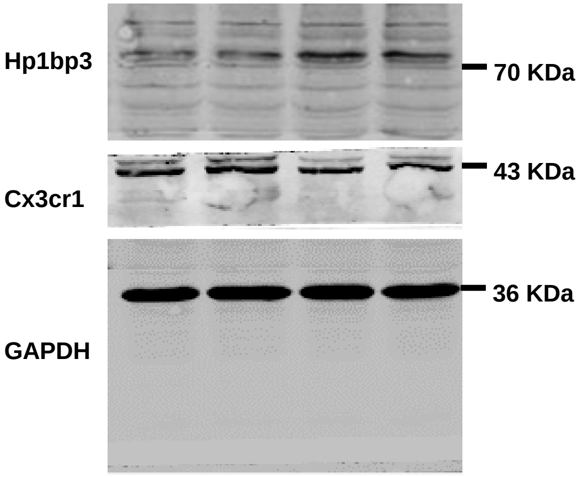


# Fig. S9B

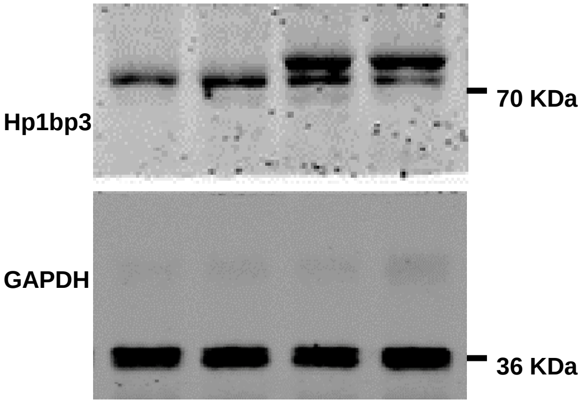

Fig. S9H

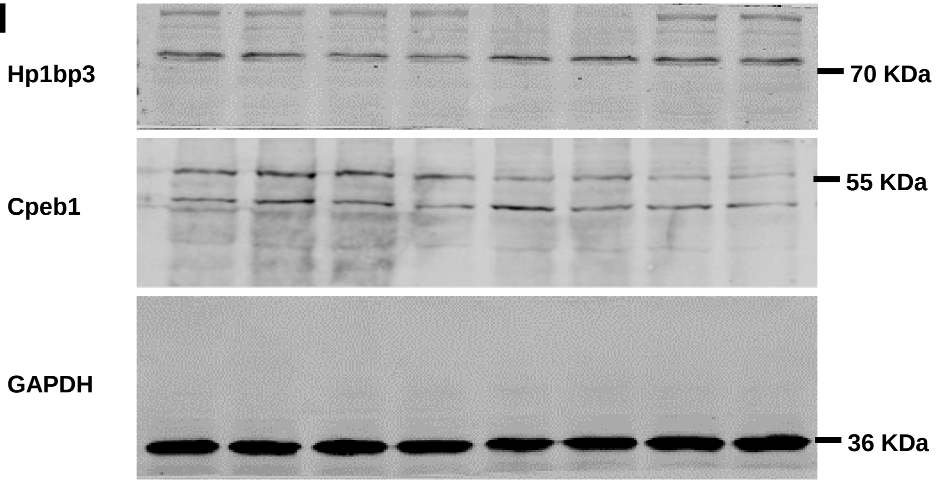


Fig. S12C

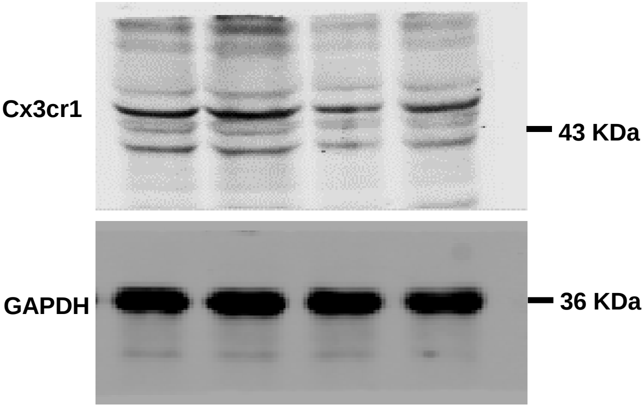


Fig. S12G

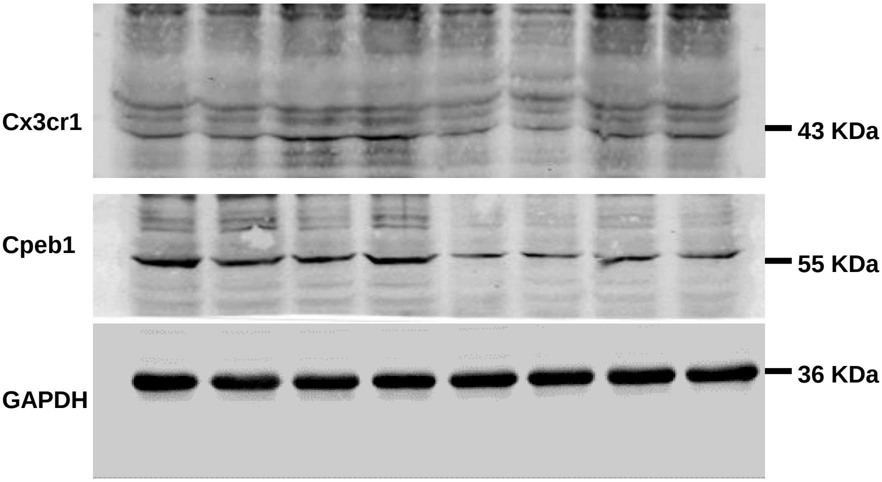

Supplement: Supplementary file 1 — Figs. S1 to S13 Tables S1 to S5 Legend for data S1 Uncropped blot and gel images [file sciadv.adr8687_sm.pdf]
